# Blocking NS3–NS4B interaction inhibits dengue virus in non-human primates

**DOI:** 10.1038/s41586-023-05790-6

**Published:** 2023-03-15

**Authors:** Olivia Goethals, Suzanne J. F. Kaptein, Bart Kesteleyn, Jean-François Bonfanti, Liesbeth Van Wesenbeeck, Dorothée Bardiot, Ernst J. Verschoor, Babs E. Verstrepen, Zahra Fagrouch, J. Robert Putnak, Dominik Kiemel, Oliver Ackaert, Roel Straetemans, Sophie Lachau-Durand, Peggy Geluykens, Marjolein Crabbe, Kim Thys, Bart Stoops, Oliver Lenz, Lotke Tambuyzer, Sandra De Meyer, Kai Dallmeier, Michael K. McCracken, Gregory D. Gromowski, Wiriya Rutvisuttinunt, Richard G. Jarman, Nicos Karasavvas, Franck Touret, Gilles Querat, Xavier de Lamballerie, Laurent Chatel-Chaix, Gregg N. Milligan, David W. C. Beasley, Nigel Bourne, Alan D. T. Barrett, Arnaud Marchand, Tim H. M. Jonckers, Pierre Raboisson, Kenny Simmen, Patrick Chaltin, Ralf Bartenschlager, Willy M. Bogers, Johan Neyts, Marnix Van Loock

**Affiliations:** 1 Janssen Global Public Health, Janssen Pharmaceutica NV, Beerse, Belgium; 2 Department of Microbiology, Immunology and Transplantation, Rega Institute for Medical Research, Laboratory of Virology and Chemotherapy, KU Leuven, Leuven, Belgium; 3 Janssen Research & Development, Janssen Pharmaceutica NV, Beerse, Belgium; 4 Janssen Infectious Diseases Discovery, Janssen-Cilag, Val de Reuil, France; 5 Galapagos, Romainville, France; 6 Cistim Leuven vzw, Leuven, Belgium; 7 Department of Virology, Biomedical Primate Research Centre, Rijswijk, The Netherlands; 8 Viral Diseases Branch, Walter Reed Army Institute of Research, Silver Spring, MD USA; 9 Heidelberg University, Medical Faculty Heidelberg, Department of Infectious Diseases, Molecular Virology, Center for Integrative Infectious Diseases Research, Heidelberg, Germany; 10 Janssen Clinical Pharmacology and Pharmacometrics, Janssen Pharmaceutica NV, Beerse, Belgium; 11 Statistics and Decision Sciences, Janssen Pharmaceutica NV, Beerse, Belgium; 12 Discovery, Charles River Beerse, Beerse, Belgium; 13 Unité des Virus Émergents, Aix-Marseille Université-IRD 190-Inserm 1207, Marseille, France; 14 Centre Armand-Frappier Santé Biotechnologie, Institut National de la Recherche Scientifique, Laval, Quebec Canada; 15 Sealy Institute for Vaccine Sciences, The University of Texas Medical Branch Health, Galveston, TX USA; 16 Galapagos NV, Mechelen, Belgium; 17 Johnson & Johnson Innovation, London, UK; 18 Centre for Drug Design and Discovery (CD3), KU Leuven, Leuven, Belgium; 19 German Centre for Infection Research, Heidelberg Partner Site, Heidelberg, Germany; 20 Global Virus Network (GVN), Baltimore, MD USA

**Keywords:** Drug discovery, Diseases

## Abstract

Dengue is a major health threat and the number of symptomatic infections caused by the four dengue serotypes is estimated to be 96 million^1^ with annually around 10,000 deaths^2^. However, no antiviral drugs are available for the treatment or prophylaxis of dengue. We recently described the interaction between non-structural proteins NS3 and NS4B as a promising target for the development of pan-serotype dengue virus (DENV) inhibitors^3^. Here we present JNJ-1802—a highly potent DENV inhibitor that blocks the NS3–NS4B interaction within the viral replication complex. JNJ-1802 exerts picomolar to low nanomolar in vitro antiviral activity, a high barrier to resistance and potent in vivo efficacy in mice against infection with any of the four DENV serotypes. Finally, we demonstrate that the small-molecule inhibitor JNJ-1802 is highly effective against viral infection with DENV-1 or DENV-2 in non-human primates. JNJ-1802 has successfully completed a phase I first-in-human clinical study in healthy volunteers and was found to be safe and well tolerated^4^. These findings support the further clinical development of JNJ-1802, a first-in-class antiviral agent against dengue, which is now progressing in clinical studies for the prevention and treatment of dengue.

## Abstract (web summary)

JNJ-1802—a highly potent dengue virus inhibitor—blocks the NS3–NS4B interaction within the viral replication complex, and is highly effective against viral infection with DENV-1 or DENV-2 in non-human primates.

## Main

An estimated 390 million DENV infections occur each year^1^, resulting in 96 million symptomatic infections^1^ and 10,000 deaths^2^, spread across 125 countries^2^ and accounting for 1.1 to 2.4 million disability-adjusted life years^2,5^. Furthermore, the World Health Organization has reported that dengue is the most rapidly spreading mosquito-borne viral disease worldwide with around half of the world’s population at risk of contracting dengue^1,6^. As a result of urbanization, globalization, a lack of effective mosquito control and climate change, the geographical range of the disease continues to expand^7,8^, leaving up to 60% of the world population at risk of infection by 2080 according to predictions^9^. Dengue is caused by any of the four DENV serotypes (DENV-1–4) and each of them can induce the full spectrum of disease^10,11^. A second infection with a different DENV serotype can result in more severe disease. The underlying mechanism for this is assumed to be antibody-dependent enhancement (ADE)^12^. Vaccine development has been challenging, requiring the induction of a balanced immune response by the vaccine against all four serotypes. Until recently, only one vaccine (Dengvaxia, Sanofi-Pasteur) was approved and available in several countries, but with restrictions on its use^13^. For example, the US Food and Drug Administration restricted Dengvaxia’s use to individuals aged 9–16 years with laboratory-confirmed previous DENV infection living in endemic areas^14–16^. The more recent QDENGA vaccine (Takeda) received its first approval in Indonesia^17^ in August 2022, in Europe^18^ in December 2022 and in the United Kingdom^19^ in February 2023. Currently, no dengue-specific treatment exists. The development of pan-serotype DENV inhibitors has proven to be challenging as exemplified by the discontinuation of various drug discovery programmes^20,21^. We recently published our findings regarding JNJ-A07, a highly potent DENV inhibitor, with a previously undescribed mechanism of action that targets the DENV non-structural protein NS4B and thereby prevents the interaction between NS4B and NS3 (ref. ^3^). Here, we characterize a compound from the same chemical series, namely JNJ-1802, which was selected over JNJ-A07 on the basis of its improved preclinical safety profile and which is currently progressing through clinical development. We describe the excellent preclinical profile of JNJ-1802 with potent in vitro antiviral activity, a high barrier to resistance, pan-serotype DENV potency in mice and substantial in vivo efficacy against DENV infection in non-human primates (NHPs).

## Subnanomolar activity for JNJ-1802

**Fig. 1:**
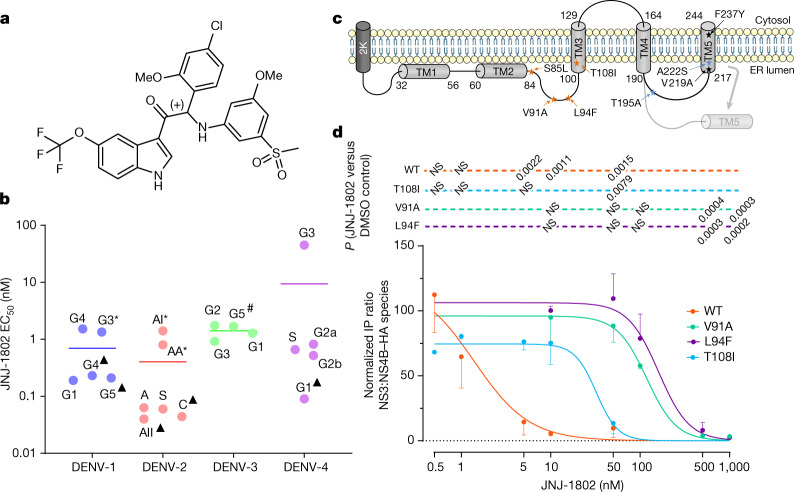
The molecular structure, in vitro pan-genotype and -serotype activity, and mechanism of action of JNJ-1802. **a**, The molecular structure of JNJ-1802. **b**, In vitro antiviral activity in Vero E6 cells against a panel of clinical isolates^23^. Data are mean EC_50_ values. The asterisk and hash symbols indicate that the DENV strain carries a T108I (*) or T108A (#) mutation in NS4B. Where indicated by a triangle, the mean EC_50_ in Vero E6 cells was calculated by setting the values below the 0.04 nM threshold at 0.04 nM. A, American; AA, Asian American; AI, Asian I; AII, Asian II; C, cosmopolitan; S, sylvatic. **c**, Schematic of the DENV NS4B membrane topology^50,51^. JNJ-1802-selected resistance mutations in orange were present in at least 99% of the quasispecies at the end point (passage 42 (sample A); passage 50 (sample B)). Mutations in black were present in less than 50% of the quasispecies at the end point. Mutations in blue appeared transiently and had disappeared at the end of the experiment. The diagram was created in part using the Servier Medical Art library (https://smart.servier.com/). **d**, JNJ-1802 prevents DENV NS3–NS4B interaction. Three independent co-immunoprecipitation experiments were performed to establish the JNJ-1802 dose–response curve for the NS3–NS4B interaction. Representative western blots are shown in Extended Data Fig. 2a,b. Signal intensity ratios were determined as described in the Methods. Data are mean ± s.e.m. For the comparison of NS3:NS4B–HA ratios between JNJ-1802 treated samples and DMSO control, *P* values were calculated using repeated-measures one-way analysis of variance (ANOVA) with subsequent Dunnett’s multiple-comparisons test; NS, not significant. EC_50_, 50% effective concentration; DMSO, dimethyl sulfoxide; IP, immunoprecipitation; TM, transmembrane. Source Data

JNJ-1802 (Fig. 1a) exerts picomolar to nanomolar antiviral potency, with 50% mean effective concentrations (EC_50_) ranging from 0.059 nM to 1.24 nM against two DENV-2 laboratory strains in an African green monkey kidney cell line (Vero), a human hepatoma cell line (Huh-7), a mosquito cell line (C6/36) and a human monocytic cell line expressing dendritic cell-specific intercellular adhesion molecule-3-grabbing non-integrin (THP-1/DC-SIGN), the latter being one of the primary target cells of DENV^22^ (Table 1). The mean protein-binding adjusted EC_50_ for JNJ-1802 against DENV-2, measured in Vero cells in the presence of 50% human serum, is 1.40 nM, 24-fold higher than without 50% human serum (Table 1). Pan-genotype and pan-serotype picomolar to low nanomolar activity (mean EC_50_ ranging from <0.04 nM to 1.8 nM) was demonstrated against a panel of 20 DENV strains that represent the diversity of genotypes within the four serotypes^23^, except against the DENV-4 genotype 3 Thailand strain, for which a mean EC_50_ value of 45 nM was obtained (Fig. 1b and Supplementary Table 1). JNJ-1802 is highly specific for DENV and is inactive against a panel of unrelated DNA and RNA viruses. The compound inhibits the replication of other flaviviruses (that is, West Nile Virus (WNV), Japanese encephalitis virus (JEV) and Zika virus (ZIKV)), with mean EC_50_ values ranging from 0.25 µM to 1.1 µM, which is more than 4,000‑fold higher than the EC_50_ value of JNJ-1802 for DENV‑2/16681 (EC_50_ = 0.059 nM) (Extended Data Table 1).

**Table 1:** JNJ-1802 shows antiviral activity and limited cellular toxicity in DENV-infected Vero, C6/36, Huh-7 and THP-1/DC-SIGN cells

		Antiviral activity				Cytotoxicity		SI^a^
Cell line	Virus	EC_50_ (nM)	*n*	EC_90_ (nM)	*n*	CC_50_ (µM)	*n*	
Vero	DENV-2/16681/eGFP	0.059 ± 0.030	16	0.161 ± 0.083	18	2.61 ± 0.47	10	44,000
Vero with 50% HS	DENV-2/16681/eGFP	1.40 ± 0.30^b^	3	4.8 ± 1.4^b^	3	>1.50 ± 0.87	3	>1,100
Vero	DENV-2/RL	0.062 ± 0.019	3	0.275 ± 0.081	3	>2.26 ± 0.45	3	>37,000
C3/36	DENV-2/RL	1.24 ± 0.14	2	6.5 ± 1.0	2	14.4 ± 1.1	2	12,000
Huh-7	DENV-2/16681/eGFP	0.135 ± 0.077	4	0.53 ± 0.31	4	12.70 ± ND	1	94,000
THP-1/DC-SIGN	DENV-2/16681/eGFP	0.173 ± 0.043	6	0.283 ± 0.056	6	>0.50 ± 0.00	6	>2,900

For EC_50_, EC_90_ and 50% cytotoxic concentration (CC_50_), data are mean ± s.d. HS, human serum; ND, not determined; *n* values indicate the number of independent experiments.

^a^Selectivity index (SI) = mean CC_50_/mean EC_50_.

^b^Protein-binding-adjusted EC_50_ or EC_90_.

Source Data

## JNJ-1802 targets the DENV NS4B protein

**Extended Data Fig. 1:**
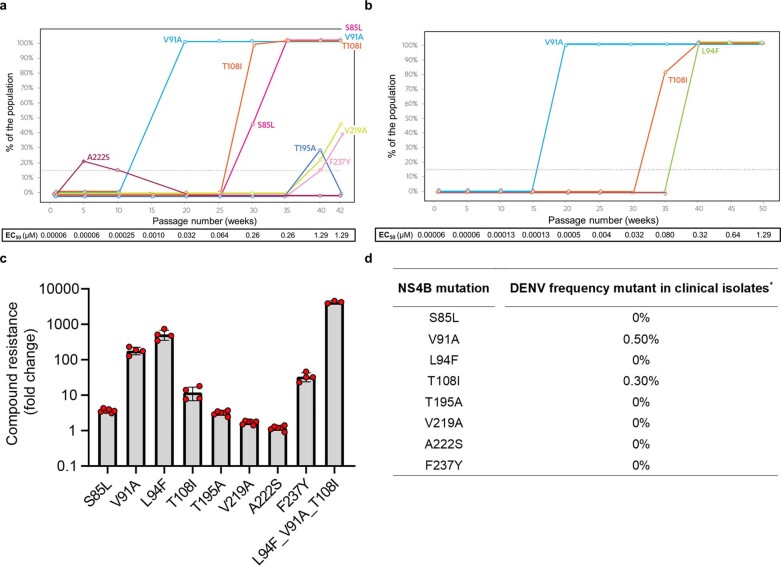
*In vitro* resistance selection profile of JNJ-1802 against DENV-2. **a, b**, The dynamics of emerging mutations were studied using whole virus genome sequencing in two independent experiments (Experiment A (**a**) and experiment B (**b**)). Each coloured line shows the kinetics of appearance of a particular mutation during virus passaging in the presence of JNJ-1802; each mutation is shown in the same colour over the different experiments. Emerging mutations were defined as amino acid changes compared to the start virus (DENV-2/RL), which were not present in the viruses that were passaged in parallel without any drug exposure. Whole genome sequencing was performed on DENV variants harvested at every 5^th^ passage (P) and at the end of the experiments (i.e., P42 for experiment A and P50 for experiment B). One passage represents a one-week time span. The dotted line represents the cut off (15%) for the detection of variants compared with wild-type in the virus population. The increasing EC_50_ values, as determined by microscopic evaluation of virus-induced CPE, are depicted below the graphs. **c**, Level of compound resistance conferred by NS4B mutations in DENV-2/16681 induced by JNJ-1802. Fold change (mean ± standard deviation) in compound resistance is calculated as the EC_50_ of the compound against the mutant sub-genomic DENV-2/16681 reporter replicon divided by the EC_50_ of the compound against wild-type sub-genomic DENV-2/16681 reporter replicon. Data are from at least three independent experiments. **d**, Natural occurrence of the NS4B mutations in clinical isolates. *The natural occurrence of the mutations was retrieved from the Virus Pathogen Resource database (www.viprbrc.org; accessed in May 2020). Prevalence values of ≤0.1% are not shown. CPE, cytopathic effect; EC_50_, 50% effective concentration. Source Data

JNJ-1802-resistant variants were selected in two independent experiments (A and B) by passaging DENV-2 in the presence of gradually increasing concentrations of JNJ-1802 (Extended Data Fig. 1a,b). While the first persistent mutation V91A was selected within NS4B from week 20 onwards, it was insufficient for complete viral breakthrough. Forty weeks or more were needed to enable the virus to grow in the presence of 1.29 µM JNJ-1802, corresponding to more than 20,000-fold the EC_50_ of JNJ-1802 against this virus. Several mutations within NS4B were identified by whole-genome sequencing of the resistant variants (Fig. 1c), but no persistent mutations were identified in NS3. In study A, NS4B mutations S85L, V91A and T108I were present in ≥99% of the viral population and V219A and F237Y in 46% and 39%, respectively, of the viral population at the end of the study (week 42), whereas the mutations A222S and T195A appeared only transiently (Extended Data Fig. 1a). In study B, the mutations L94F, V91A and T108I were observed in ≥99% of the viral population at week 50 (Extended Data Fig. 1b). None of these mutations was present in the viral populations passaged in parallel in the absence of drug pressure. The selected mutations were further assessed for their resistance to JNJ-1802 by inserting them into a subgenomic DENV-2/16681 reporter replicon. V91A and L94F conferred the highest level of resistance and reduced the activity of JNJ-1802 180-fold and 520-fold, respectively. T108I decreased the susceptibility to DENV-2 by 12-fold. Moreover, when the three mutations V91A, L94F and T108I were introduced together into the subgenomic DENV-2 reporter replicon, a 4,200-fold reduction in the activity of JNJ-1802 compared with wild-type (WT) DENV-2 was observed (Extended Data Fig. 1c). Of all observed mutations, only the mutations V91A and T108I were naturally occurring in DENV isolates at a low frequency (≤0.52%) as deduced from the Virus Pathogen Resource (www.viprbrc.org) database (Extended Data Fig. 1d).

## JNJ-1802 blocks the NS3–NS4B interaction

**Extended Data Fig. 2:**
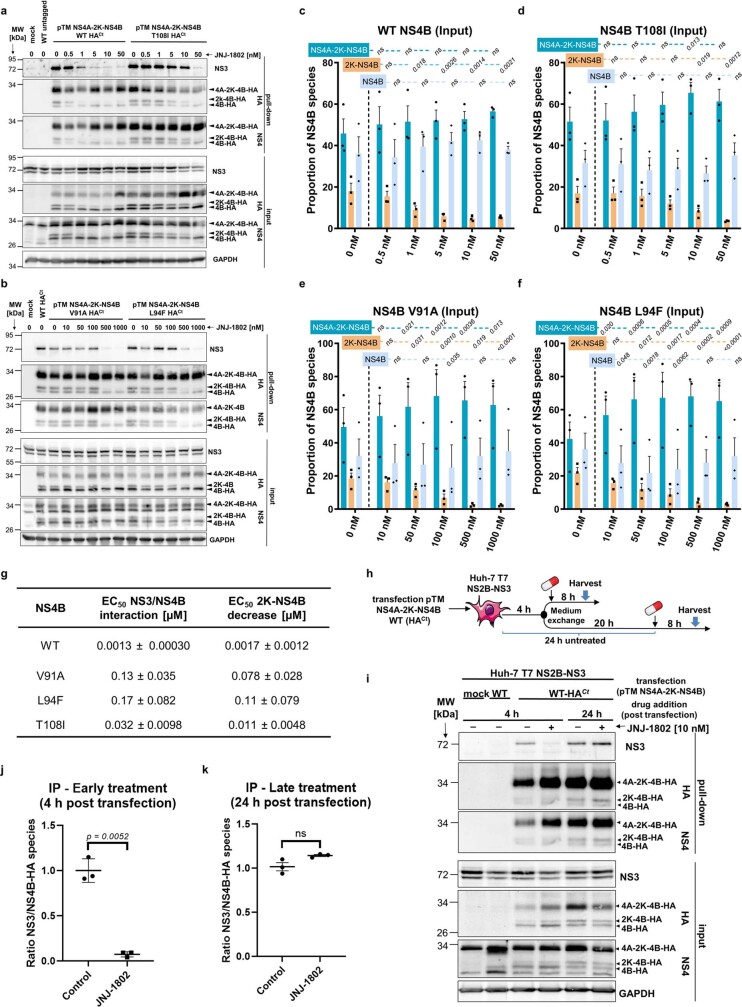
JNJ-1802 prevents DENV NS3-NS4B interaction. **a, b**, Huh-7 cells stably expressing T7 RNA polymerase and DENV-2 NS2B-NS3 were transfected with T7-based expression plasmids encoding NS4A-2K-NS4B(-HA^Ct^) (NS4B corresponding to wild-type [WT] or mutant NS4B). Four hours post-transfection, cells were treated with various concentrations of JNJ-1802 or DMSO, collected 14 h later, lysed and used for HA-specific pull-down and Western blot analysis of captured complexes. Representative Western Blot of the dose-response assays. Samples contain either WT NS4B or NS4B T108I (**a**) and mutant NS4B (V91A or L94F) (**b**). Please note the higher compound concentrations compared to (**a**). **c–f**, Western blot signal intensities of total lysates (input) obtained with an anti-HA-antibody were used to determine the dose-dependent effect of JNJ-1802 on the relative abundance of NS4B-containing species (mean ± standard error of the mean [SEM] of three independent experiments). Repeated measures one-way ANOVA and Dunnett’s multiple comparisons test were used to calculate P values. **g**, EC_50_ values (mean ± SEM) for the NS3-NS4B species ratio (**a, b**) and the change of 2K-NS4B proportion in total lysates (**c–f**) were obtained by fitting four-parameter dose-response curves to the results from each individual experiment. **h**, Experimental setup to investigate blockade of NS3-NS4B interaction with regard to the timing of JNJ-1802 addition. As indicated, cells were treated with 10 nM JNJ-1802 or DMSO, collected 8 h later, lysed and used for HA-specific pull-down and Western blot analysis. The diagram was created in part using the Servier Medical Art Library (https://smart.servier.com/). **i**, Representative Western blot from three independent experiments for the setup in (**h**). **j–k**, Effect of early and late treatment on the NS3/NS4B-HA species ratio in samples after HA-pulldown. Results are the mean and SEM from three independent experiments. Paired two-tailed t-test was applied to calculate P values. Uncropped images of **a,b** and **i** are presented in Supplementary Fig. 1–3. *ns*, not significant; Ct, C-terminal. Source Data

To determine whether JNJ-1802 blocks the interaction between NS3 and NS4B, we transiently expressed the C-terminally haemagglutinin-affinity-tagged NS4A-2K-NS4B precursor (WT or the V91A, L94F and T108I mutants) in cells stably expressing the NS2B–NS3 protease–helicase complex. We observed a loss of interaction between NS3 and WT NS4B at low concentrations (EC_50_ of 0.001 µM) of JNJ-1802, whereas higher JNJ-1802 concentrations (EC_50_ values ranging from 0.03 to 0.2 µM) were required to prevent the interaction between NS3 and NS4B carrying one of the three NS4B resistance mutations (Fig. 1d and Extended Data Fig. 2a,b,g). Moreover, we observed an altered cleavage pattern of the NS4A-2K-4B precursor, which was most apparent for the 2K-NS4B intermediate, for which a dose-dependent decrease was observed (Extended Data Fig. 2a–f). EC_50_ values indicated a correlation between the loss of NS3–NS4B interaction and the decrease in the 2K-NS4B intermediate (Extended Data Fig. 2g).

Similar to JNJ-A07, JNJ-1802 prevents de novo formation of NS3–NS4B complexes but does not disrupt established ones (Extended Data Fig. 2h). This becomes evident from the significant loss (*P* = 0.0052) of NS3–NS4B complexes detected in cells treated with JNJ-1802 4 h after transfection, but not in cells treated 24 h after transfection, when NS3–NS4B complexes had already been established (Extended Data Fig. 2i–k).

## Pan-serotype potency of JNJ-1802 in mice

JNJ-1802 has a favourable pharmacokinetic profile in mice. The compound has low clearance and a moderate volume of distribution resulting in a terminal half-life of 6.2 h. The plasma protein binding in mice was higher than 99.9%. The oral bioavailability was 46% and 59% at 1 and 3 mg per kg, respectively, and increased to more than 100% due to more than dose-proportional pharmacokinetics between 3 and 10 mg per kg. In a separate study, after single oral dosing, JNJ-1802 showed exposures in mice with an area under the plasma concentration versus time curve during 24 h (AUC_0–24h_) ranging from 1,520 up to 120,000 ng h ml−1 for a dose ranging from 1 mg per kg to 30 mg per kg, respectively (Extended Data Table 2a,b).

The prophylactic efficacy of JNJ-1802 against DENV-2 was studied in AG129 mice^24^ using different settings: (1) a 3 day viraemia mouse model with high viral inoculum (10^6^ plaque-forming units (PFU)), (2) an 11 day viraemia mouse model with low viral inoculum (10^2^ PFU) and (3) a lethal DENV challenge mouse model. Moreover, (4) the effect of JNJ-1802 was assessed in a therapeutic setting during a non-lethal (10^2^ PFU) DENV-2 infection.

**Fig. 2:**
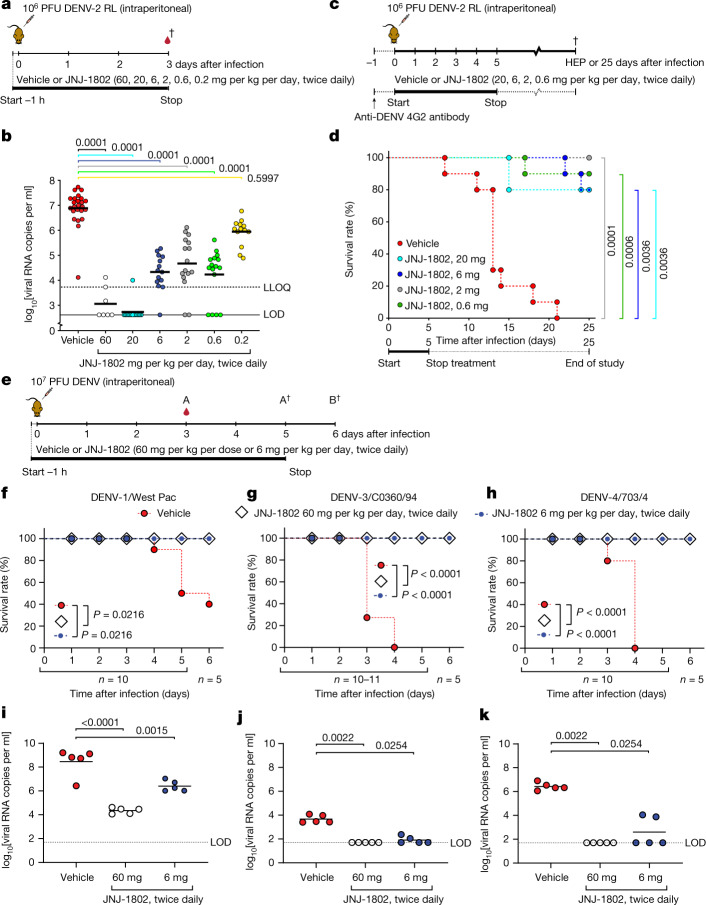
In vivo efficacy of JNJ-1802 (b.i.d.) against DENV-1–4 after infection in AG129 mice. **a**, Schematic of viraemia studies against DENV-2. **b**, The effect of JNJ-1802 on viraemia on day 3 after infection in DENV-2-infected mice treated b.i.d. with 60, 20, 6, 2, 0.6, 0.2 mg per kg per day JNJ-1802 (*n* = 7, 14, 14, 16, 16 and 13, respectively; without anti-flavivirus antibodies), compared with vehicle-treated mice (*n* = 24). Treatment started 1 h before infection. Undetermined *C*_t_ values imputed at a value of 40 (the limit of detection (LOD)) correspond to 2.6 log_10_ viral RNA copies per ml. Pooled data of three independent studies were analysed using two-way ANOVA with Dunnett’s multiple-comparison test. The LLOQ is 3.7 log_10_ viral RNA copies per ml. **c**, Schematic of the survival study. **d**, The effect of JNJ-1802 on survival in mice treated with anti-flavivirus antibodies (clone 4G2) receiving b.i.d. 20, 6, 2, 0.6 or 0 mg per kg per day JNJ-1802 starting 1 h before infection. Data are from a single study (*n* = 10 mice per group). Two-sided Fisher’s exact tests were used on day 25 with Bonferroni’s multiple-comparison test. **e**, Outline of the viraemia/survival studies against DENV-1, DENV-3 and DENV-4. **f**–**k**, The effect of JNJ-1802 on survival (*n* = 10 mice per group, except for DENV-3 vehicle, for which *n* = 11 mice per group) (**f**–**h**) and viraemia (*n* = 5 mice per group) on day 3 after infection (**i**–**k**) in mice challenged with DENV-1/West Pac (**f**,**i**), DENV-3/C0360/94 (**g**,**j**) or DENV-4/703/4 (**h**,**k**), treated b.i.d. with 60, 6 or 0 mg per kg per day JNJ-1802. Treatment started 1 h before infection. The LOD is 1.7 log_10_ viral RNA copies per ml. Two-sided Fisher’s exact tests were used on day 6 (survival) with Bonferroni’s multiple-comparison test. For viraemia, ordinary one-way ANOVA with Dunnett’s multiple-comparison test (DENV-1) and Kruskal–Wallis tests with Dunn’s multiple-comparison test (DENV-3 and DENV-4) were performed. *P* values are shown in the figures. HEP, human end point. For **a**, **c** and **e**, the schematics were adapted from ref. ^3^. The dagger symbol indicates the day of euthanasia. Source Data

**Extended Data Fig. 3:**
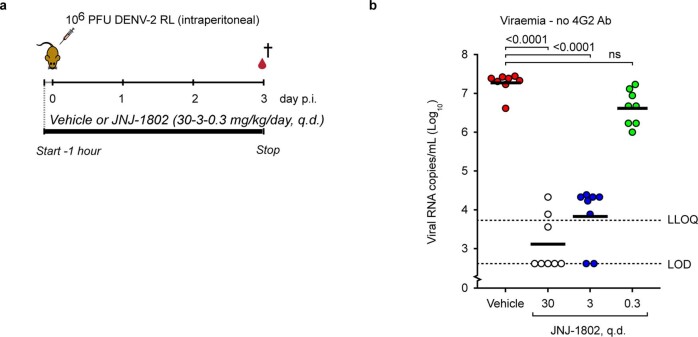
*In vivo* efficacy of JNJ-1802 against DENV-2 infection, dosed once-daily for 3 days. **a**, Schematic outline of a viraemia study using AG129 mice adapted from Kaptein et al.^3^ **b**, Effect of JNJ-1802 on viraemia on Day 3 post-infection (p.i.) in mice orally treated once daily (q.d.) with a dose of 30, 3 or 0.3 (*n* = 8 for all groups) mg/kg/day JNJ-1802, compared to vehicle-treated mice (*n* = 8), with the first dose administered 1 h before infection. Data are from a single study. Undetermined cycle threshold values were imputed at a value of 40 (=limit of detection [LOD]), corresponding to 2.6 log_10_ viral RNA copies/mL. For statistical analysis, ordinary one-way ANOVA was used and Dunnett test to correct for multiple comparisons. p <0.0001 for 30 mg/kg/day versus vehicle; p <0.0001 for 3 mg/kg/day versus vehicle and p = 0.0983 not-significant (ns) for 0.3 mg/kg/day versus vehicle. LLOQ, lower limit of quantification, is 3.7 log_10_ viral RNA copies/mL. 4G2, anti-flavivirus antibody, clone 4G2. Source Data

First, the effect of JNJ-1802 on peak viral load (on day 3 after infection) was studied in mice after challenge with a high viral inoculum of DENV-2 (10^6^ PFU; setting 1) (Fig. 2a). Twice-daily (b.i.d.) dosing with JNJ-1802 for 3 days resulted in a dose-dependent reduction in mean viral RNA (ranging from 3.8 log_10_ to 1.0 log_10_ copies per ml for the dose groups ranging from 60 to 0.2 mg per kg per day), compared with the vehicle. Undetectable viral RNA was observed in four out of seven mice dosed b.i.d. with 60 mg per kg per day and in 13 out of 14 mice dosed b.i.d. with 20 mg per kg per day (Fig. 2b). Once-daily (q.d.) dosing for 3 days (Extended Data Fig. 3a) resulted in low viral RNA levels in DENV-infected mice comparable to b.i.d. dosing, at least for the 30 and 3 mg per kg dosing groups (Extended Data Fig. 3b). Furthermore, five out of eight mice in the 30 mg per kg q.d. dosing group had undetectable viral RNA levels. By contrast, only the 0.3 mg per kg dose was less efficacious when dosed daily compared with b.i.d. dosing (Extended Data Fig. 3b).

**Extended Data Fig. 4:**
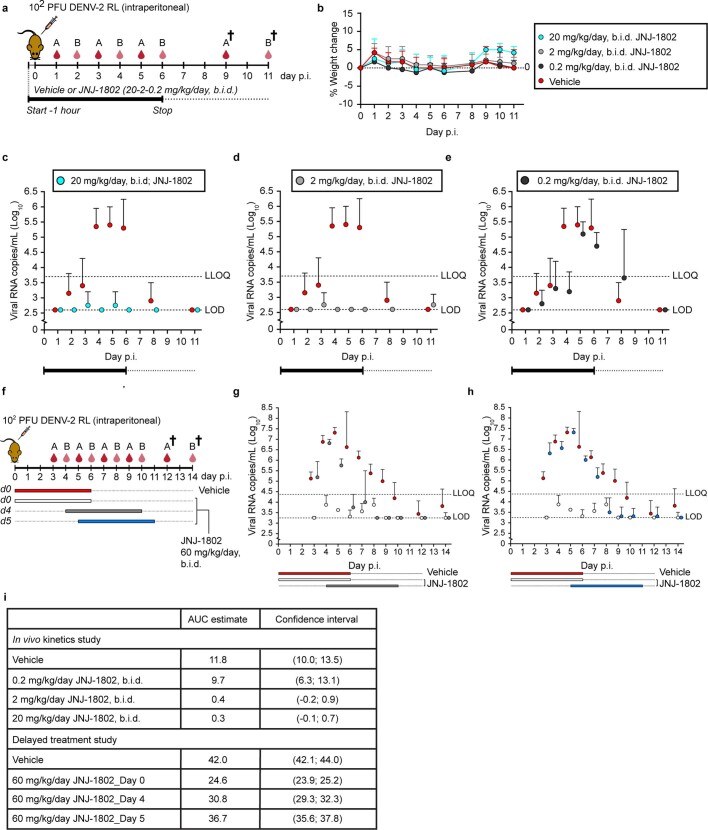
*In vivo* efficacy of JNJ-1802 against DENV-2 infection, dosed twice daily for 6 days. **a**, Schematic of the *in vivo* kinetics study. Compound administration started 1 h before infection and continued twice daily (b.i.d.) for 6 consecutive days. Each treatment group (n = 8) was equally divided into sub-groups A and B for blood collection on alternating days. **b**, Weight curves (mean values ± s.d.) of AG129 mice in the different treatment groups. **c–e**, Inhibitory effect of JNJ-1802 on viraemia in mice treated b.i.d. with 20, 2 or 0.2 mg/kg/day JNJ-1802 as compared to vehicle-treated mice. Data (mean ± s.d.) are from a single experiment. **f**, Schematic of the *in vivo* kinetics study in a therapeutic setting with treatment starting on Day 4 or 5 after DENV-2 challenge. In the control groups (vehicle and JNJ-1802) treatment started on the day of infection. Each treatment group (*n* = 10) was equally divided into sub-groups A and B for blood collection on alternating days. **g, h**, Inhibitory effect of JNJ-1802 on viraemia in AG129 mice with treatment (60 mg/kg/day, b.i.d. for 6 consecutive days) starting on Day 4 (**g**) and Day 5 (**h**) p.i. compared with the two control groups, vehicle and JNJ-1802. Data (mean ± s.d.) are from a single experiment. For both experiments (**c–e; g, h**), undetermined Ct values were imputed at a value of 40 (=limit of detection [LOD]), corresponding to 2.6 log_10_ viral RNA copies/mL (**c-e**) or 3.3 log_10_ viral RNA copies/mL (**g-h**). **i**, Mean AUC and 95% CIs were determined for each group depicted in (**c–e; g, h)**. If CIs did not overlap, groups were considered to differ significantly. LLOQ, lower limit of quantification, is calculated to be 3.7 log_10_ viral RNA copies/mL (**c-e**) or 4.4 log_10_ viral RNA copies/mL (**g-h**). Schemes are adapted from Kaptein et al.^3^. Source Data

Next, the effect of JNJ-1802 (20, 2 or 0.2 mg per kg per day, b.i.d., for 6 days) was evaluated on the kinetics of DENV-2 replication in AG129 mice after a non-lethal (10^2^ PFU) viral challenge in an 11 day study (setting 2) (Extended Data Fig. 4a) as previously described^3^. Administration of JNJ-1802 did not affect body weight (Extended Data Fig. 4b). We observed that the mean viral load was reduced to undetectable levels compared with the vehicle on 6 out of 8 days for the two highest doses (Extended Data Fig. 4c,d). No significant reduction in viral load was observed for the lowest dose (Extended Data Fig. 4e).

In the 3 day viraemia mouse model with high viral inoculum (setting 1), we observed significantly lower viral RNA levels (*P* ≤ 0.0001) in mice that were treated with JNJ-1802 for dosing groups 0.6–20 mg per kg per day, b.i.d., compared with the vehicle-treated mice. This reduction in viral RNA levels also translated to a significantly higher survival (≥80%; *P* ≤ 0.0021) of mice treated with JNJ-1802 for dosing groups 0.6–20 mg per kg per day, b.i.d., compared with the vehicle-treated mice (0% survival) (setting 3, lethal DENV challenge model; Fig. 2c,d). In this model, an anti-flavivirus antibody, clone 4G2, was administered to the mice one day before DENV infection to induce ADE and therefore accelerates virus-induced disease.

Lastly, the effect of JNJ-1802 (60 mg per kg per day, b.i.d., 6 consecutive days) was assessed in a therapeutic setting (setting 4) during a non-lethal (10^2^ PFU) DENV-2 infection in AG129 mice (Extended Data Fig. 4f). When JNJ-1802 treatment was initiated on day 4 after infection, a time at which substantial viraemia in the controls was observed, a significant reduction in the DENV-2 RNA level (mean AUC of 30.8 copies × days per ml) was observed compared with the vehicle group (mean AUC of 42.0 copies × days per ml), in which viral loads returned to levels below the lower limit of quantification (LLOQ) within 48 h (Extended Data Fig. 4g,i). Even when treatment was first initiated on day 5 after infection, the day at which replication reached its peak, a significant antiviral effect (with confidence intervals based on AUC estimates) was observed on the mean RNA AUC (36.7 copies × days per ml) compared with the vehicle control (42.0 copies × days per ml) (Extended Data Fig. 4h,i).

Next, we investigated the efficacy of JNJ-1802 treatment initiated before virus challenge on dengue disease caused by the other three DENV serotypes (DENV-1, DENV-3 and DENV-4) in AG129 mice (Fig. 2e). In the vehicle-treated mice challenged with either one of the three serotypes, survival was low—20% for DENV-1-challenged mice and 0% for DENV-3- and DENV-4-challenged mice. By contrast, 100% of the DENV-1-, DENV-3- or DENV-4-infected mice treated b.i.d. with JNJ-1802 at 60 or 6 mg per kg per day survived the study and remained disease-free until the end of the study (day 6) (Fig. 2f–h). Furthermore, the viral RNA load on day 3 after infection was significantly reduced in the JNJ-1802-treated mice that were infected with any of the three serotypes compared with the vehicle-treated controls (Fig. 2i–k).

Taken together, these results extend the in vitro data by demonstrating that JNJ-1802 is highly active against all four DENV serotypes in mice.

## Efficacy against DENV-2 in NHPs

**Fig. 3:**
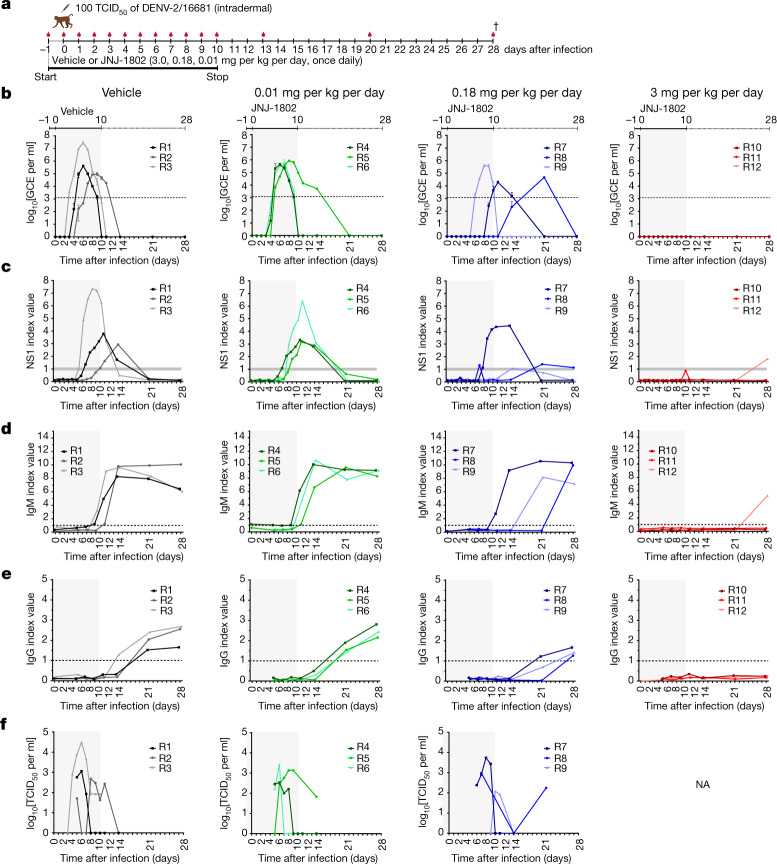
In vivo efficacy of JNJ-1802 against DENV-2 in NHPs by measuring viral RNA load, NS1 protein, IgM/IgG response and TCID_50_. **a**, Schematic of the viraemia studies using rhesus macaques. **b**, The effect of JNJ-1802 on viral RNA in plasma of rhesus macaques (R1–R12) treated q.d. with vehicle, or 0.01, 0.18 or 3 mg per kg per day JNJ-1802 (*n* = 3 per group). Treatment started 1 day before infection. Analyses were performed in triplicate. Data are mean ± s.d. The LLOQ or LOD of 1,286 GCE per ml is indicated by a dotted line in the graphs. Undetectable levels are shown as 10^0^ GCE per ml. **c**, The levels of NS1 protein in serum samples of rhesus macaques. Analyses were performed in duplicate. Data are mean. An index value of less than 0.9 is considered to be negative, between 0.9 and 1.1 equivocal, and all values greater than 1.1 positive. The area between 0.9 and 1.1 is indicated by a grey horizontal bar. **d**,**e**, The levels of IgM (**d**) or IgG (**e**) antibodies in the sera of groups of rhesus macaques. IgM/IgG antibody levels are expressed as the mean index value from two independent assays. An index value of greater than 1.0 is presumptive for the presence of IgM/IgG antibodies to DENV. The cut-off value of 1.0 is indicated by a dotted line in the graphs. **f**, Quantification of infectious virus in plasma samples of rhesus macaques using the 50% tissue culture infective dose (TCID_50_) assay. Only samples that tested positive by RT–qPCR assay or in the NS1 ELISA, as well as samples with indeterminate outcomes in these assays, were analysed. NA, not applicable. For **a**, the schematic was adapted from ref. ^3^, Springer Nature Limited. The dagger symbol indicates the day of euthanasia. Source Data

To investigate whether the potent antiviral effect of JNJ-1802 observed in mice translates to species that are more closely related to humans and naturally susceptible to DENV infection, the effect of JNJ-1802 was studied in NHPs challenged with DENV-2 (Fig. 3a).

**Extended Data Fig. 5:**
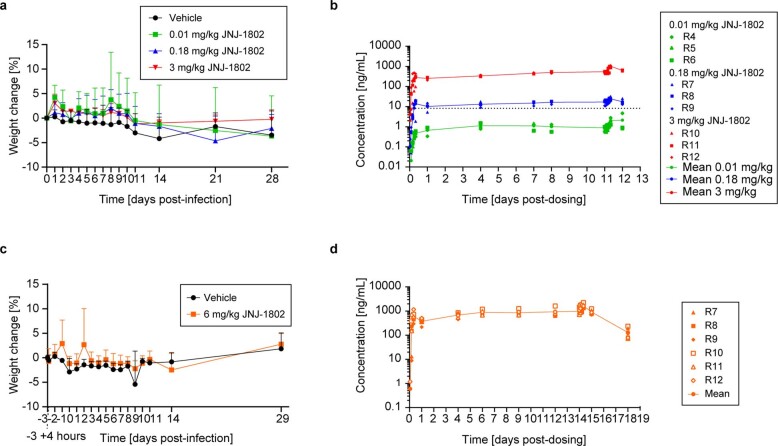
Individual and mean JNJ-1802 plasma concentration-time profiles in non-human primates. **a**, Weight curves (mean values + standard deviation) of rhesus macaques in the different treatment groups (*n* = 3 per group, except for Day 3, n = 1 for 0.01 mg/kg/day and 0.18 mg/kg/day groups and n = 2 for 3 mg/kg/day group). **b**, Mean JNJ-1802 plasma concentration-time profile for the DENV-2 study in NHP. Rhesus macaques (*n* = 3 per group) were treated prophylactically with different doses (3, 0.18 or 0.01 mg/kg/day, once daily) of JNJ-1802, and subsequently experimentally infected with 10^2^ TCID_50_ of DENV-2/16681. Dashed line indicates 3x protein-binding adjusted 90% effective concentration (pbaEC_90_) against DENV-2/16681 (8.2 ng/mL). **c**, Weight curves (mean values + standard deviation) of rhesus macaques in the different treatment groups (*n* = 6 per group). **d**, Mean JNJ-1802 plasma concentration-time profile for the DENV-1 study in NHP. Rhesus macaques were prophylactically treated with JNJ-1802 at 6 mg/kg/day once daily (*n* = 6, except for Day 1 n = 2) and subsequently infected with DENV 1/45AZ5 (0.5 mL at 1.2x10^5^ plaque forming units [PFU]/mL). NHP, non-human primates. Source Data

As seen previously in mice, JNJ-1802 showed a favourable pharmacokinetic profile in rhesus macaques (*Macaca mulatta*) with a low clearance and moderate volume of distribution translating to a terminal half-life of 50 h (Extended Data Table 2a). The plasma protein binding in rhesus macaques was higher than 99.9%. The bioavailability in monkeys ranged from 18–27% (Extended Data Table 2a). Both compound-treated and vehicle-treated monkeys infected with DENV-2/16681 exhibited a comparable weight loss over the course of the study, which was not attributable to treatment with JNJ-1802 (Extended Data Fig. 5a). Moreover, the results of the haematological analysis did not show any indication for a treatment-related effect (Supplementary Tables 4–7). No adverse effects were observed in these animals. Viral RNA (≥LLOQ) was first detected in all vehicle-treated control animals between day 3 and 7 after infection. By contrast, animals in the highest-dose group (3 mg per kg per day) had undetectable DENV RNA levels throughout the study period (up to day 28) (Fig. 3b). At the intermediate dose of 0.18 mg per kg, one out of three animals became DENV-RNA positive (≥LLOQ) on day 6 after infection, the second animal on the last day of dosing (day 10 after infection) and the last animal on day 21 after infection (Fig. 3b). Animals in the lowest-dose group (0.01 mg per kg per day JNJ-1802) became DENV-RNA positive (≥LLOQ) on day 5 after infection (Fig. 3b). When a Bayesian nonlinear dose–response model was fitted to these data, peak viraemia in the lowest-dose group (0.01 mg per kg per day) was comparable to the vehicle controls. However, in the median-dose group (0.18 mg per kg per day), peak DENV RNA levels were significantly reduced by 1.14 log_10_ genome copy equivalents (GCE) per ml plasma (95% confidence interval (CI) = −1.99 to −0.37) compared with the vehicle controls (95% CI = 5.47–5.85) as estimated using the Bayesian non-linear dose–response model and, in the high-dose group, a reduction by 4.57 log_10_ GCE per ml plasma (95% CI = −6.27 to −3.49) was observed. The detection of the viral NS1 protein in the serum aligns with the results of the quantitative PCR with reverse transcription (RT–qPCR) analysis, although NS1 was detected 2–4 days after the detection of the DENV RNA, with the exception of one animal in the highest-dose group, for which no RNA was detected, but still a positive NS1 signal was observed on day 28 (Fig. 3c). DENV immunoglobulin G (IgG) and IgM seroconversion occurred in all of the animals in the vehicle-treated group and the lowest-dose group (0.01 mg per kg per day) (Fig. 3d,e). For two out of three animals that received the intermediate dose of 0.18 mg per kg per day, IgM seroconversion occurred at later timepoints compared with the vehicle-treated group and the lowest-dose group, whereas IgG seroconversion was observed at later timepoints in all three animals. At the highest dose tested (3 mg per kg per day), one out of three animals had detectable anti-DENV IgM antibodies on day 28, consistent with the detection of NS1 protein but without a measurable RNA signal, and none of the animals had detectable anti-DENV IgG antibodies throughout the study period (Fig. 3d,e). The titre of infectious DENV particles in the plasma was quantified in samples that tested positive in the RT–qPCR assay or in an NS1 enzyme-linked immunosorbent assay (ELISA; Fig. 3f). Plasma samples with detectable DENV RNA levels also had detectable infectious virus levels at various timepoints after infection.

Plasma profiles of JNJ-1802 in DENV-2-infected rhesus macaques are shown in Extended Data Fig. 5b. After the first and the last oral administration with 0.01, 0.18 and 3 mg per kg of JNJ-1802, absorption was rather slow with time to reach maximum plasma concentrations (*t*_max_) up to 24 h. Moreover, *C*_max_ and AUC_0–24h_ values increased more than dose proportionally. After repeated administration with 0.01, 0.18 and 3 mg per kg, *C*_max_ and AUC_0–24h_ values were higher compared with a single dose, with ratios of 4, 1.7 and 2.9 for *C*_max_ and 4, 2.2 and 3.4 for AUC_0–24h_, respectively, consistent with a long terminal half-life (50 h) (Extended Data Table 2c). The predicted efficacious concentration of JNJ-1802 against DENV-2/16681, estimated as 3 times the protein-binding adjusted 90% effective concentration (3× pbaEC_90_) value of JNJ-1802 against DENV-2/16681, was 14 nM (or 8.2 ng ml^−1^) (Table 1). A similar approach has been used previously to predict the efficacious concentration of compounds against other viruses^25–28^. For the highest-dose group (3 mg per kg per day), with undetectable DENV RNA levels throughout the 28 day study period, the exposure values were well above (40–230-fold) 8.2 ng ml^−1^ at 24 h after the first dose. For a dose of 0.18 mg per kg per day, exposure values were just above 8.2 ng ml^−1^, translating to an intermediate effect on RNA and NS1 as described above. Only the lowest dose of 0.01 mg per kg per day resulted in exposure levels below the 3× pbaEC_90_ value, which may explain why this dose lacked efficacy.

## Efficacy against DENV-1 in NHPs

**Fig. 4:**
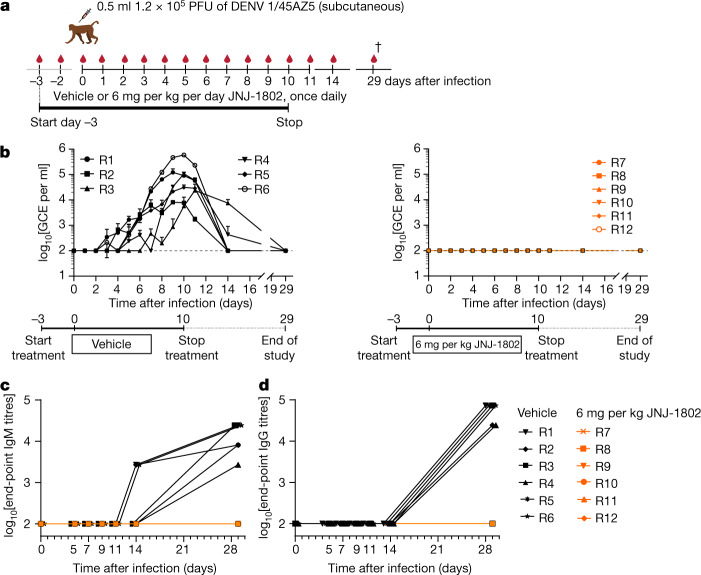
The in vivo efficacy of JNJ-1802 against DENV-1/45AZ5 was examined in NHPs by measuring viral RNA load and IgM/IgG response. **a**, Schematic of the DENV-1/45AZ5 study. **b**, The effect of JNJ-1802 on viral RNA in rhesus macaques (R1–R12) infected with DENV-1/45AZ5 (0.5 ml at 1.2 × 10^5^ PFU per ml) and treated with JNJ-1802 at 6 mg per kg per day once daily (*n* = 6; right) in comparison to the vehicle-treated group (*n* = 6; left). Treatment started 3 days before infection. RNA levels are expressed in GCE per ml. Data are mean ± s.d. The LLOQ of 100 GCE per ml is indicated by a dotted line in the graphs. **c**,**d**, ELISA data are presented as end-point titres by study day, which is defined as the reciprocal of the highest dilution of the serum that gives a positive signal. Samples were assayed in duplicate. Data are mean. For **a**, the schematic was adapted from ref. ^3^, Springer Nature Limited. The dagger symbol indicates the day of euthanasia. Source Data

To assess whether the findings in the DENV-2/16681 rhesus macaque model also applied to other DENV serotypes, the antiviral effect of JNJ-1802 was evaluated in a prophylactic setting in rhesus macaques infected with DENV-1/45AZ5 (Fig. 4a). On the basis of an assessment of weight loss (treatment with JNJ-1802 did not affect body weight; Extended Data Fig. 5c) and body temperature, no adverse effects were observed in these animals. All of the vehicle-treated animals presented with viraemia. The viraemic window varied by animal, with the earliest positive signal detected on day 3 and the latest on day 14 (Fig. 4b (left)). By contrast, DENV RNA remained undetectable during the complete study period in JNJ-1802-treated animals (Fig. 4b (right)), indicating that JNJ-1802 is highly effective in preventing DENV-1 infection in this NHP model. Humoral immune responses to the DENV-1 challenge were observed for IgM and IgG for vehicle-treated animals, while they were undetectable for JNJ-1802-treated groups (Fig. 4c,d). Plasma profiles of JNJ-1802 are shown in Extended Data Fig. 5d. After the first and the last oral administration, absorption was slow. After repeated dosing (14 doses), *C*_max_ and AUC_0–24h_ values were higher compared with single dosing, with ratios of 3.6 for *C*_max_ and 4.0 for AUC_0–24h_, consistent with a long terminal half-life (50 h) (Extended Data Table 2c).

## Discussion

Dengue remains a major global health concern for which effective therapeutic and prophylactic treatment options are urgently needed. Currently, effective direct acting antiviral drugs against dengue are lacking, and the development of vaccines poses significant challenges^29^.

The four serotypes of DENV (DENV-1–4) are genetically diverse and share only around 60–75% homology at the amino acid level^30^, while viruses within the same serotype share more than 97% homology^30^. Maintaining efficacy against all four serotypes is essential for a dengue antiviral drug to overcome ADE but has proven challenging owing to limited homology over the different serotypes as exemplified by the discontinuation of several NS4B-targeting antiviral compounds lacking pan-serotype activity (for example, compound 14a^31^, NITD-618 (ref. ^32^) and JNJ-1A^33^). Moreover, one of the biggest hurdles of vaccine development is the requirement of a tetravalent balanced immune response against these four serotypes^34^.

Here we present a direct-acting small-molecule anti-dengue compound, JNJ-1802, that exhibits picomolar to nanomolar in vitro activity against a panel of clinical and lab-adapted DENV strains, representative of the different genotypes within each DENV serotype. JNJ-1802 is also highly effective in reducing viral RNA load and virus-induced disease in DENV infection mouse models for each of the four DENV serotypes. Given its excellent in vitro and in vivo DENV pan-serotype coverage, combined with the low frequency of resistance mutations occurring naturally as polymorphisms, JNJ-1802 is now progressing into clinical studies to evaluate its efficacy against the diverse sequence spectrum comprising all four DENV serotypes.

We previously reported on JNJ-A07—a compound from the same chemical series as JNJ-1802. As JNJ-1802 has an improved preclinical safety profile compared with JNJ-A07^3^, JNJ-1802 was selected over JNJ-A07 as a clinical candidate. Mechanism of action studies revealed that JNJ-1802 prevents the interaction between NS3 and NS4B, without the ability to disrupt already established NS3–NS4B complexes. Whether this is caused by the binding of the compound to NS4B and consequently blocking the association with NS3, or by more indirect effects, such as altered cleavage of the precursor affecting NS3–NS4B interaction, remains to be determined.

Most of the mouse models in which JNJ-1802 was studied and showed promising efficacy were based on prophylactic dosing whereby treatment was initiated before virus challenge. Importantly, we also demonstrate that JNJ-1802 is effective in a therapeutic setting in mice. When treatment was initiated as late as day 4 or day 5 after infection, at the time of peak viraemia, the DENV RNA in mice was still efficiently reduced. Thus, JNJ-1802 has the potential to be effective in both prophylactic and therapeutic settings. Prophylaxis can be beneficial for travellers to, as well as for vulnerable populations living in, dengue-endemic regions^21^, as shown by the effective use of prophylactic drugs for the prevention of malaria^35,36^. A dengue antiviral drug administered as pre-exposure prophylaxis may have an effect across different levels, that is, individual and community. At the individual level, prophylactic treatment may decrease or ideally prevent viraemia, which may consequently reduce or prevent progression to clinical disease^3,21^. At the community level, it might exert its effect through the prevention or reduction of DENV transmission from human to mosquito and, therefore, potentially stop dengue outbreaks^37^. Nevertheless, the long-term use of a prophylactic agent may facilitate the development of resistant variants when residual virus is still propagating. To minimize the risk of resistance, the antiviral should efficiently suppress viral replication during prophylactic use, as was shown for JNJ-1802 in several mouse and NHP models. In case residual replication would still occur during prophylactic use of an efficacious antiviral, pathogen populations within the host might be kept small making it unlikely that resistance would arise^3,38^. Moreover, as shown by ref. ^3^, the JNJ-A07-resistant virus retained full replication competence in Vero E6 cells, while it hardly replicated in C6/36 mosquito cells. This suggests that, even if such mutations would still develop, the mosquito–human transmission cycle may be interrupted^3^. Some of the main challenges for dengue therapeutics are the rapid decline in patient viraemia during the febrile phase and the hesitancy of patients to seek medical attention early on. As viraemia lasts for only around 1 week in the blood of an individual with DENV infection before it gets resolved by the immune system^39,40^, the treatment window is limited. Thus, early, rapid and sensitive diagnostics are indispensable to identify and treat patients early on during the critical treatment window so that the antiviral can exert its maximal effect^41^.

The pathogenesis of severe dengue disease is not well understood, but it is mostly described to be attributed to ADE due to its association between higher viraemia and protein antigenaemia in patients with secondary infection^42,43^. Viraemia is 10- to 100-fold higher in severe dengue (dengue haemorrhagic fever and dengue shock syndrome) compared with mild cases of dengue disease^42,44^. The number of target cells infected, and the resulting viraemia titre, could determine the ratio of proinflammatory cytokines and therefore the intensity of the inflammatory effects in the haemostatic system, which may be reflected in a worse clinical outcome. Furthermore, the longer duration of viraemia after onset of symptoms^45^ may potentially allow for a more attainable window for the administration of the antiviral. Ongoing clinical trials will provide further understanding about the effect of JNJ-1802 on severity of a DENV infection and the effect on disease outcomes. One of the aims of the treatment trials is to understand whether a decrease in viral load by our dengue inhibitor will translate to a faster resolution of clinical symptoms.

Resistance mutations for JNJ-1802 were identified within NS4B, with the first persistent mutation (V91A) emerging after 20 passages. Two additional mutations, which conferred a high level of resistance, were observed only after 35 or more passages (approximately half a year of in vitro culturing) of increased drug pressure. This is in stark contrast to, for example, hepatitis C virus (another member within the family of *Flaviviridae*) protease inhibitors and non-nucleoside RNA-dependent RNA-polymerase inhibitors, which develop mutations after ten cell passages or less, and are therefore both associated with a low genetic barrier to resistance^46,47^. By contrast, the HIV drug tenofovir is described as having a high genetic barrier to resistance, as it takes weeks/months for resistance to develop^48^. Similarly, we consider that JNJ-1802 has a high genetic barrier to resistance. Importantly, the individual drug-resistance mutations are not, or only at a very low percentage (≤0.52%), naturally occurring in the Virus Pathogen Resource (www.viprbrc.org) database, while the combination of three JNJ-1802 resistance mutations together into one viral genome did not exist in the database. This suggests that such highly resistant mutants most likely do not occur naturally in the population. Considering that viraemia typically lasts for about 1 week, resulting in a short treatment period^39,40^, it can be assumed that such a short time frame may not allow for the selection of drug-resistant variants.

To further examine the potential of JNJ-1802, its prophylactic effect was studied in NHP models of DENV infection. In contrast to WT mice (which are immunocompetent in contrast to interferon (IFN)-signalling-deficient AG129 mice), rhesus macaques are susceptible to DENV infection, have an intact innate immune response resembling that of humans and develop detectable viraemia, although they do not exhibit clear clinical signs of infection^49^. JNJ-1802 showed excellent efficacy against DENV-1 and DENV-2 infection in NHPs, an important milestone when considering the clinical challenge of developing antiviral drugs against DENV in humans. No viral RNA was detected in the animals of the highest dose group after DENV-1 or DENV-2 infection. At the intermediate dose of 0.18 mg per kg, the animals became DENV-RNA positive (≥LLOQ) on day 6, day 10 or day 21 after infection. After stopping of drug treatment at day 10 after infection, rebound of the virus was observed in two animals (animals R7 and R8). As shown by the detection of DENV-RNA below LLOQ, the replication was only partially blocked with this intermediate dose, indicating that the virus could enhance its replication after stopping of treatment and before protective immunity emerged. At a high dose of 3 mg per kg, rebound of virus was not observed. The exposure achieved was probably sufficient to efficaciously block viral replication or at least to keep it at a very low level during the duration of the study. Thus, sufficient drug exposure of the compound will be critical. Virological and immunological characterization of any potential viral or symptomatic rebound will be further investigated in clinical studies. Nevertheless, in most of the animals treated with the highest dose of JNJ-1802, DENV IgG and IgM seroconversion was not observed during the study period of 28 days, and in the one animal in which IgM seroconversion did occur, it was delayed compared with the controls. The ability of a prophylactic dosing strategy to prevent seroconversion needs to be evaluated in future clinical studies.

JNJ-1802 has successfully completed a phase I first-in-human clinical study in healthy volunteers, showing that the antiviral is safe and well tolerated in humans^4^. The activity of JNJ-1802 both for the prevention and for treatment of DENV is currently being evaluated in further clinical studies. The preclinical data described in this manuscript, including the data in NHPs, together with the phase I first-in-human healthy volunteer data, have guided the dose-selection for JNJ-1802 against DENV infection for the clinical trials, demonstrating the value of this work. A highly potent and safe pan-serotype DENV inhibitor with a high barrier to resistance may potentially have an important role in an integrated approach to control dengue.

## Methods

### Compound

The synthesis of JNJ-1802 (Fig. 1a) is published in patent application WO 2016/180696. The synthesis and chemical characterization of JNJ-1802 are also provided in the Supplementary Methods. For in vitro experiments, the compound was dissolved in 100% dimethyl sulfoxide (DMSO) as a 10  mg ml^−1^ or a 5 mM stock. For oral administration in AG129 mice, JNJ-1802 was dissolved in 80% polyethylene glycol 400 (PEG400)/20% H_2_O. For oral administration in rhesus macaques (*M. mulatta*), JNJ-1802 was dissolved in 100% PEG400. The formulations used during the study in NHPs were prepared a maximum 3 days before dosing and stored at room temperature, protected from daylight.

### Cells

Vero cells (African green monkey kidney cells; European Collection of Authenticated Cell Cultures (ECACC), CL 84113001/American Type Culture Collection (ATCC) CCL-81) were maintained in Eagle’s minimum essential medium (MEM) supplemented with 10% fetal bovine serum (FBS) (Sigma-Aldrich), 2 mM l-glutamine and 0.02 mg ml^−1^ gentamicin (Thermo Fisher Scientific). Vero E6 cells (ATCC, CRL-1586) were cultured in MEM supplemented with 10% heat-inactivated FBS, 2 mM l-glutamine and 100 U ml^−1^ penicillin–streptomycin (Sigma-Aldrich). Huh-7 hepatoma-derived cells were maintained in Dulbecco’s modified Eagle’s medium (DMEM), supplemented with 10% FBS, 2 mM l-glutamine and 0.02 mg ml^−1^ gentamicin. In antiviral assays using Vero and Huh-7 cells, the culture medium contained 2% FBS and 10% FBS, respectively. In antiviral experiments with Vero E6 cells, 2% heat-inactivated FBS was used. Huh-7 replicon cells were cultured in the same medium as mentioned above, supplemented with 75 µg ml^−1^ hygromycin B (Roche).

Huh-7 cells stably expressing T7 RNA polymerase and DENV NS2B-NS3 (Huh-7–T7/NS2B–NS3 cells) were generated by lentiviral transduction, as previously described^52^. Cells were cultured at 37 °C and 5% CO_2_ in DMEM, supplemented with 10% FBS, 2 mM l-glutamine, 100 U ml^−1^ penicillin, 100 µg ml^−1^ streptomycin, 5 µg ml^−1^ zeocin, 1 µg ml^−1^ puromycin and 1% non-essential amino acids.

THP-1 cells (TIB-202, ATCC) expressing dendritic-cell-specific intercellular adhesion molecule-3-grabbing non-integrin (DC-SIGN) were propagated in Roswell Park Memorial Institute (RPMI) (Lonza) supplemented with 10% heat-inactivated FBS (F7524, Sigma-Aldrich) and 0.04% gentamicin (Gibco-Life Technologies). All cell lines (Vero, Huh-7 and THP-1/DC-SIGN) were regularly tested for mycoplasma contamination.

C6/36 mosquito cells (from *Aedes albopictus*; ATCC, CCL-1660) were cultivated in the absence of 5% CO_2_ at 28 °C in Leibovitz’s L-15 medium (Thermo Fisher Scientific), supplemented with 10% FBS, 1% non-essential amino acids (Thermo Fisher Scientific), 1% HEPES buffer (Thermo Fisher Scientific) and 1% penicillin (100 U ml^−1^) and streptomycin (100 μg ml^−1^) solution (Thermo Fisher Scientific).

### Antibodies

Glyceraldehyde-3-phosphate dehydrogenase (GAPDH) or β-actin were used as loading controls for cell lysates (input), and were visualized using mouse monoclonal anti-GAPDH, G-9 (sc-365062, I2320, Santa Cruz Biotechnology, 1:1,000) or mouse monoclonal anti-β-actin, AC-15 (A5441, 079M4799V, Sigma-Aldrich, 1:5,000) antibodies. For immunoprecipitation experiments, mouse monoclonal anti-HA agarose beads (HA-7, A2095, 119M4756V, Sigma-Aldrich), were used and NS4B–HA was detected using a purified mouse anti-HA.11 epitope tag (16B12, 901502, B276381, BioLegend, 1:1,000). NS4B- and NS3-specific bands were visualized using custom-generated rabbit polyclonal antibodies directed against NS3 (1:2,000) or NS4B (1:1,000)^50^. Anti-flavivirus group monoclonal antigen antibodies (D1-4G2-4-15, MAB10216, 2441960, Millipore/Merck) were used in the survival study in AG129 mice (injected with 100 µl, 1:50) before DENV infection to induce ADE and therefore accelerate death and in the 50% tissue culture infective dose (TCID_50_) assay, in which virus production was determined using custom-developed colorimetric detection of viral envelope protein using the anti-flavivirus group antigen monoclonal antibody as primary antibody. The 4G2 mouse monoclonal anti-DENV capture antibody was also used to coat 96-well plastic flat-bottom plates for the IgM and IgG ELISA. Secondary antibodies used in this assay were peroxidase-labelled goat anti-monkey lgM (KPL, 074-11-031) or lgG (Sigma-Aldrich, SAB3700766).

### Viruses

A clonal stock of the strain DENV-2/16681 was produced by transfection of in vitro transcribed RNA of plasmid pFK-DVs into Huh-7 cells^3^. DENV-2/16681/eGFP, carrying enhanced green fluorescent protein (eGFP) at the amino terminus of the capsid protein, was produced through the transfection of in vitro transcribed RNA of plasmid pFK-DV-G2A into Huh-7 cells^3,53^.

A total of 20 DENV strains, representing the available genotypes within the four DENV serotypes, were used in the in vitro genotype panel, from which 19 viruses were described previously in ref. ^3^ and one additional virus, DENV-1/45AZ5 (ref. ^54^), was added.

For antiviral assays using Vero cells, in vitro resistance-selection experiments and in vivo efficacy studies in mice, the DENV-2 Rega Laboratory strain (referred to as DENV-2 RL) was used (GenBank: MW741553; provided by V. Deubel). For in vivo studies in mice, high-titre stocks were generated by propagating the virus in C6/36 mosquito cells and subsequently concentrating, either by ultracentrifugation or tangential flow filtration using tangential flow filtration capsules (Minimate TFF; Pall Life Sciences), according to the manufacturer’s protocol. Infectious virus titres (PFU per ml) were determined by performing plaque assays on baby hamster kidney cells (BHK-21, ATCC (CCL-10)).

Four non-dengue flaviviruses were used: ZIKV (H/PF/2013, French Polynesia, GenBank: KJ776791), JEV (CNS769-Laos 2009, Laos, GenBank: KC196115), WNV (R94224, CDC human brain 29 September 2008, Wisconsin, GenBank: MF004388) and yellow fever virus (YFV; 88-99, Bolivia, GenBank: MF004382). All other viruses, not belonging to the *Flavivirus* genus, are detailed in the Supplementary Methods and Supplementary Table 2.

To test the efficacy against the DENV serotypes in the AG129 mouse model (129/Sv mice deficient in both IFNα/β and IFNγ receptors) with mortality read-out, DENV-1/West Pac (genotype IV), DENV-3/C0360/94 (genotype II) or DENV-4/703/4 (genotype II) was used. The virus strains and culture procedure to generate the virus stocks have been described previously^55^. For proof-of-concept studies in rhesus macaques, the virus strains DENV-1/45AZ5 (ref. ^54^) and DENV-2/16681 were used. DENV-2/16681 stocks were generated by propagating the virus in C6/36 mosquito cells. Infectious virus titres were determined on Vero cells (ATCC, CCL-81) using standard procedures. DENV-1/45AZ5 was grown in fetal rhesus lung cells and the virus was not further concentrated^54^.

### Antiviral assays

The antiviral activity of JNJ-1802 against DENV-2/16681/eGFP was determined in a phenotypic antiviral assay with eGFP readout as a measure for viral replication. In parallel, the toxicity was measured using an ATPLite cell viability luminescence assay (PerkinElmer). The assays were performed in three different cell types (Vero, Huh-7 and THP-1/DC-SIGN) as described in ref. ^3^ to exclude cell-specific activity of the compound.

The antiviral activity of JNJ-1802 against the DENV-2 RL strain was determined in Vero and C6/36 cells by measuring the levels of viral RNA using RT–qPCR as previously described^3^. A potential toxic effect on host cells was tested in parallel, while omitting virus infection, using the MTS/PMS method (Promega) for Vero cells, or by high-content imaging for C6/36 cells, as described previously^3^. Antiviral activity of JNJ-1802 against the DENV genotype panel (Supplementary Table 1) was assessed by measuring viral RNA using RT–qPCR as described previously^3,23^. RT–qPCR data were analysed using the QuantStudio 12K Flex software (v.1.2.3) or SDS v.1.2 Applied Biosystems software. Inhibition values for antiviral molecules were plotted using KaleidaGraph plotting software (v.4.03, Synergy Software). The antiviral activity of JNJ-1802 against DENV-1/45AZ5 was tested in Vero cells by measuring viral RNA using RT–qPCR as described previously^56^. Antiviral activity/toxicity assays of JNJ-1802 for all other viruses, not belonging to the flaviviruses, were performed using either reporter systems, cell viability, RT–qPCR or plaque reduction assays as readout, as further specified in the Supplementary Information and Supplementary Table 2.

DENV-2 in vitro resistance selection experiments were performed in Vero cells with gradually increasing concentrations of JNJ-1802 (ref. ^3^). Whole-genome sequencing was performed in-house on DENV variants collected at every fifth passage and at the end of the experiments (that is, passage 42 for experiment A and passage 50 for experiment B) as described previously^3^.

### Transient mutant replicon assays

A panel of WT and mutant subgenomic DENV reporter replicons (sgDVs-R2A) was used to determine the compound resistance imposed by each of these mutations^3^.

### Immunoprecipitation experiments

HA-immunoprecipitation experiments were performed as previously described^3^ with minor modifications. In brief, for dose–response assays, cells were transfected for 4 h, followed by addition of fresh DMEM supplemented with JNJ-1802 or an equivalent amount of DMSO and cells were collected 18 h after treatment. For the time-of-addition experiments, cells were drug treated either 4 h or 24 h after transfection and, in either case, collected 8 h after treatment. Immunoprecipitation was conducted using mouse anti-HA monoclonal agarose beads (Sigma-Aldrich). Cell lysates and captured protein complexes were analysed by western blotting using a home-made polyclonal antibody for the detection of NS3 and both a home-made polyclonal anti-NS4B-specific antibody and a monoclonal anti-HA-specific antibody for detecting NS4B-containing protein species (or HA-tagged modifications thereof). Intensities of NS3- and NS4B–HA-specific bands (the latter determined by the anti-HA-specific antibody) were quantified using ImageJ (v.2.1.0/1.53j; Wayne Rasband and contributors, National Institutes of Health, USA). To ensure equal weighting of individual experiments, signal intensities of NS3 and NS4B–HA species (NS4A-2K-NS4B–HA, 2K-NS4B–HA and NS4B–HA) were first divided by the corresponding sum across all samples (both in lysates and after pull-down). Ratios of NS3 to HA-tagged NS4B-containing species were then formed and normalized to the mean ratio of the DMSO-treated control. For dose–response assays, statistical analysis was performed using repeated-measures one-way ANOVA with subsequent Dunnett’s multiple-comparisons test. In the time-of-addition experiments, statistical analysis was performed using paired two-sided *t*-tests.

### DENV-2 infection studies in mice

Breeding couples of AG129 mice were purchased from Marshall BioResources and bred in-house. The specific pathogen-free status of the mice was regularly checked at the KU Leuven animal facility. Mice were housed in individually ventilated cages (maximum of five mice per cage, type GM500 Sealsafe Plus, Tecniplast) at 21 °C, 55% humidity under a 12 h–12 h light–dark cycle. The mice were provided with food and water ad libitum as well as with cardboard play tunnels and cotton as extra bedding material. Allocation to experimental groups was performed randomly. Housing conditions and experimental procedures were approved by the ethics committee of KU Leuven (licence P169/2011 and P047/2017) following institutional guidelines approved by the Federation of European Laboratory Animal Science Associations.

The efficacy of JNJ-1802 on viraemia, viral kinetics and virus-induced disease (survival) was evaluated in DENV-2 infection models in AG129 mice (Supplementary Table 3) as described previously^3^.

In brief, in day 3 viraemia studies, DENV female mice (7–11 weeks old) were challenged intraperitoneally (i.p.) with 10^6^ PFU DENV-2 RL strain. Mice were treated b.i.d. by oral gavage for 3 consecutive days with either vehicle (PEG400:water (80:20); *n* = 24) or various doses of JNJ-1802 (60, 20, 6, 2, 0.6 or 0.2 mg per kg per day; *n* = 8 (60 mg per kg per day dosing group) and *n* = 16 (all other dosing groups)), with the first administration 1 h before DENV challenge. In a separate viraemia study, in which mice were orally treated for 3 days with 30, 3 or 0.3 mg per kg per day JNJ-1802 (*n* = 8 for each group), the treatment was administered once daily. On day 3 after infection, the mice were euthanized, and blood was collected and stored at −80 °C until further use. The effect of the compound on viral RNA levels in the blood on various days after infection was monitored in an in vivo kinetics study. AG129 female mice (aged 7–11 weeks, *n* = 16 per group) were inoculated i.p. with 10^2^ PFU DENV-2 RL strain. Mice were treated b.i.d. through oral gavage with vehicle or JNJ-1802 using three different doses: 20, 2 or 0.2 mg per kg per day. In the kinetics studies, treatment was initiated 1 h before DENV infection and continued for 6 consecutive days. Each group was subdivided into two smaller groups (A and B; *n* = 8 each), from which blood was collected on alternating days: on day 1, 3 and 5 (A groups) or on day 2, 4 and 6 (B groups). On day 8 and day 11 after infection, the mice from the A and B groups, respectively, were euthanized, and blood was collected and stored at −80 °C until further use. The survival study was performed as described previously^3^ with b.i.d. dosing of AG129 mice (aged 7–11 weeks, female, *n* = 10 per group) at 20, 6, 2 or 0.6 mg per kg per day (start of treatment 1 h before DENV challenge).

In delayed-treatment studies (therapeutic setting), AG129 female mice (aged 7–11 weeks, *n* = 10 per group) were inoculated i.p. with 10^2^ PFU DENV-2 RL strain. Treatment with JNJ-1802 (60 mg per kg per day, b.i.d.) was initiated on day 4 or 5 after infection and continued for 6 days. Mice that were treated with vehicle or JNJ-1802 on the day of infection (that is, 1 h before infection) were included as controls. Each group was subdivided into two smaller groups (A and B; *n* = 5 per group), from which blood was collected on alternating days: on day 3, 5, 7, and 9 (A groups) or on day 4, 6, 8 and 10 (B groups). On day 12 and day 14 after infection, mice from the A and B groups, respectively, were euthanized, and blood was collected and stored at −80 °C until further use.

Viral RNA isolation from plasma was performed using the NucleoSpin RNA virus kit (Macherey-Nagel) according to the manufacturer’s instructions. Details of the primers, the probe and the RT–qPCR were described previously^57,58^. The LLOQ of this RT–qPCR assay was defined as 3.8 log_10_ copies per ml, which corresponds to a median cycle threshold value of 36.3.

### DENV-1, DENV-3 and DENV-4 infection in mice

Studies to evaluate the in vivo efficacy of JNJ-1802 against DENV-1, DENV-3 and DENV-4 were undertaken at the University of Texas Medical Branch (UTMB) in laboratories managed by the Animal Resources Center. The UTMB is an Association for the Assessment and Accreditation of Laboratory and Care (AAALAC) International accredited facility. All of the procedures were reviewed and approved by the UTMB Institutional Animal Care and Use Committee. The studies were carried out in strict compliance with the recommendations of the Guide for the Care and Use of Laboratory animals published by the National Research Council. Male and female AG129 mice (*n* = 10 per group; aged 6–8 weeks) were housed at a maximum of 5 mice per cage in individually ventilated cages with food and water provided ad libitum. Allocation to experimental groups was performed randomly. For the in vivo efficacy of JNJ-1802 against DENV-1, DENV-3 and DENV-4, AG129 mice (aged 6–8 weeks) were treated b.i.d. by oral gavage with JNJ-1802 (60 or 6 mg per kg per day in 0.1 ml per dose, (*n* = 10 each)) or vehicle (80% PEG400 + 20% H_2_O in 0.1 ml) (*n* = 10) for 5 consecutive days. Approximately 1 h after the first treatment, all of the animals were challenged by i.p. injection with 10^7^ PFU DENV-1/West Pac, DENV-3/C0360/94 or DENV-4/703/4. Pretreatment with anti-flavivirus antibody 4G2 to increase disease severity by induction of ADE was not required as infection with DENV-1/West Pac, DENV-3/C0360/94 or DENV-4/703/4 in our models leads to severe disease (requiring euthanasia after >20% loss of body weight). Each treatment group was subdivided into two smaller groups (groups A and B; *n* = 5 each). Blood was collected on day 3 after infection from group A to measure viral RNA; samples were collected by retro-orbital bleed on day 3 after infection around 30 min before the morning treatment. In parallel, animals (groups A and B) were followed for lethality throughout the experiment. Viral RNA isolation from plasma and RT–qPCR analysis was performed using the methods described previously^55^.

### DENV-2 study in NHPs

Pre-exposure prophylaxis to DENV-2 was assessed in 12 rhesus macaques (*M. mulatta*) at the Biomedical Primate Research Centre (BPRC), Rijswijk, the Netherlands. BPRC is an AAALAC International accredited facility, and the research protocol was approved by appropriate national authorities (CCD, Central Committee for Animal Experiments; licence number AVD5020020172884) and by the institutional Animal Welfare Body (AWB).

The animals were selected from the experimental stock from the self-sustainable BPRC colony. Before being placed on the protocol, all animals tested negative for antibodies to DENV serotypes 1, 2, 3 and 4, WNV and ZIKV. All animals were healthy, adult, male and female, Indian-origin rhesus monkeys with a minimum age of 5 years and a minimum body weight of 7 kg. During the experiment, the animals were housed in pairs with a socially compatible cage mate under biosafety level 3 (BSL3) conditions. The animals were offered a daily diet consisting of monkey food pellets, fruit and bread. Enrichment (toys, extra food) was offered daily. Drinking water was available ad libitum through an automatic watering system. The animals were randomly allocated to treatment groups according to a randomized block design on the basis of the sex and body weight of the animals. To assess the efficacy of JNJ-1802 in a prophylactic setting, rhesus macaques received daily vehicle (100% PEG400, *n* = 3), or compound JNJ-1802 at a dose of 0.01 mg per kg per day (*n* = 3), 0.18 mg per kg per day (*n* = 3) or 3 mg per kg per day (*n* = 3) dissolved in 100% PEG400 through oral gavage, starting from 1 day before experimental infection until 10 days after infection. On day 0, the animals were exposed through intradermal inoculation in the upper back with 100 TCID_50_ of strain DENV-2/16681. In the DENV-1 NHP study that was conducted first, infection with DENV-1 was performed 3 days after oral administration of JNJ-1802. As a rapid increase in drug levels was obtained 12 h after dosing and high concentration persisted up to the end of the dosing period, animals in the DENV-2 NHP study were infected already one day after the start of oral administration with JNJ-1802. The animals were monitored daily during the study period for general behaviour until day 28. Blood samples were taken at regular timepoints for both virological assessments and determination of plasma compound concentrations. Virological assessments included the quantification of DENV-2 RNA using RT–qPCR, the detection of DENV NS1 antigen by ELISA, the quantification of infectious DENV-2 by TCID_50_ assay in Vero cells and the detection of anti-DENV IgM/IgG by ELISA. Details of the assessment of haematological parameters are provided in the Supplementary Information.

### Quantification of DENV RNA using RT–qPCR

Plasma samples from ethylenediaminetetraacetic acid (EDTA)-treated blood of macaques were checked for the presence of DENV-2 RNA using RT–qPCR as described in ref. ^59^. Viral RNA was isolated from 140 µl plasma using the QIAamp Viral RNA Mini Kit (Qiagen Benelux) according to the manufacturer’s instructions. Next, 10 µl taken from the 60 µl eluate was used for cDNA synthesis and PCR amplification. This was performed using the Brilliant II QRT-PCR Core Reagent Kit, 1-Step kit (Agilent), according to the manufacturer’s instructions. The amount of viral RNA in the plasma was evaluated on a Bio-Rad CFX Connectreal-time system. A multiplication factor of 42.86 was used to calculate the amount of viral RNA per ml of plasma. The amount of viral RNA per ml plasma was determined in three independent assays. The LLOQ was 30 genomic copies per reaction.

### DENV IgM and IgG ELISA

Anti-DENV IgM and IgG were assessed in the serum of DENV-2-infected macaques using the Dengue Virus IgM Capture DxSelect ELISA test (EL1500M; Focus Diagnostics) and the Dengue Virus IgG DxSelect ELISA test (EL1500G; Focus Diagnostics) according to the manufacturer’s instructions in two independent experiments^60^. Both are qualitative assays for the detection of human IgM and IgG antibodies to DENV expressed as an index value relative to the cut-off calibrator as described in the instruction manual of the assay. To calculate index values, specimen optical density (OD) values (corrected for blank readings) were divided by the mean of the corrected cut-off calibrator absorbance values. As no specific assays were available to detect macaque antibodies in response to DENV infection, we made use of the broad cross-reactivity between the macaque antibodies and the detecting conjugates to evaluate the anti-DENV humoral immune responses in macaques.

### NS1 ELISA

Detection of NS1 antigen in serum of DENV-2 infected macaques was performed using the Alere Panbio Dengue Early ELISA (01PE40; Alere) and performed according to the manufacturers’ instructions. DENV NS1 was detected in two independent assays.

### Infectious virus quantification

Quantitative virus isolation was performed using the TCID_50_ assay. To determine the TCID_50_ per ml, plasma samples from DENV-2-infected macaques were titrated on Vero cells. In brief, the samples were 1:1 serially diluted in culture medium and added in triplicate to a monolayer of Vero cells in microtitre plates. Undiluted virus and medium alone were used as positive and negative controls, respectively. After incubation for 6 days under 5% CO_2_ and at 37 °C, the medium was discarded and the cells were fixed with cold acetone. Next, virus production was determined using a custom-developed colorimetric detection of viral envelope protein using an anti-flavivirus group antigen monoclonal antibody (D1-4G2-4-15) as the primary antibody. The plates were measured at an OD of 490 nm on the Bio-Rad iMark microplate reader. A well was scored positive when OD_sample well_ > OD_negative control_ + 0.75 × (OD_positive control_ − OD_negative control_). The 50% endpoint was calculated using the Spearman & Kärber algorithm^61,62^.

The colorimetric screening assay was validated by a direct comparison with a similar test but based on microscopic scoring of infected wells.

### DENV-1 study in NHPs

Antiviral activity of JNJ-1802 was also assessed in a prophylactic setting in rhesus macaques infected with the DENV-1/45AZ5 virus strain. The study was approved by the Walter Reed Army Institute of Research Institutional Animal Care and Use Committee (WRAIR IACUC). Research was conducted in compliance with the Animal Welfare Act and other federal statutes and regulations pertaining to animals, and the work was performed in accordance with the principles stated in the Guide for the Care and Use of Laboratory Animals, the National Research Council. WRAIR is fully accredited by AAALAC International. The animals were US-colony bred and procured from Covance Research Products. Before being placed on the protocol, all of the animals tested negative for antibodies to DENV serotypes 1–4, WNV and ZIKV by neutralization assays performed at WRAIR. The animals also tested negative for simian retroviruses, simian immunodeficiency virus and simian T cell leukaemia virus with testing performed by the vendor before shipment. The animals were single-housed under a 12 h–12 h light–dark cycle and with 10–15 room air changes per hour. The animals were fed Old World Primate Chow 5038 (Quality Lab Products) b.i.d., fresh fruit at least three times a week and water ad libitum. Environmental enrichment was provided in the form of cage complexities, food treats, opportunities to forage, a rotation of several toys and puzzles, periodic access to an activity cage that permits climbing, jumping and swinging, and alternating days of television and music. Animals were anaesthetized by intramuscular injection with a combination of ketamine (5 mg per kg) and dexmedetomidine (0.01 mg per kg) before all procedures, and anaesthesia was reversed by intramuscular injection of atipamezole (0.01 mg per kg). The studies performed under this protocol were non-terminal. Twelve healthy, adult, Indian-strain rhesus macaques, males and females, at least 5 kg in weight and aged 4–10 years were selected for the study. The animals were allocated into two experimental groups (*n* = 6 per group) in no particular order, while attempting to balance sex, ages and weights. Group 1 received 6 mg per kg per day JNJ-1802 and group 2 received vehicle (100% PEG400). Oral administration of compound JNJ-1802 started 3 days before experimental infection (day −3) and continued with daily administrations until day 10 after infection. On study day 0, which is 3 days after the start of treatment, the challenge virus (0.5 ml of DENV-1/45AZ5; 1.2 × 10^5^ PFU per ml) was administered subcutaneously into the upper arm. Whole-blood samples were collected on days −28, −3, −2, 0–11, 14 and 29 (Fig. 4a). In addition to veterinary and husbandry care, the animals were observed visually at least once daily throughout the study by research personnel.

### Quantification of DENV RNA using RT–qPCR

Serum samples from macaques were checked for the presence of DENV-1 RNA. Viral RNA for RT–qPCR was extracted from 100–200 µl of serum using the QIAcube instrumentation and the QIAGEN Viral RNA extraction kits. The primers and fluorogenic probes used in the RT–qPCR assays have been previously described^63,64^. DENV-1 RNA was detected in a 25 µl reaction containing 5 µl of extracted RNA sample, 10 pmol of dengue-specific primers and 5 pmol dengue probe in the reaction mixture from Superscript Ill Platinum One-Step Quantitative RT-PCR Kit (Invitrogen, Thermo Fisher Scientific), consisting of 0.5 µl Superscript III RT/Platinum mix, 12.5 µl 2× reaction mix and 0.05 µl ROX reference dye. The one-step RT–qPCR comprises a 30 min RT step at 50 °C and 2 min of Taq polymerase activation at 94 °C, followed by 40 cycles of PCR at 94 °C for 5 s and 53 °C for 35 s performed in an ABI 7500DX Fast Real Time PCR system. Each assay included positive, negative and non-template controls. The RNA copy number was calculated from a standard curve of tenfold serial dilutions made from RNA in vitro transcripts consisting of DENV-1 with known copy numbers. The LLOQ was 100 genomic copies per reaction.

### DENV IgM and IgG ELISA

The IgM/IgG ELISA assays were performed on sera from DENV-1 infected macaques. For IgM and IgG ELISA, 96-well plastic flat-bottom plates were coated with 4G2 mouse monoclonal anti-DENV capture antibody. After blocking, the plates were incubated with partially purified DENV-1 (WP-74 strain) virus antigen, washed and then incubated with serial dilutions of NHP serum (100-fold to 218,700-fold dilutions). After washing, the plates were incubated with the appropriate secondary antibody—either peroxidase-labelled goat anti-monkey IgM or IgG in 50% glycerol. After washing, the plates were developed using the TMB Microwell Peroxidase Substrate System (KPL, 50-76-00). Once the reaction was stopped, using an acidic solution, the enzymatic turnover of the substrate was determined by OD measurement at 450 nm. The samples were assayed in duplicate. The background was calculated as the average of all day 0 (pre-infection) sera for each dilution.

### Pharmacokinetics studies

The pharmacokinetics profile of JNJ-1802 was evaluated in a separate experiment in fed male CD-1 mice (*n* = 3 per group, 27–33 g body weight, aged around 6–8 weeks; Charles River Laboratories) at Janssen Pharmaceutica NV. The housing conditions and experimental procedures were approved by the ethics committee on animal experiments of Janssen Pharmaceutica NV (license number LA1100119). Janssen Pharmaceutica NV has full AAALAC accreditation. The mice were intravenously injected into the tail vein with 2.5 mg per kg of JNJ-1802, which was formulated as an 0.5 mg ml^−1^ solution in 70% PEG400/30% H_2_O, and blood samples were collected (in EDTA-containing microcentrifuge tubes) from the saphenous vein at 0.12, 0.33, 1, 2, 4, 7 and 24 h after dosing. Moreover, JNJ-1802 was administered by oral gavage at 1, 3, 10 and 30 mg per kg, formulated as a solution in 80% PEG400/20% H_2_O, and the blood samples were collected from the saphenous vein at 0.5, 1, 2, 4, 7 and 24 h after dosing. The blood samples were immediately centrifuged at 4 °C and the plasma was stored at −20 °C. Compound concentrations in the plasma samples were determined using the API 5500 LC–MS/MS system mass spectrometer (Applied Biosystems). Individual plasma concentration–time profiles were processed for a non-compartmental pharmacokinetics analysis using Phoenix WinNonlin v.6.3. (Certara).

The pharmacokinetics profile of JNJ-1802 was evaluated in female monkeys after single intravenous administration and in DENV-infected monkeys after repeated oral administration. After single intravenous administration, the monkey blood was collected at the BPRC just before dosing, and at 0.05, 0.15, 0.5, 4, 6, 8, 24, 48 and 264 h after dosing. After oral administration, the monkey blood was collected at the BPRC on the first and last day of dosing just before dosing and at 1, 2, 4, 6, 8 and 24 h after dosing that day. Moreover, blood samples were taken on days 3, 6 and 7 after infection just before dosing. For the study at WRAIR, the monkey blood was collected just before dosing and at 1, 4, 8 and 24 h after the first day of dosing and before dosing and at 2, 5, 8, 24 and 96 h after the last day of dosing. Furthermore, blood samples were taken on days 0, 2, 5 and 8 after infection just before dosing. Blood was drawn from the femoral vein (or a peripheral vein, for example, saphenous or cephalic) The volume of blood collected did not exceed 7.5% of an animal’s total blood volume over a 7 day period or 10% over a 2 week period. A volume of 1 ml blood was collected into a blood collection tube coated with EDTA as anti-coagulant. Within 1 h after collection, the blood samples were centrifuged in a precooled centrifuge for 10 min at 1,500*g* (4 °C). Within 2 h after blood collection, the plasma samples were stored at −80 °C until shipment. Individual plasma concentration–time profiles were processed for a non-compartmental pharmacokinetics analysis using Phoenix WinNonlin v.6.3. (Certara).

### Statistical analysis for in vivo studies

Statistical power calculations considered the number of mice required to detect a significant reduction in viraemia compared with vehicle-treated controls. With groups of *n* = 8, a reduction of at least 0.8 log_10_ in viral RNA can be detected according to the independent *t*-test (with *α* = 0.05, power = 80% and a s.d. value of 0.5). Moreover, statistical calculations considered the number of mice that were required to detect a significant improvement in survival compared with vehicle-treated controls. With groups of *n* = 11, a minimal survival rate of 60% for treated mice versus 0% in the untreated, infected control group can be demonstrated according to the Fisher’s exact test (with *α* = 0.05 and power = 80%). The experiments were not randomized, and investigators were not blinded to allocation during experiments and outcome assessment.

To assess the effect of JNJ-1802 treatment on viral load in DENV-2-infected mice for each treatment group compared with the vehicle-treated mice (viraemia studies), ordinary one-way ANOVA was used with Dunnett’s test to correct for multiple comparisons (experiment with q.d. treatment) and the pooled data of three independent studies were analysed using two-way ANOVA with Dunnett’s test to correct for multiple comparisons (experiment with b.i.d. treatment).

To assess the effect of JNJ-1802 treatment on viral load in DENV-1-, DENV-3- or DENV-4-infected mice for each treatment group compared with the vehicle-treated mice (viraemia studies), ordinary one-way ANOVA was applied with Dunnett’s test to correct for multiple comparisons (DENV-1), and Kruskal–Wallis tests with Dunn’s multiple-comparison test (DENV-3 and DENV-4). For the viral kinetics studies and the delayed-treatment studies, a batch approach was applied to calculate the viral load AUC using the PΚ R package^65^. This package estimates a mean AUC value for settings in which the animals are measured at varying timepoints within a treatment group. Within each experiment, the mean AUC value and 95% CIs were determined for each group. The AUC was calculated using the LOD (2.6 log_10_ copies per ml) as the lowest limit. In case the CI of a compound-treated group did not overlap with that of another group (vehicle or treatment), the groups were considered to be statistically different. In the delayed-treatment studies, the total viral load AUC for each of the compound-treated groups was calculated and compared with that of the vehicle-treated group. Fisher’s exact tests were used to determine whether the survival rate in DENV-2-infected mice on day 25 for each compound treatment group differed significantly from that of the vehicle group. *P* values were adjusted using the Bonferroni multiple-comparison correction method. Note that *P* values lower than 0.0001 were rounded to 0.0001. For survival studies of DENV-1-, DENV-3- or DENV-4-infected mice, Fisher’s exact tests were used on day 6. *P* values were adjusted using the Bonferroni multiple-comparison correction method.

To assess the in vivo efficacy of JNJ-1802 against DENV-2 in NHPs, no formal sample size calculation was performed. A Bayesian nonlinear inhibitory sigmoid *E*_max_ model was used to quantify the dose–response relationship between JNJ-1802 and the induced peak viral load DENV-2 in monkeys. The model estimates the mean peak viral load under placebo (dose zero) and the nonlinear decrease in function of increasing dose of JNJ-1802 under the assumptions of 100% maximum drug inhibition at infinite high dose levels. The animal caretakers who performed all animal handling and blood collections were not blinded to the DENV-2 NHP studies. However, the samples were processed by laboratory technicians who were not involved in the animal treatment/manipulation and who received the blood samples in coded tubes for further analysis.

To assess the antiviral activity of JNJ-1802 in rhesus macaques infected with the DENV-1/45AZ5 virus strain, the sample size was calculated using one-sided Fisher exact tests and an *α* = 0.05 targeting a power of at least 80%. This sample size was sufficient to detect a significant difference in the number of infected animals in each of the groups as low as 83%. Although the DENV-3 NHP studies were not formally blinded, the veterinary staff were not told which material (JNJ-1802 or placebo) they were delivering to a specific animal. Sample processing and conducting of assays was performed by laboratory technicians not involved in the treatment/manipulation of the animals. Blood samples were labelled with animal ID numbers and processed in a non-specific manner simultaneously. All assays were performed on samples of all animals by laboratory technicians; the samples and raw data were not labelled with their treatment group.

### Reporting summary

Further information on research design is available in the Nature Portfolio Reporting Summary linked to this article.

## Online content

Any methods, additional references, Nature Portfolio reporting summaries, source data, extended data, supplementary information, acknowledgements, peer review information; details of author contributions and competing interests; and statements of data and code availability are available at 10.1038/s41586-023-05790-6.

### Supplementary information

Supplementary Information Supplementary Methods, Supplementary Figs. 1–3, Supplementary Tables 1–7 and Supplementary References.

Reporting Summary

Supplementary Data Source data for Supplementary Table 2.

### Source data

Source Data Fig. 1

Source Data Fig. 2

Source Data Fig. 3

Source Data Fig. 4

Source Data Table 1

Source Data Extended Data Fig. 1

Source Data Extended Data Fig. 2

Source Data Extended Data Fig. 3

Source Data Extended Data Fig. 4

Source Data Extended Data Fig. 5

Source Data Extended Data Table 1

Source Data Extended Data Table 2

## Figures and Tables

**Extended Data Table 1:**
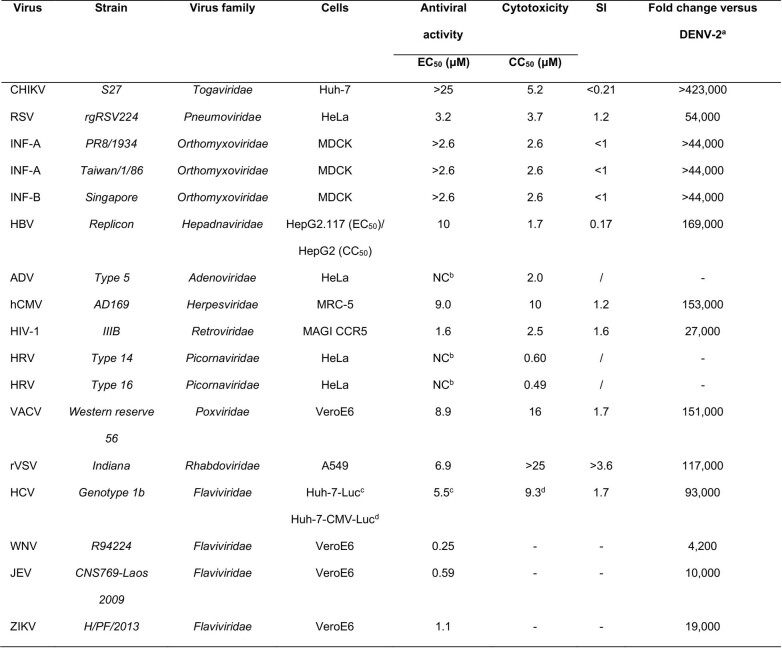
Antiviral activity of JNJ-1802 against various RNA and DNA viruses

EC_50_, 50% effective concentration; CC_50_, 50% cytotoxic concentration; SI, selectivity index (= CC_50_/EC_50_); NC, not calculated; ADV, adenovirus; CHIKV, chikungunya virus; HBV, hepatitis B virus; hCMV, human cytomegalovirus, HCV, hepatitis C virus; HIV-1, human immunodeficiency virus 1; HRV, human rhinovirus; INF-A, influenza A; INF-B, influenza B; JEV, Japanese encephalitis virus; RSV, respiratory syncytial virus; VACV, vaccinia virus; rVSV, recombinant vesicular stomatitis virus; WNV, West Nile virus; ZIKV, Zika virus. Number of experiments to calculate EC_50_ and CC_50_ was *n* = 1 except for the determination of the EC_50_ for HBV (*n* = 2), where mean values were shown. ^a^Fold change of the EC_50_ (respective strain)/EC_50_ (DENV-2/*16681*). EC_50_ (DENV-2/16681) = 0.059 nM (see also Table 1). ^b^EC_50_ was not calculated since antiviral activity was associated with similar level of cytotoxicity. ^c^Huh-7-Luc are HCV-Luc replicon containing cells used to measure the activity of the compound. ^d^Huh-7-CMV-Luc cells were used to measure the toxicity of the compound.

Source Data

**Extended Data Table 2:**
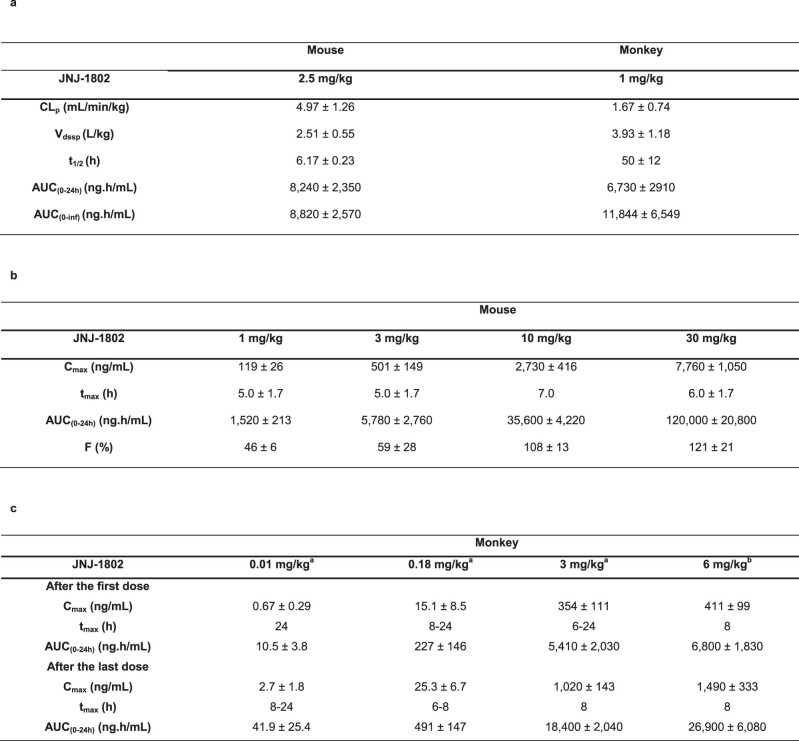
Pharmacokinetic parameters of JNJ-1802 in mice (**a, b**) and monkeys (rhesus macaques) (**a, c**) after intravenous (**a**) and oral dosing (**b-c**)

^a^Data are from female and male rhesus macaques (Biomedical Primate Research Center). ^b^Data are from male rhesus macaques (Walter Reed Army Institute of Research). JNJ-1802 was administered as a single dose to male CD-1 mice (6–8 weeks old) intravenously (**a**) as a solution formulated in PEG400/water (7:3) at 2.5 mg/kg or via oral gavage (**b**) as a solution formulated in PEG400/water (8:2) at 1, 3, 10 or 30 mg/kg. Values represent mean ± standard deviation (s.d) from 3 animals. F was calculated using AUC_(0-24h)_. JNJ-1802 was administered to female/male monkeys (adult) intravenously (**a**) as a solution formulated in PEG400/water (7:3) at 1.0 mg/kg or via oral gavage (**c**) for 12 or 14 days as a solution formulated in PEG400 at 0.01, 0.18, or 3 and 6 mg/kg. Values represent mean ± s.d. from 3 animals. CL_p_, plasma clearance; Vd_ssp_, Volume of distribution in plasma at steady state; t_1/2_, terminal phase elimination half-life; AUC, area under the plasma concentration versus time curve; AUC_(0-24h)_, AUC up to 24h; AUC_(0-inf)_, AUC curve to infinite time; C_max_, maximum plasma concentration; t_max_, the time to reach C_max_; F, bioavailability; PEG400, polyethylene glycol 400.

Source Data

## Data Availability

The genome sequence of DENV-2 RL has been deposited at GenBank (MW741553). The synthesis and chemical characterization of all compounds described in this paper is provided in the Supplementary Methods. The uncropped images of the western blots shown in Extended Data Fig. 2a,b,i are presented in Supplementary Figs. 1–3. All data supporting the findings of this study are available within the Article and its Supplementary Information. Source data are provided with this paper.

## Code availability

A custom script^66^ was used to derive the amino acid composition of each sample for all coding sequences per DENV genotype that was not specifically developed for this research but for all similar analyses. The code for the custom script is deposited as part of the pipeline VirVarSeq but is individually accessible on the Open Source software platform SourceForge (https://sourceforge.net/projects/virtools/?source=directory). The code for this specific variant detection script is ‘codon_table.pl’. The natural occurrence of the mutations was retrieved from the Virus Pathogen Resource database (www.viprbrc.org; accessed in May 2020). Graphs and figures were generated using Microsoft PowerPoint (v.2208, build 16.0.15601.20280), GraphPad Prism (v.9.0.0 and v.7.04) or Adobe Illustrator (2020 24.0.2); the software was made available by Janssen Pharmaceutical.
