# Supplementary material for: Blocking NS3–NS4B interaction inhibits dengue virus in non-human primates
Source: Nature. 2023 Mar 15;615(7953):678–86. doi: 10.1038/s41586-023-05790-6 (PMC10033419; doi:10.1038/s41586-023-05790-6)
Supplement: Supplementary file 1 — Supplementary Methods, Supplementary Figs. 1–3, Supplementary Tables 1–7 and Supplementary References. [file 41586_2023_5790_MOESM1_ESM.pdf]

nature portfolio

https://doi.org/10.1038/s41586-023-05790-6

Supplementary information

Blocking NS3–NS4B interaction inhibits dengue virus in non-human primates

In the format provided by the authors and unedited

Supplementary Methods

Additional cell lines - antiviral assays

Madin-Darby canine kidney (MDCK; American Type Culture Collection [ATCC]) cells were cultured in Ultra-MDCK serum-free medium (Lonza) and 20 µg/mL gentamicin (Gibco). The same medium was used in the antiviral experiments.

HeLa cell lines (cervical epithelial, human) were obtained from ATCC. In the experiments performed at Janssen, cells were cultured in Roswell Park Memorial Institute (RPMI)-1640 medium (Lonza) supplemented with 10% heat-inactivated fetal bovine serum (FBS; Thermo Fisher Scientific), 25 mM 4-(2-hydroxyethyl)-1-piperazineethanesulfonic acid (Hepes; Sigma), 100 mM L-glutamine (Sigma), and 20 µg/mL gentamicin. The same medium was used for the antiviral assay. The experiments performed at the Southern Research Institute (Frederick, MD) used Dulbecco's Modified Eagle Medium (DMEM; Lonza) supplemented with 5% FBS, 2 mM L-glutamine, 100 units/mL penicillin (Sigma), 100 µg/mL streptomycin (Sigma), and 0.1 mM nonessential amino acids (Sigma), while cells were cultured in 10% FBS.

HepG2.117 cells[66] were kindly provided by Prof. M. Nassal, University Hospital Freiburg, Freiburg, Germany. HepG2.117 cells and HepG2 cells were cultured in DMEM supplemented with 10% FBS, 2 mM L-glutamine, 20 µg/mL gentamicin, 80 µg/mL hygromycin (Roche), 500 µg/mL G418 (geneticin; Gibco), and 100 ng/mL doxycycline (Sigma). HepG2.117 is an inducible hepatitis B virus (HBV)-replication cell line[66]. It was established by introduction of a tetracycline (Tet)-responsive element (TRE)-controlled HBV genome (Genotype D, Subtype ayw) expression vector (pTRE-HBVT) into a HepG2 cell line that stably expresses a Tet-responsive trans-activator (tTA). HBV pregenomic RNA is transcribed under the control of the TRE-controlled minimal cytomegalovirus (CMV) promotor upon doxycycline removal from the culture medium, leading to capsid assembly and DNA synthesis.

The hepatoblastoma cell line HepG2 was obtained from ATCC and cultured in RPMI-1640 medium supplemented with 10% FBS, 25 mM Hepes, 2 mM L-glutamine, and 40 µg/mL gentamicin. Medium with 2% FBS was used in the antiviral assay.

Huh-7-Luc cells (Huh-7 human hepatoma cells that are stably transfected with a selectable self-replicating subgenomic hepatitis C virus [HCV] Genotype 1b [Clone ET] RNA sequence harboring a luciferase [Luc] reporter gene) and Huh-7-CMV-Luc (Huh-7 cells containing a CMV major immediate early promoter – Luc construct) were obtained from Heidelberg University Hospital (Heidelberg, Germany)[67]. Both cell lines were cultured in DMEM supplemented with 10% heat-inactivated FBS, 0.04% gentamicin (stock solution: 50 mg/mL), and 2 mM L-glutamine. For Huh-7-Luc cells, 0.25 mg/mL G418 was added. The same medium was used in the antiviral assay.

Human hepatocellular carcinoma cells (Huh-7)[68] were obtained from Prof. Heinz Schaller (Center for Molecular Biology Heidelberg [ZMBH], Germany). were cultured in DMEM supplemented with 10% heat-inactivated FBS, 0.02 mg/mL gentamicin, and 2 mM L-glutamine. Medium with 2% FBS was used in the chikungunya virus (CHIKV) assay.

MRC-5 cells (secondary human fetal lung fibroblast; ATCC) were cultured in DMEM supplemented with 10% FBS, 0.1 mM nonessential amino acids, 1.0 mM sodium pyruvate (Thermo Fisher), 2.0 mM L-glutamine, 100 units/mL penicillin, and 100 µg/mL streptomycin. The antiviral assay was performed at a reduced FBS concentration of 2%.

MAGI-CCR5 cells were obtained from the National Institutes of Health (NIH; Bethesda, MA) acquired immune deficiency syndrome (AIDS) Research and Reference Reagent Program. These cells were derived from HeLa-CD4-LTR-β-gal cells. The cells naturally express C-X-C chemokine receptor Type 4 (CXCR4) and have been engineered to express high levels of cluster

of differentiation (CD)4 and C-C chemokine receptor Type 5 (CCR5) and contain 1 copy of the HIV-1 LTR promoter driving expression of the β-galactosidase gene upon HIV-1 Tat transactivation. MAGI-CCR5 cells were cultured in DMEM with 10% FBS, supplemented with 2 mM L-glutamine and 0.1 mM nonessential amino acids. In the antiviral assay, DMEM was used supplemented with 2% FBS, 2 mM L-glutamine, 100 units/mL penicillin, 100 μg/mL streptomycin, and 0.1 mM nonessential amino acids.

Vero E6 cells (African green monkey kidney cells; ATCC) were cultured in Eagle's Minimal Essential Medium (MEM; Invitrogen) supplemented with 10% FBS, 2 mM L-glutamine, 100 units/mL penicillin, and 100 μg/mL streptomycin. Medium with 2% FBS was used in the antiviral assay.

Human lung epithelial A549 cells were obtained from ATCC and grown in DMEM with high glucose supplemented with 10% FBS and 2 mM L-glutamine. The same medium was used in the antiviral assay.

Chikungunya virus and INF virus assays

The antiviral activity of JNJ-1802 against CHIKV (S27) (*Togaviridae*), influenza (INF) A and INF B viruses (*Orthomyxoviridae*) was measured with a cytopathic effect (CPE) inhibition assay using ATPLite (PerkinElmer). This assay readout is based on the bioluminescent measurement of adenosine triphosphate (ATP) in metabolically active cells. Briefly, MDCK cells (6×10^3 cells/well) (INF-1 and B) or Huh-7 cells (8,000 cells/well) (CHIKV) were seeded in 384-well blackview plates (Costar) containing serially diluted test compound in cell culture medium (MDCK cells, Ultra-MDCK serum-free medium and 0.2 μg/mL gentamicin; Huh-7 cells, DMEM medium supplemented with 2% FBS) and were infected with CHIKV strain S27 at a multiplicity of infection (MOI) of 0.25, INF A/Taiwan/1/1986 (H1N1), INF

A/PuertoRico/8/1934 (H1N1), or INF B/Singapore/222/1979 (Yamagata lineage) virus strains at a MOI of 0.01. Plates were incubated at 37°C and 5% CO_2 for 2 days (CHIKV) until the viral CPE in the virus control wells reached ~100% or 3 days (INF A and B) until the CPE in the virus control wells reached ~90%. Then, ATPLite was added to all wells to assess the viability of the cells and thus the preventive effect of the antiviral test compound on CPE. Luminescence was measured using a Viewlux (PerkinElmer) apparatus. In parallel, cytotoxicity was assessed in non-infected Huh-7 cells (CHIKV) and non-infected MDCK cells (INF A and B) using the same ATP-based bioluminescent readout.

Respiratory syncytial virus assay

The antiviral activity of JNJ-1802 against wild-type rgRSV224, an engineered respiratory syncytial virus (RSV) (*Paramyxoviridae*), was determined by measuring inhibition of eGFP expression[69]. A viral titration assay was performed to determine the optimal virus dilution to be used for each newly produced batch of virus. The virus dilution resulting in an infection of 40% of the cells and an eGFP signal between 5×10^5 and 2×10^6 relative fluorescence units (RFU) per well in 96-well format was used. Briefly, HeLa cells (3,000 cells/well) and rgRSV224 (MOI = 1) were incubated for 3 days in 384-well black microtitre plates with a serial dilution of JNJ-1802 in triplicate (40 µL final volume; 0.5% DMSO) in RPMI-1640 supplemented with 10% FBS, 25 mM Hepes (Sigma), 10 mM L-glutamine, and 0.02 µg/mL gentamicin. eGFP fluorescence was measured using an automated scanning laser microscope. In parallel, cytotoxicity of the compound was measured in non-infected HeLa cells using ATP-based bioluminescent readout (ATPLite 1step luminescence assay system; PerkinElmer).

Hepatitis B virus assay

Briefly, 20,000 HepG2.117 cells per well were plated into 96-well plates 1 day before starting the 3-day incubation with JNJ-1802. During the antiviral testing, cells were cultured in DMEM medium with 1% MEM nonessential amino acid solution (Sigma) and 1% glutamine (Gibco) in the absence of doxycycline and presence of 2% FBS. JNJ-1802 was tested in a 1:4 serial dilution at 5 different concentrations in duplicate. At the end of the drug treatment, the supernatant was removed, and intracellular total DNA was extracted. Briefly, 100 µL of a 0.33% NP-40 solution was added per well, the plate was incubated at 4°C for 5 minutes and spun at 1,500 revolutions per minute (rpm) for 5 minutes to remove cell debris. Of the lysate, 35 µL was added to 65 µL QuickExtract DNA Extraction solution 1.0 (Epicentre) in a 96-well polymerase chain reaction (PCR) plate and the plate was incubated for 6 minutes at 65°C and 2 minutes at 98°C in a PCR machine. Of the extracted total DNA, 10 µL was used for the quantification of HBV DNA in a quantitative PCR (qPCR) assay. The Δ cycle threshold (Ct) method was used to calculate 50% effective concentration (EC_{50}) or 90% effective concentration (EC_{90}) values.

For toxicity testing, HepG2 cells (1×10^4 cells/well) were added to 96-well culture plates (Nunc) in RPMI-1640 medium with 2% L-glutamine (Sigma) in the presence of 2% FBS containing serially diluted test compound in cell culture medium and were incubated for 4 days. Cytotoxicity of JNJ-1802 was measured using the ATPLite kit (Perkin Elmer).

Hepatitis C virus assay

The antiviral activity of JNJ-1802 against HCV (*Flaviviridae*) was tested in an HCV replicon-containing cell culture system consisting of Huh-7 cells that are stably transfected with a selectable self-replicating sub-genomic HCV Genotype 1b (Clone ET) RNA sequence harboring a luciferase reporter gene (Huh-7-Luc cells)[67]. In brief, Huh-7 Luc replicon-containing cells were

seeded in 384-well plates (2,500 cells/well) and incubated for 3 days with a concentration range of serially diluted JNJ-1802 in a final DMSO concentration of 0.5% in cell culture medium without G418. HCV replicon RNA replication was determined by means of measuring the firefly luciferase reporter gene expression using the SteadyLite Plus assay kit (PerkinElmer) and luminescence measurement using a ViewLux reader (PerkinElmer). A toxicity assay was performed using the Huh-7-CMV-Luc cells (containing an hCMV-MIEP-Luc construct). Cells were seeded in 384-well culture plates (2,500 cells/well) in cell culture medium without G418 and incubated for 3 days at 37°C in a humidified 5% CO_2 atmosphere in the presence or absence of 4-fold serially diluted compound. Luciferase activity was quantified using the SteadyLite Plus assay kit (PerkinElmer) and luminescence measurement on a ViewLux reader (PerkinElmer).

Adenovirus type 5 and HRV assay

JNJ-1802 was tested against adenovirus (ADV) Type 5 (*Adenoviridae*), human rhinovirus (HRV)-14 or HRV-16 (*Picornaviridae*) using a cytoprotection-based assay in HeLa cells. Briefly, HeLa cells (2.5×103 cells/well) and ADV, HRV-14 or HRV-16 were mixed in the presence of a serial dilution of the compound and incubated for 6 days (ADV) or 3 to 4 days (HRV-14 and -16). Inhibition of viral replication and cytotoxicity were measured using a 3 (4,5 dimethylthiazol-2 yl)-5 (3 carboxymethoxyphenyl)-2 (4 sulfophenyl)-2H tetrazolium (MTS) dye reduction assay (CellTiter, Promega). MTS is metabolised by the mitochondrial enzymes of metabolically active cells to yield a soluble formazan product, enabling the rapid quantitative analysis of cell viability and compound cytotoxicity. MTS is a stable solution that does not require preparation before use. At termination of the assay, 15 µL of MTS reagent was added per well. The microtitre plates were then incubated for 1.5 to 2 hours at 37°C. The plates were read

spectrophotometrically at 490/650 nm with a Molecular Devices SpectraMax plate reader. In parallel, cytotoxicity was determined using the same assay principle in the absence of virus.

Human cytomegalovirus and VACV assays

The antiviral activity of JNJ-1802 against human cytomegalovirus (hCMV) (*Herpesviridae*) was evaluated in MRC-5 cells and against vaccinia virus (VACV) (*Poxviridae*) in Vero E6 cells using a plaque reduction assay. MRC-5 cells (1×10^5 cells/well) were seeded in 24-well plates (Corning) or Vero E6 cells (3.2×10^5 cell/well) in 12-well plates (Corning) and incubated. The following day, media was aspirated and 100 plaque-forming units (PFU) of hCMV AD169 were added. After 1 h of viral absorption, a serially diluted JNJ-1802 solution containing 0.5% methylcellulose (Sigma) was added. The plates were incubated for 6 days. VACV was added at 150 PFU to the corresponding wells in presence of serial dilutions (in duplicate) of JNJ-1802. After incubation for 1 h at 37°C and 5% CO_2, a compound dilution / overlay media mixture was added to the wells. Plates were incubated for 72 h. Next, media was aspirated, and cells were fixed using 20% methanol containing crystal violet (Sigma). Plaques were counted by microscopic inspection. In parallel, compound cytotoxicity was assessed in MRC-5 (hCMV) or Vero E6 (VACV) cells seeded in 96-well plates (1×10^4 cells/well). After an overnight incubation, JNJ-1802 was added. After a 6-day (MRC-5 cells) or 72-hour (Vero E6 cells) incubation period, cell viability was measured using the MTS dye reduction assay. At termination of the assay, 20 µL of MTS reagent was added per well. The microtitre plates were then incubated for approximately 1.5 h at 37°C. The plates were read spectrophotometrically at 490/650 nm with a Molecular Devices Vmax plate reader.

Human immunodeficiency virus assay

The antiviral activity of JNJ-1802 against HIV 1 (*Retroviridae*) was assessed in MAGI CCR5 cells containing an HIV 1 LTR promoter driving expression of the β-galactosidase gene upon HIV 1 Tat transactivation. Briefly, MAGI CCR5 cells were pre-seeded 1 day before infection (1×10^4 cells/well) and incubated overnight. Next, MAGI CCR5 cells were infected with HIV 1/IIIB (0.001 50% tissue culture infective dose per cell) in the presence of a serial dilution (triplicate) of JNJ-1802. After 48 h incubation, antiviral activity was measured as the inhibition of β-galactosidase reporter expression using Gal screen reagent (Tropix) according to the manufacturer's instructions. The resulting chemiluminescence signal was read using a Microbeta Trilux luminescence reader (PerkinElmer, Wallac). In parallel, cytotoxicity plates were measured using the MTS method as described above.

Vesicular stomatitis virus assay

Inhibition of vesicular stomatitis virus (VSV; *Rhabdoviridae*) replication by JNJ-1802 was assessed in A549 cells infected with recombinant (r)VSV harboring a luciferase reporter gene. In brief, A549 cells (3×10^4 cells/well) were seeded 1 day in advance, followed by a 1-hour incubation with a serial dilution of JNJ-1802, before infection with rVSV. After 24-hour incubation, BrightGlo reagent (Promega) was added and luciferase activity was measured using an Envision plate reader (PerkinElmer). In parallel, cytotoxicity was assessed in non-infected A549 cells using the commercially available CellTiter-Glo Luminescent Cell Viability Kit (Promega). The procedure involves adding the single reagent (CellTiter-Glo Reagent) directly to the cells, which induces cell lysis and the production of a bioluminescent signal that is proportional to the amount of ATP present (which is a biomarker for viability).

Evaluation of haematological parameters

At the clinical laboratory at the Biomedical Primate Research Centre (BPCR), haematological parameters were measured in EDTA-treated blood (1 mL was collected) using a Sysmex XT-2000iV Automated Hematology Analyzer (Sysmex® Corporation of America). The data obtained are summarized in Supplementary Tables 4–7. Haematological data were compared to the minimum and maximum normal values also shown in the tables. Normal values (minimum and maximum) were determined using blood samples collected from healthy male and female rhesus macaques from the BPRC breeding colony and were calculated using haematological values from > 50 individual animals. As the normal values were obtained by averaging the values from these animals, haematological values of individual animals may deviate. Indeed, data were obtained at several time points from individual animals that were outside the normal minimum and maximum values. These values are indicated in the table (orange shading). However, these changes in haematological parameters could not be related to the experimental infection or compound dose.

Chemical characterization of JNJ-1802

All commercial reagents were used without further purification. Dry solvents were used. NMR experiments were carried out using a Bruker Avance III 400 or a Bruker 360 DPX spectrometer, at ambient temperature (298.6 K), using internal deuterium lock, and equipped with reverse double-resonance (^{1}H, ^{13}C, SEI) probe head with z gradients, and operating at 400 MHz or 360 MHz for the proton, and 100 Hz or 90 Hz for the carbon, respectively. Chemical shifts (δ) are reported in parts per million (ppm). J values are expressed in Hz. The following abbreviations were used for multiplicities: s = singlet, d = doublet, t = triplet, q = quartet, m = multiplet, dd = doublet of doublets, td = triple doublet, dt = double triplet, and br = broad.

Chromatographic experiments for HRMS were performed using an Ultimate 3000 RS UHPLC system (Thermo Fisher Scientific, Germering, Germany) composed of a gradient pump, an autosampler, a column oven, and a diode-array detector (DAD). The DAD scanning wavelength ranged from 200 to 400 nm. Mobile phase A consisted of 10 mM CH_3COONH_4 in 95 % H_2O + 5 % CH_3CN, and mobile phase B consisted of CH_3CN. The liquid chromatography (LC) experiments were carried out at a flow rate of 0.6 mL/min. A linear gradient was applied from 95 % A to 5 % A in 2.10 min and held for 1.9 min. The column compartment was kept at 55 °C. A 2.1 mm i.d. x 100 mm Acquity UPLC BEH C18 column packed with 1.7 um particles was obtained from Waters Corporation (Milford, MA, USA). Flow from the column was 1:10 split to the mass spectrometer (MS).

The high-resolution mass spectrometry experiments were performed on a Q-Exactive mass spectrometer (Thermo Fisher Scientific, Bremen, Germany) via an electrospray ionization (ESI) interface and in Full MS scan type mode. Nitrogen was used as the nebulizer gas. The MS was operated both in positive and negative mode, and the ESI parameters were as follows: spray

voltage: 4.00 kV; capillary temperature: 320 °C; S-lens RF level: 50.0. Masses in the m/z 150 to 1200 range were selected and the experiments were performed at resolution of 140,000. The data acquisition software used was Xcalibur (version 4.4, Thermo Fisher Scientific). The MS was calibrated in both modes according to the manufacturer instructions. The reported accurate masses correspond to the $[M+H]^+$ (protonated monoisotopic molecular mass) and/or $[M-H]^-$ (deprotonated monoisotopic molecular mass).The LC-MS analyses were performed using a LC pump, a diode-array (DAD) or a UV detector and a column as specified in the respective methods. If necessary, additional detectors were included (see methods below). Flow from the column was brought to the MS which was configured with an atmospheric pressure ion source. Data acquisition was performed with appropriate software. If not specified differently in the data, the reported molecular ion corresponds to the $[M+H]^+$ (protonated molecule). For molecules with multiple isotopic patterns (Br, Cl), the reported value is the one obtained for the lowest isotope mass. All results were obtained with experimental uncertainties that are commonly associated with the method used. "SQD" means Single Quadrupole Detector, "DAD" Diode Array Detector, "HSS" High Strength Silica.

LCMS1 conditions: reversed-phase UPLC-DAD and SQD was carried out on a HSS T3 column (1.8 µm, 2.1 × 100 mm) from Waters with a flow rate of 0.7 mL/min at 55 °C. The gradient conditions used were as follows: 100 % A (10 mM CH_3COONH_4 in 95 % H_2O + 5 % CH_3CN), 0 % B (CH_3CN), to 5 % A in 2.1 min, to 0 % A in 0.9 min, to 5 % A in 0.5 min, with a total run time of 3.5 min.

LCMS2 conditions: reversed-phase UPLC-DAD and SQD was carried out on a BEH C18 column (1.7 µm, 2.1 × 50 mm) from Waters with a flow rate of 0.8 mL/min at 55 °C. The

gradient conditions used were as follows: 95 % A (10 mM CH_3COONH_4 in 95 % H_2O + 5 % CH_3CN), 5 % B (CH_3CN), to 5 % A in 1.3 min, held for 0.7 min, with a total run time of 2 min.

The SFC measurement was performed using an Analytical Supercritical fluid chromatography (SFC) system composed by a binary pump for delivering carbon dioxide (CO_2) and modifier, an autosampler, a column oven, a diode array detector equipped with a high-pressure flow cell standing up to 400 bars. If configured with a MS the flow from the column was brought to the MS. Data acquisition was performed with appropriate software.

SFC conditions: SFC was carried out on a Daicel Chiralpak AS-3 column (3 µm, 4.6 x 150 mm) with a flow rate of 2.5 mL/min and a backpressure of 110 bars, at 40 °C. The gradient conditions used were as follows: 90 % A (CO_2), 10 % B (EtOH + 0.2 % $iPrNH_2$ + 3 % H_2O) to 50 % A, 50 % B in 6 min, held for 3.5 min, with a total run time of 9.5 min.

For melting points, values are peak values and are obtained with experimental uncertainties that are commonly associated with this analytical method. Melting points were determined with a DSC823e (Mettler-Toledo). Melting points were measured with a temperature gradient of 10 °C/minute. Maximum temperature was 300 °C.

Optical rotations were measured on a Perkin-Elmer 341 polarimeter with a sodium lamp. The rotation is reported in degrees.

Synthesis of JNJ-1802

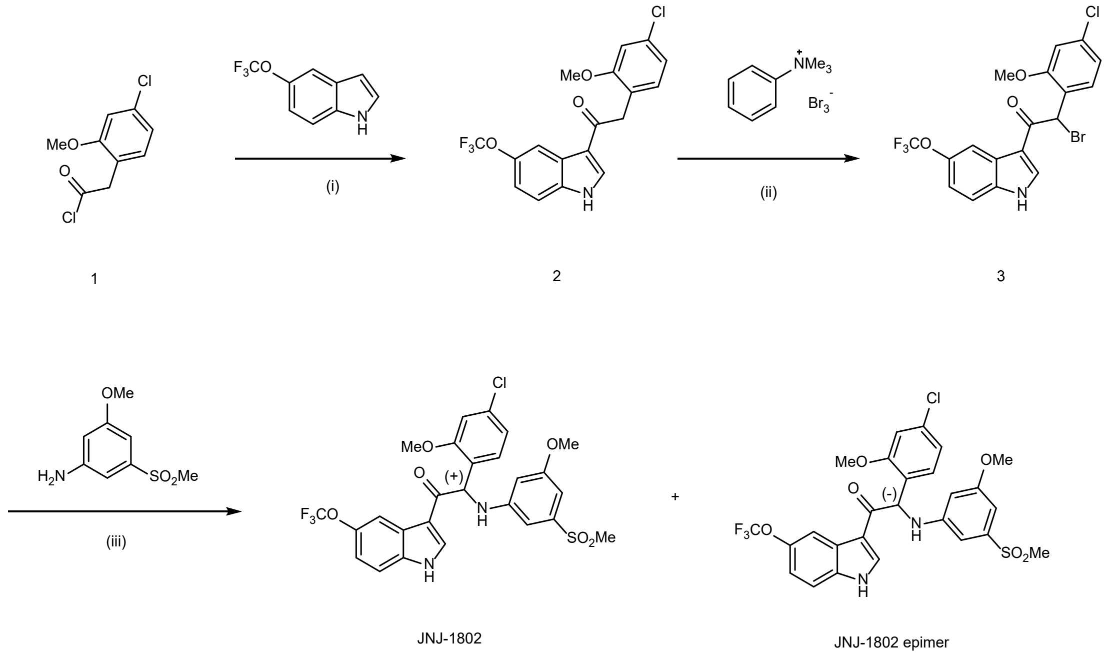

Scheme 1. Synthesis pathway for JNJ-1802: (i) Et_2AlCl, CH_2Cl_2, 0 °C to r.t., 4 h; (ii) THF, 0 °C to r.t., 2 h; (iii) DIPEA, CH_3CN, 90 °C, 18 h, followed by chiral separation.

2-(4-Chloro-2-methoxyphenyl)-1-(5-(trifluoromethoxy)-1H-indol-3-yl)ethan-1-one (2)

A solution of 5-(trifluoromethoxy)-*1H*-indole ([CAS 262593-63-5], 3 g, 14.9 mmol) in CH_2Cl_2 (150 mL) was cooled to 0 °C under nitrogen atmosphere. A solution of diethylaluminum chloride (1 M in hexane, 22.4 mL, 22.4 mmol) was added dropwise and the resulting mixture was kept at 0 °C for 15 min. A solution of 2-(4-chloro-2-methoxyphenyl)acetyl chloride[70] 1 (4.57 g, 20.9 mmol) in CH_2Cl_2 (100 mL) was added dropwise. Stirring was continued at 0 °C for 1 h and the reaction mixture was subsequently stirred at room temperature for 4 h. The reaction mixture was poured out in a stirring ice/Rochelle salt solution. After the ice had melted, the mixture was filtered over dicalite® and the filter cake was washed several times with THF. The filtrates were

combined. The layers were separated and the organic layer was washed with brine, dried over $MgSO_4$, filtered, and evaporated under reduced pressure. The residue was triturated with CH_2Cl_2 (50 mL). The resulting precipitate was filtered and dried under vacuum at 50 °C to provide 2-(4-chloro-2-methoxyphenyl)-1-(5-(trifluoromethoxy)-*1H*-indol-3-yl)ethan-1-one **2** (4.39 g, yield: 76 %).

^{1}H NMR (400 MHz, DMSO-*d6*) δ ppm 3.75 (s, 3 H), 4.18 (s, 2 H), 6.97 (dd, J=8.1, 2.0 Hz, 1 H), 7.05 (d, J=2.0 Hz, 1 H), 7.21 (m, J=7.4, 7.4 Hz, 2 H), 7.59 (d, J=8.8 Hz, 1 H), 8.03 (s, 1 H), 8.56 (s, 1 H), 12.21 (br s, 1 H); ^{13}C NMR (101 MHz, DMSO-*d6*) δ ppm 192.5, 158.5, 144.2, 136.4, 135.5, 132.9, 132.5, 126.3, 124.0, 120.4, 116.9, 116.6, 114.0, 113.6, 111.7, 56.3 (1 peak is hidden under the solvent peak); LC-MS: $[M+H]^+$ 384; purity 100 % (method LCMS2); Melting point: 221-235 °C (DSC peak: 225.7 °C); HRMS (ESI^+) m/z: $[M]^+$ calcd for $C_{18}H_{14}O_3NClF_3$, 384.0609; found, 384.0606

2-Bromo-2-(4-chloro-2-methoxyphenyl)-1-(5-(trifluoromethoxy)-1H-indol-3-yl)ethan-1-one (3)

A stirred solution of 2-(4-chloro-2-methoxyphenyl)-1-(5-(trifluoromethoxy)-*1H*-indol-3-yl)ethan-1-one **2** (4.39 g, 11.4 mmol) in THF (200 mL) was cooled to 0 °C. A solution of phenyltrimethylammonium tribromide ([CAS 4207-56-1], 4.73 g, 12.6 mmol) in THF (100 mL) was added dropwise. The resulting suspension was stirred at room temperature for 2 h. The solids were removed by filtration and washed with THF. The combined filtrates were evaporated under reduced pressure. The residue was mixed with EtOAc (30 mL). The solids were isolated by filtration, washed with a small amount of EtOAc, and dried under vacuum at 50 °C to provide 2-bromo-2-(4-chloro-2-methoxyphenyl)-1-(5-(trifluoromethoxy)-*1H*-indol-3-yl)ethan-1-one **3** (5.0 g, yield: 95 %) as a white solid, which was used in the next step without further purification.

^1H NMR (360 MHz, DMSO-d_6) δ ppm 3.89 (s, 3 H), 6.89 (s, 1 H), 7.07 (dd, J=8.2, 2.0 Hz, 1 H), 7.15 (d, J=1.5 Hz, 1 H), 7.24 (dd, J=8.6, 2.0 Hz, 1 H), 7.57 (d, J=8.4 Hz, 1 H), 7.61 (d, J=8.8 Hz, 1 H), 8.05 (s, 1 H), 8.49 (d, J=3.3 Hz, 1 H), 12.39 (br d, J=2.6 Hz, 1 H); LC-MS: R_t 2.29 min, $[M+H]^+$ 462; purity: 98 % (method LCMS1)

(+)-2-(4-chloro-2-methoxyphenyl)-2-((3-methoxy-5-(methylsulfonyl)phenyl)amino)-1-(5-(trifluoromethoxy)-1H-indol-3-yl)ethan-1-one (JNJ-1802)

A mixture of 2-bromo-2-(4-chloro-2-methoxyphenyl)-1-(5-(trifluoromethoxy)-*1H*-indol-3-yl)ethan-1-one **3** (2.5 g, 5.40 mmol), 3-methoxy-5-(methylsulfonyl)aniline ([CAS 62606-02-4], 1.49 g, 7.38 mmol), and diisopropylethylamine (931 µL, 5.40 mmol) in CH_3CN (100 mL) was stirred overnight at 90 °C. The reaction mixture was concentrated under reduced pressure. The residue was dissolved in CH_2Cl_2 (100 mL), washed with aqueous HCl (1 N in water, 100 mL) and water (100 mL), dried over $MgSO_4$, filtered, and evaporated under reduced pressure. The residue was purified by column chromatography (Stationary phase: Grace Reveleris® silica 120 g, Mobile phase: EtOAc:EtOH (3:1)/heptane gradient from 0/100 to 50/50). The desired fractions were combined and evaporated under reduced pressure. The residue was precipitated from EtOAc (10 mL) while stirring. The solids were isolated by filtration and washed with a small amount of EtOAc to provide 2-(4-chloro-2-methoxyphenyl)-2-((3-methoxy-5-(methylsulfonyl)phenyl)amino)-1-(5-(trifluoromethoxy)-*1H*-indol-3-yl)ethan-1-one (477 mg) as a racemic mixture. The filtrate was evaporated under reduced pressure and the residue was taken up with EtOAc (5 mL). After overnight stirring, the solids were isolated by filtration and washed with EtOAc to provide a second crop of 2-(4-chloro-2-methoxyphenyl)-2-((3-methoxy-5-(methylsulfonyl)phenyl)amino)-1-(5-(trifluoromethoxy)-*1H*-indol-3-yl)ethan-1-one (216 mg). Both batches of the racemic mixture were combined and the mixture was separated into its

enantiomers by normal phase chiral purification (Stationary phase: AS 20 µm, Mobile phase: 100 % MeOH). The first eluted epimer was stirred up in water (2 mL) and MeOH (3 mL) at 40 °C. The solids were filtered, washed (3 x) with H_2O/MeOH 1/1, and dried under vacuum at 45 °C to provide (+)-2-(4-chloro-2-methoxyphenyl)-2-((3-methoxy-5-(methylsulfonyl)phenyl)amino)-1-(5-(trifluoromethoxy)-*1H*-indol-3-yl)ethan-1-one (**JNJ-1802**) (151 mg, yield: 5 %). The second eluted epimer was further purified by flash chromatography on silica gel (Stationary phase: Grace Reveleris® silica 12 g, Mobile phase: heptane/EtOAc/EtOH 100/0/0 to 40/45/15). The desired fractions were combined, evaporated under reduced pressure, and co-evaporated with EtOAc. The residue was stirred up in MeOH (5 mL) and precipitated by the slow addition of H_2O (4 mL). The solids were filtered, washed (3 x) with H_2O/MeOH 1/1, and dried under vacuum at 50 °C to provide the **epimer of JNJ-1802** (132 mg, yield: 4 %).

JNJ-1802:

^{1}H NMR (400 MHz, DMSO-d_6) δ ppm 3.09 (s, 3 H) 3.73 (s, 3 H) 3.99 (s, 3 H) 6.26 35 (d, J=7.9 Hz, 1 H) 6.55 - 6.62 (m, 2 H) 6.91 (t, J=1.5 Hz, 1 H) 6.98 (dd, J=8.4, 2.0 Hz, 1 H) 7.07 (d, J=7.9 Hz, 1 H) 7.13 (d, J=2.0 Hz, 1 H) 7.21 (dd, J=8.8, 1.8 Hz, 1 H) 7.36 (d, J=8.4 Hz, 1 H) 7.59 (d, J=8.8 Hz, 1 H) 8.07 (d, J=0.9 Hz, 1 H) 8.55 (s, 1 H) 12.29 (br s, 1 H); ^{13}C NMR (DMSO-d_6, 101 MHz) δ ppm 190.8, 160.9, 157.6, 149.1, 144.5, 142.9, 136.4, 135.5, 133.8, 129.5, 126.6, 126.4, 121.3, 117.3, 114.4, 114.3, 113.4, 112.2, 103.2, 100.2, 56.8, 55.8, 55.0, 43.9, (CF_3 quadruplet not visible); LC/MS: R_t 1.20 min, $[M+H]^+$ 583; purity 97 % (method LCMS2); Chiral SFC: R_t 3.10 min, $[M+H]^+$ 583, chiral purity 100 %; $[\alpha]_D^{20}$: +130.3° (589 nm, c 0.555 w/v %, DMF, 20 °C); Melting point: 106-118 °C (DSC peak: 111.5 °C); HRMS (ESI$^+$) m/z: $[M]^+$ calcd for $C_{26}H_{23}O_6N_2ClF_3S$, 583.0912; found 583.0907; Elemental analysis requires C, 53.57 %; H, 3.80 %; N, 4.81 %; found C, 53.3 %; H, 3.9 %; N, 4.8 %

Epimer of **JNJ-1802**:

^{1}H NMR (400 MHz, DMSO-d_6) δ ppm 3.09 (s, 3 H) 3.73 (s, 3 H) 3.99 (s, 3 H) 6.26 (d, J=7.9 Hz, 1 H) 6.56 - 6.62 (m, 2 H) 6.92 (t, J=2.0 Hz, 1 H) 6.98 (dd, J=8.1, 2.0 Hz, 1 H) 7.07 (d, J=7.9 Hz, 1 H) 7.13 (d, J=2.0 Hz, 1 H) 7.22 (dd, J=8.8, 1.8 Hz, 1 H) 7.36 (d, J=8.4 Hz, 1 H) 7.59 (d, J=8.8 Hz, 1 H) 8.07 (d, J=0.9 Hz, 1 H) 8.55 (s, 1 H) 12.30 (br s, 1 H); ^{13}C NMR (DMSO-d_6, 101 MHz) δ ppm 190.8, 160.9, 157.6, 149.1, 144.5, 142.9, 136.5, 135.5, 133.8, 129.5, 126.6, 126.4, 121.3, 120.8 (q, J=255.0 Hz), 117.3, 120.8, 114.4, 114.3, 113.4, 112.2, 104.5, 103.2, 100.2, 56.8, 55.8, 55.0, 43.9; LC/MS: R_t 1.20 min, $[M+H]^+$ 583; purity 100 % (method LCMS2); Chiral SFC: R_t 3.51 min, $[M+H]^+$ 583, chiral purity 100 %; $[\alpha]_D^{20}$: -133.2° (589 nm, c 0.5 w/v %, DMF, 20 °C); Melting point: 104-115 °C (DSC peak: 110.2 °C); HRMS (ESI$^+$) m/z: $[M]^+$ calcd for $C_{26}H_{23}O_6N_2ClF_3S$, 583.0912; found, 583.0907.

^{1}H, ^{13}C nuclear magnetic resonance (NMR), and differential scanning calorimetric (DSC) characterisation of the compounds

Intermediate 2 – ^{1}H NMR

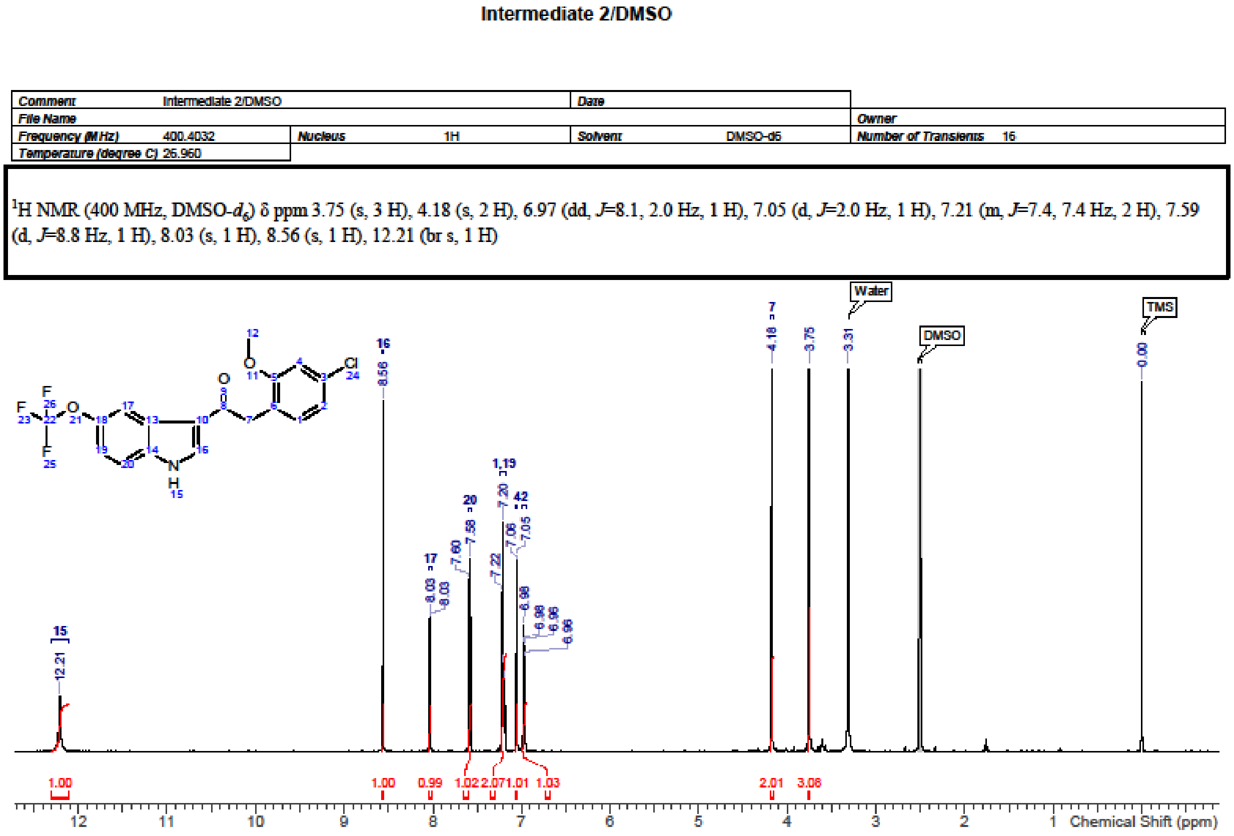

Intermediate 2 – ^{13}C NMR

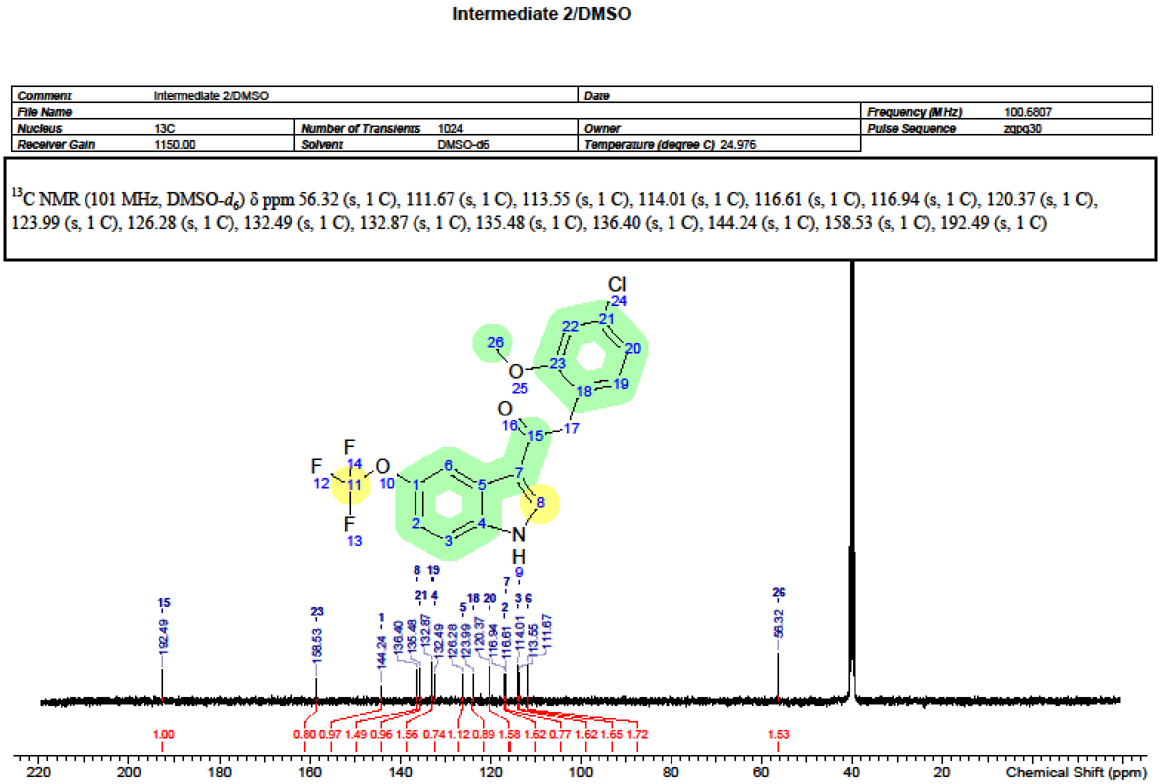

Intermediate 2 – LC-MS chromatogram

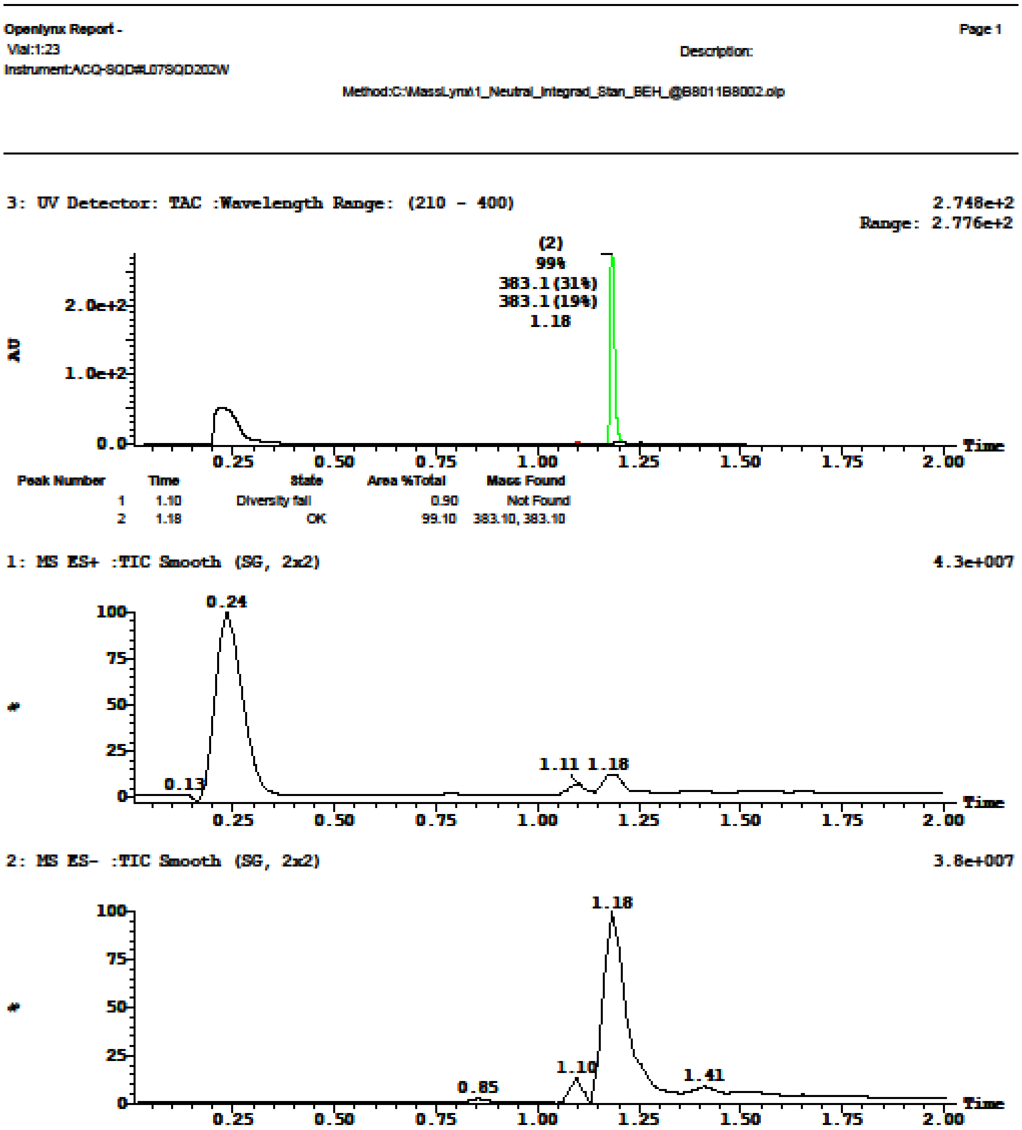

Peak Number	Time	State	Area %Total	Mass Found
1	1.10	Diversity fail	0.90	Not Found
2	1.18	OK	99.10	383.10, 383.10

Intermediate 2 – DSC trace

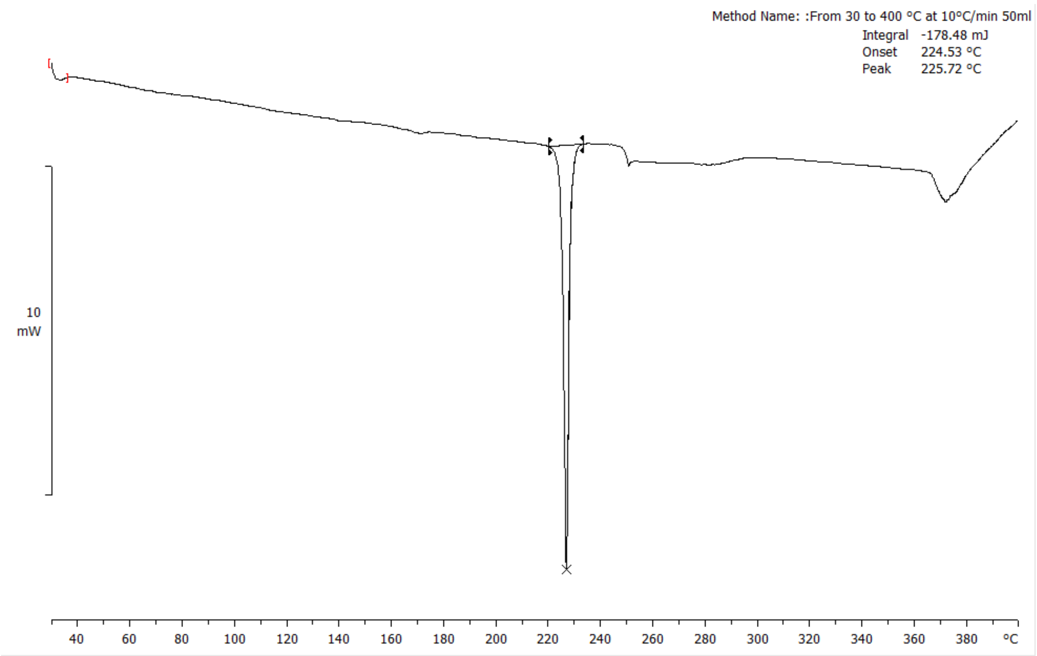

Intermediate 3 – ^{1}H NMR

Intermediate 3/DMSO

Comment	Intermediate 3/DMSO						
File Name						*Owner*	
Frequency (MHz)	360.1328	*Nucleus*	1H	*Solvent*	DMSO-d6	*Number of Transients*	16
Temperature (degree C)	27.000						

^{1}H NMR (360 MHz, DMSO-d_6) δ ppm 3.89 (s, 3 H), 6.89 (s, 1 H), 7.07 (dd, J=8.2, 2.0 Hz, 1 H), 7.15 (d, J=1.5 Hz, 1 H), 7.24 (dd, J=8.6, 2.0 Hz, 1 H), 7.57 (d, J=8.4 Hz, 1 H), 7.61 (d, J=8.8 Hz, 1 H), 8.05 (s, 1 H), 8.49 (d, J=3.3 Hz, 1 H), 12.39 (br d, J=2.6 Hz, 1 H)

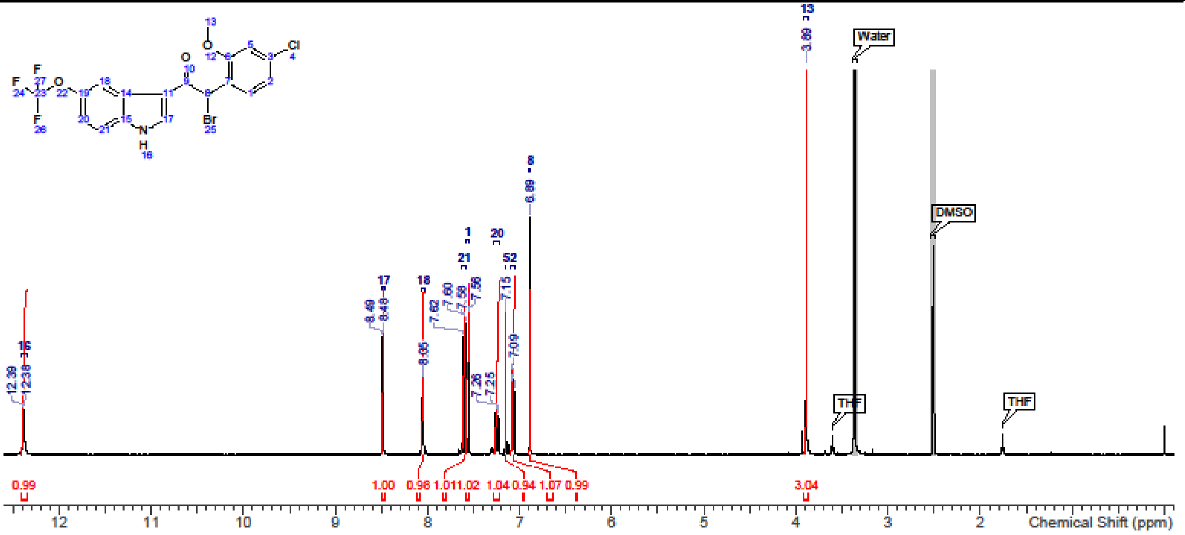

Intermediate 3 – LC-MS chromatogram

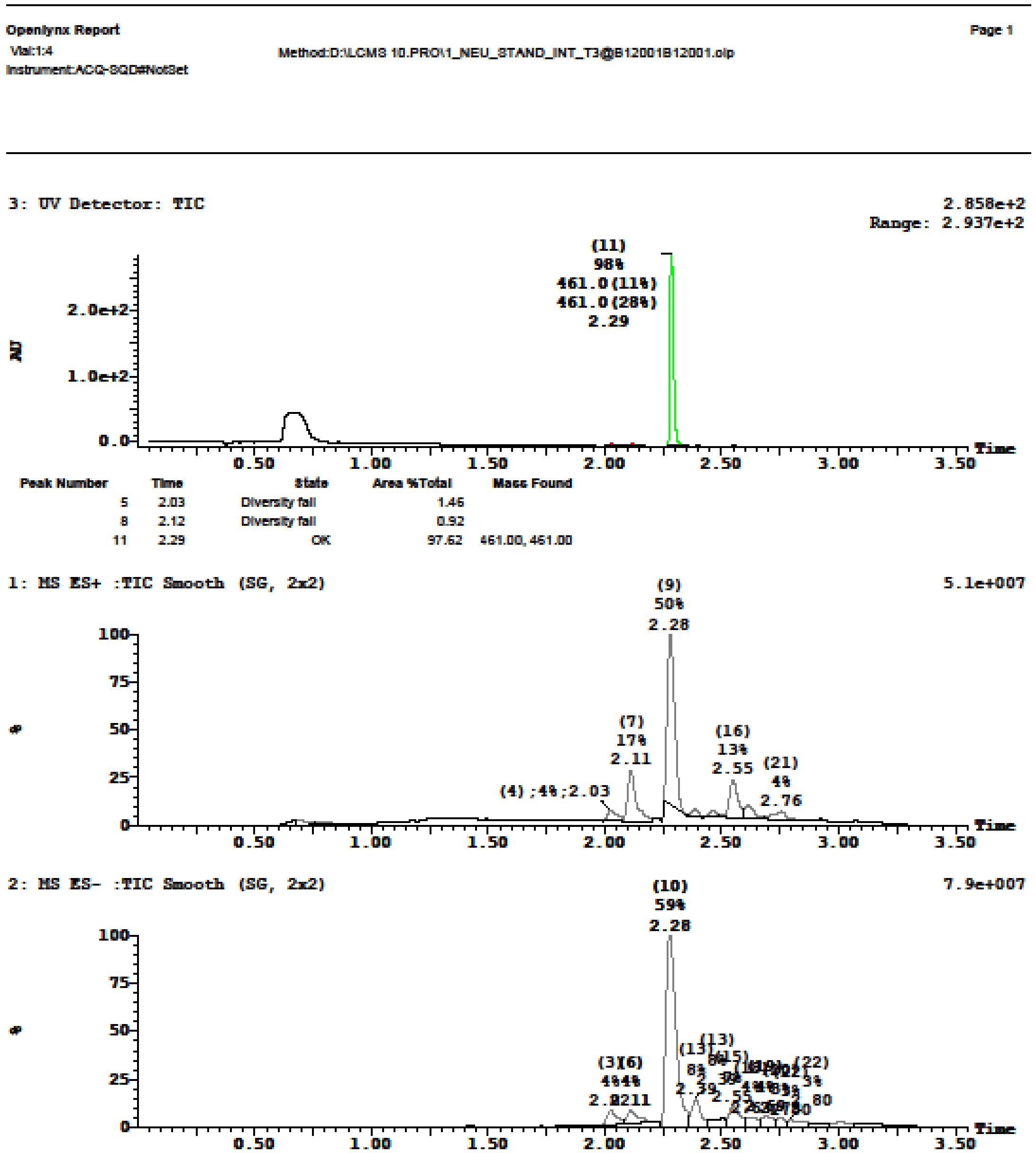

JNJ-1802 – ^{1}H NMR

JNJ-1802/DMSO

Comment	JNJ-1802/DMSO						
File Name						Owner	
Frequency (MHz)	400.4032	Nucleus	1H	Solvent	DMSO-d6	Number of Transients	16
Temperature (degree C)	27.060						

^{1}H NMR (400 MHz, DMSO-d_6) δ ppm 3.09 (s, 3 H), 3.73 (s, 3 H), 3.99 (s, 3 H), 6.26 (d, *J*=7.9 Hz, 1 H), 6.59 (m, *J*=4.0 Hz, 2 H), 6.91 (br s, 1 H), 6.98 (dd, *J*=8.4, 2.0 Hz, 1 H), 7.07 (d, *J*=7.9 Hz, 1 H), 7.13 (d, *J*=2.0 Hz, 1 H), 7.21 (dd, *J*=8.8, 1.8 Hz, 1 H), 7.36 (d, *J*=8.4 Hz, 1 H), 7.59 (d, *J*=8.8 Hz, 1 H), 8.06 (d, *J*=0.9 Hz, 1 H), 8.55 (s, 1 H), 12.29 (br s, 1 H)

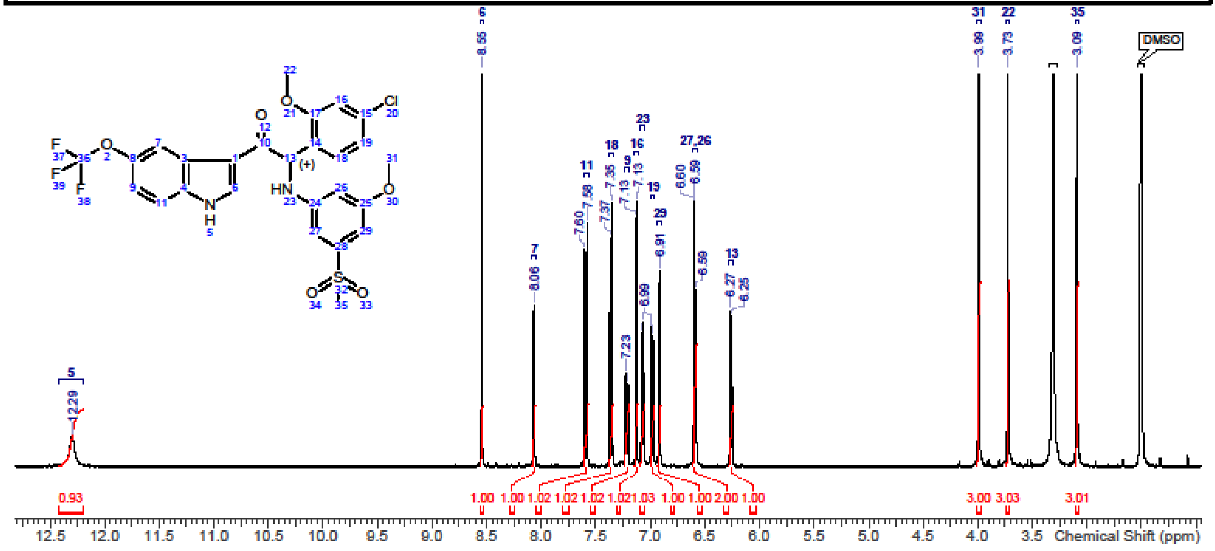

JNJ-1802 – ^{13}C NMR

JNJ-1802-AAA/DMSO

Comment					
File Name					
Frequency (MHz)	100.6807	Nucleus	13C	Number of Transients	1024
Owner	shr-nmrbdls2	Pulse Sequence	zgpg30	Receiver Gain	1150.00
Solvent	DMSO-d6	Temperature (degree C)	24.976		

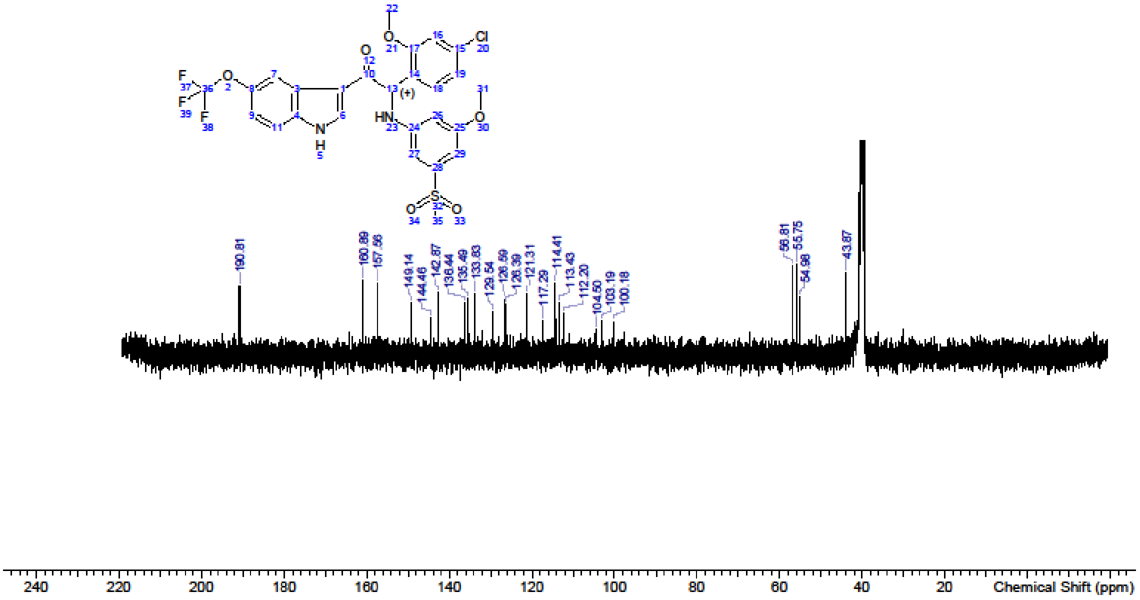

JNJ-1802 – LC-MS chromatogram

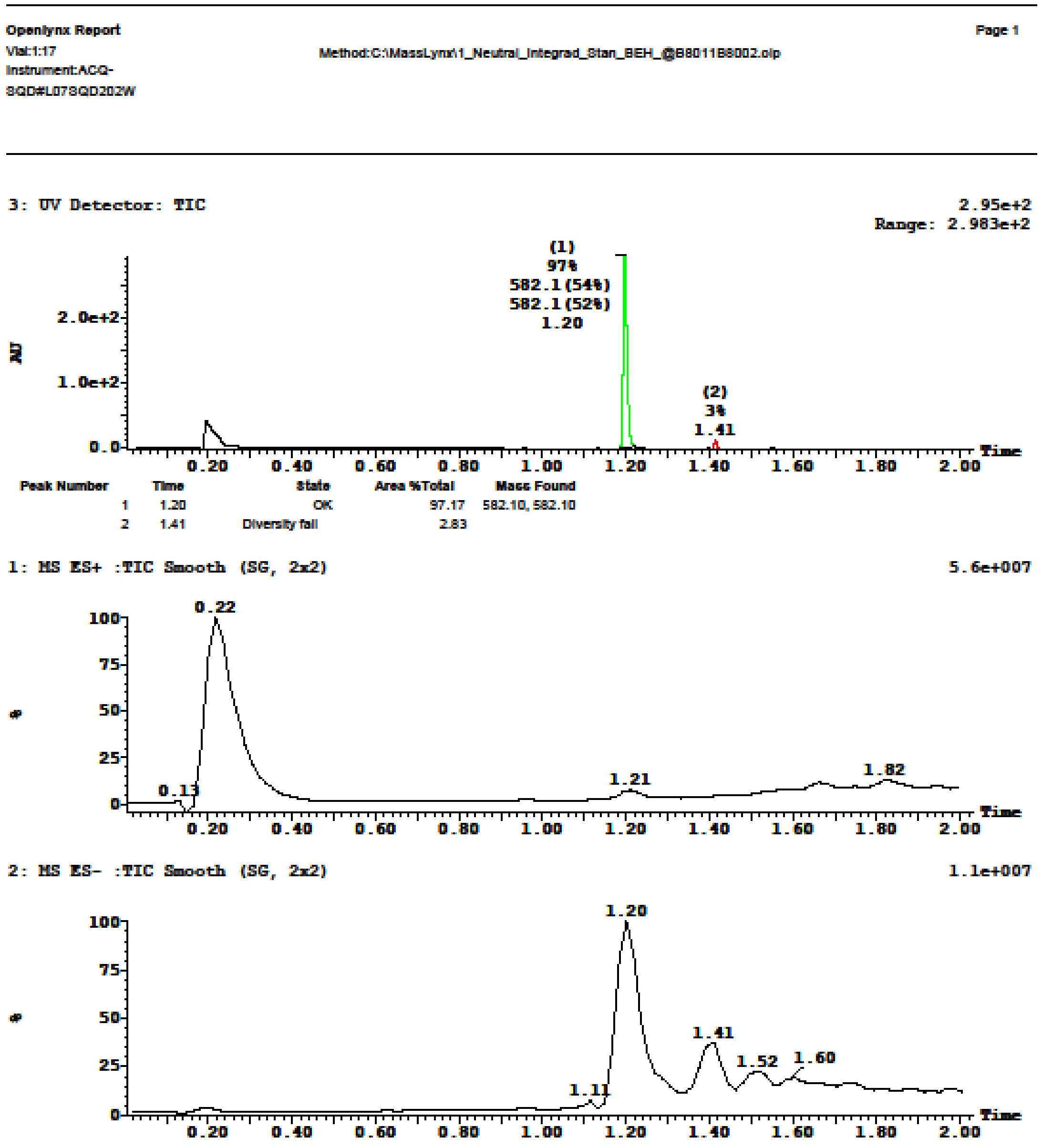

JNJ-1802 – DSC trace

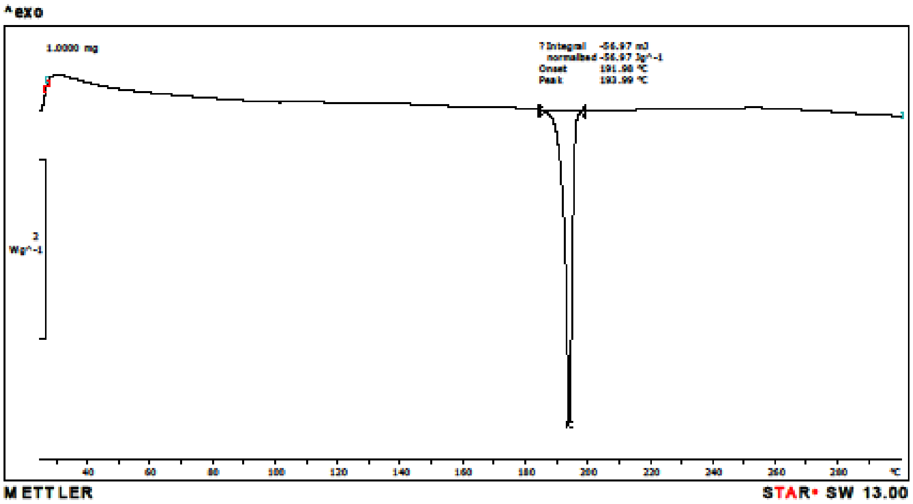

Epimer of JNJ-1802 – ^{1}H NMR

JNJ-1802 Epimer/DMSO

Comment	JNJ-1802 Epimer/DMSO						
File Name						*Owner*	
Frequency (MHz)	400.4032	*Nucleus*	1H	*Solvent*	DMSO-d6	*Number of Transients*	16
Temperature (degree C)	26.960						

^{1}H NMR (400 MHz, DMSO-d_6) δ ppm 3.09 (s, 3 H), 3.73 (s, 3 H), 3.99 (s, 3 H), 6.26 (d, *J*=7.9 Hz, 1 H), 6.57 - 6.61 (m, 2 H), 6.92 (br s, 1 H), 6.98 (dd, *J*=8.1, 2.0 Hz, 1 H), 7.07 (d, *J*=7.9 Hz, 1 H), 7.13 (d, *J*=2.0 Hz, 1 H), 7.22 (dd, *J*=8.8, 1.8 Hz, 1 H), 7.36 (d, *J*=8.4 Hz, 1 H), 7.59 (d, *J*=8.8 Hz, 1 H), 8.07 (d, *J*=0.9 Hz, 1 H), 8.55 (s, 1 H), 12.30 (s, 1 H)

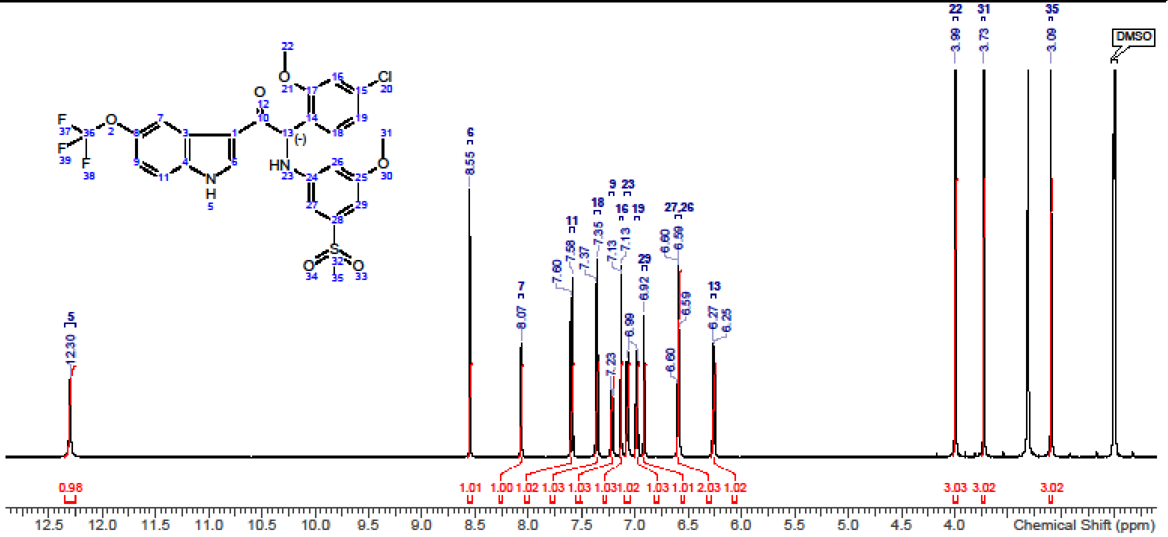

Epimer of JNJ-1802 – ^{13}C NMR

Number of Nuclei	26 C's / 26 C's (spectrum / structure)	Multiplets Integrals Sum	1.00

Formula	$C_{26}H_{22}ClF_3N_2O_6S$	FW	582.9759	$sd_M(^{13}C)$	2.542	$max\_d_M(^{13}C)$	6.226	$d_M(^{13}C)$	1.916

Acquisition Time (sec)	1.3631	Comment				D	0.03	D1	2
DE	6.5	DS	4						
								Frequency (MHz)	100.6807
GB	0	INSTRUM	<spect>	LB	1	NS	2048	Nucleus	13C
Number of Transients	2048	Origin	spect	Original Points Count	32768			PC	1.4
PROBHD	<Z824801_0057 (PA BBO 400S1 BB-H-D-05 Z)>			PULPROG	<zgpg30>	Points Count	32768	Pulse Sequence	zgpg30
Receiver Gain	1620.00	SF	100.680661	SFO1	100.690728146			SI	32768
SSB	0	SW(cyclical) (Hz)	24038.46	SWH	24038.4615384615			Solvent	DMSO-d6
Spectrum Offset (Hz)	10067.1475	Spectrum Type	standard	Sweep Width (Hz)	24037.73	TD	65536	TD0	1
TE	297.9757	Temperature (degree C)	24.976	UNC1	<13C>	WDW	1		

^{13}C NMR (DMSO-d_6, 101 MHz) δ 190.8, 160.9, 157.6, 149.1, 144.5, 142.9, 136.5, 135.5, 133.8, 129.5, 126.6, 126.4, 121.3, 117.3, 120.8, 114.4, 114.3, 113.4, 112.2, 104.5, 103.2, 100.2, 56.8, 55.8, 55.0, 43.9

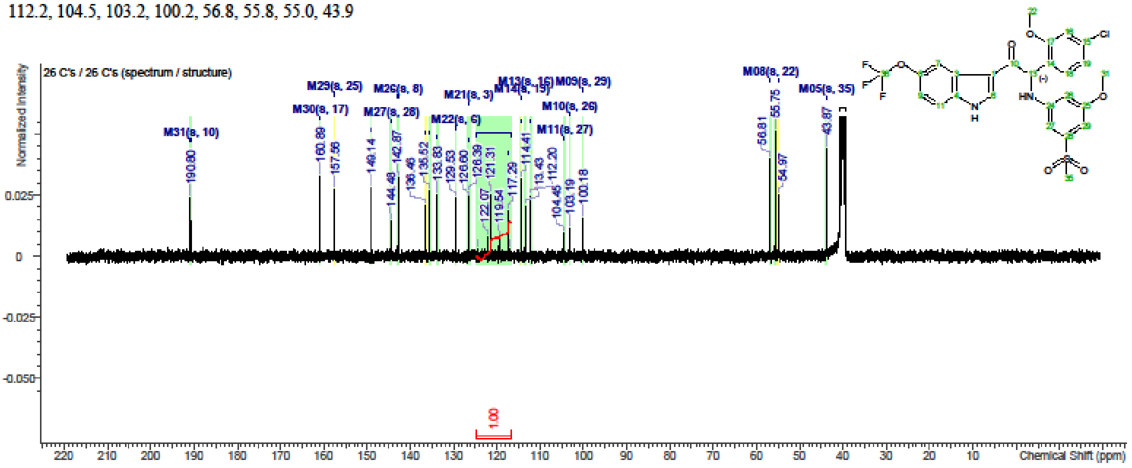

Epimer of JNJ-1802 – LC-MS chromatogram

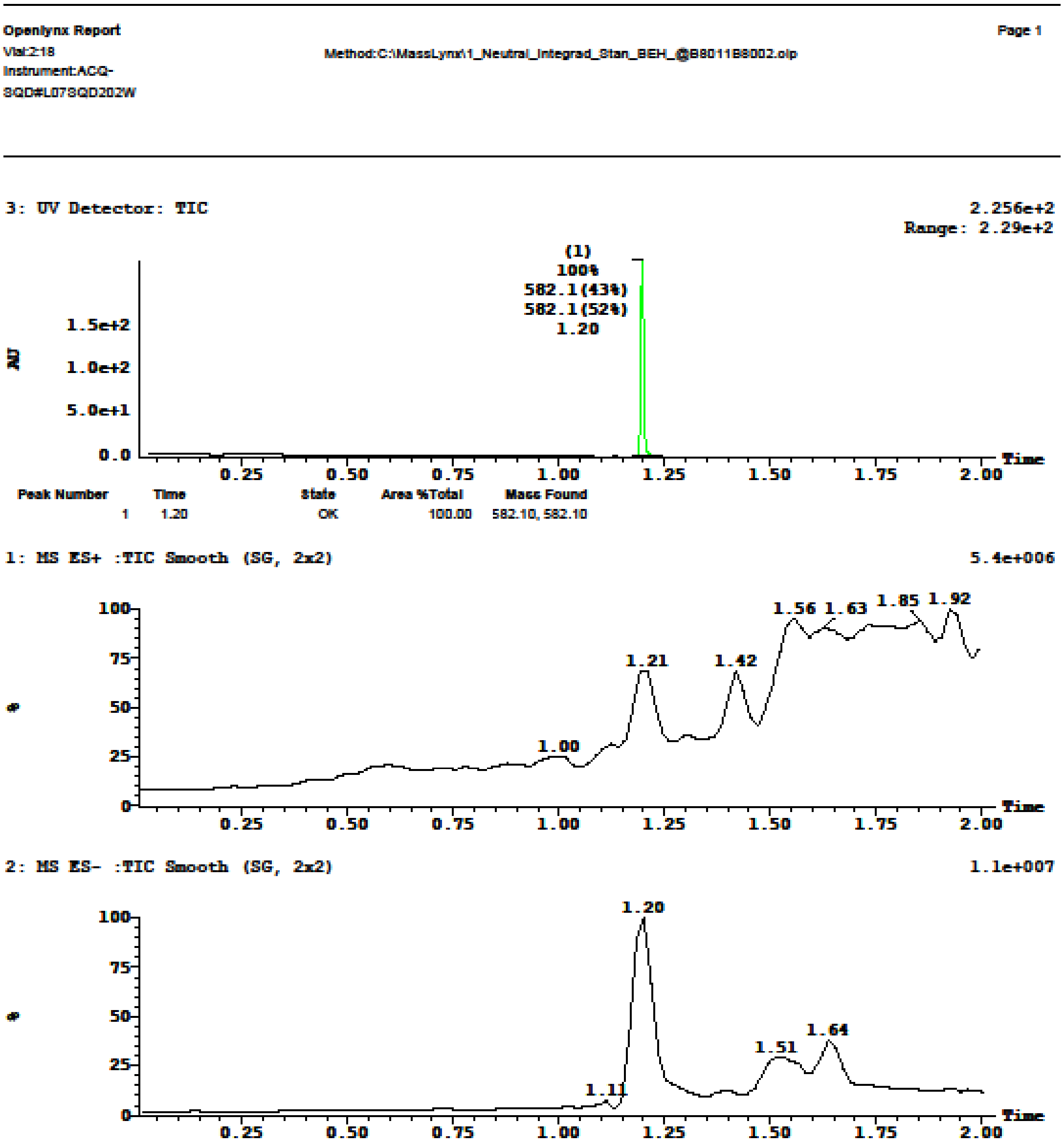

Epimer of JNJ-1802 – DSC trace

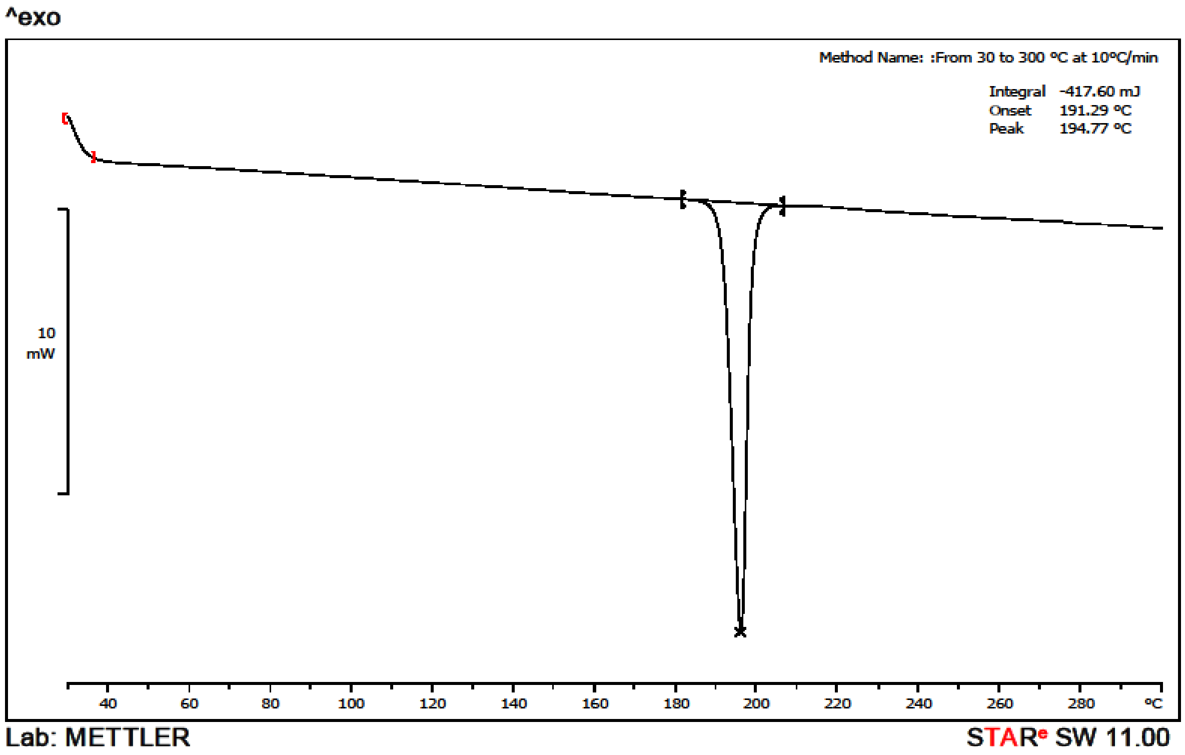

Supplementary Figures

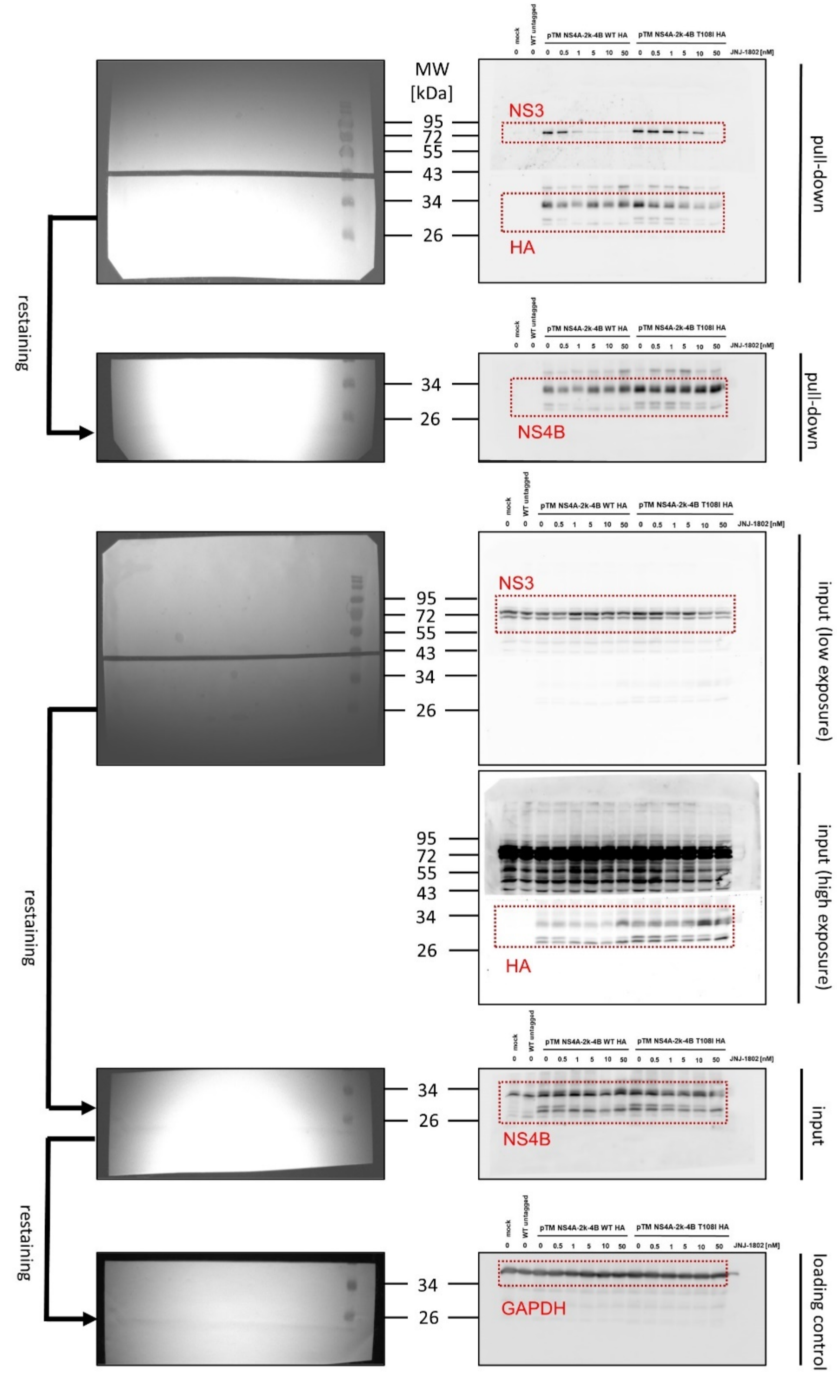

Supplementary Fig. S1. Uncropped western blots from Extended Data Figure 2a

Dose-response assay of JNJ-1802 in cells transfected with pTM NS4A-2K-NS4B (WT and T108I mutant). Eluted samples were loaded onto a gel (see top part) and the membrane was cut

and firstly stained for both NS3 and HA-Tag. Subsequently, the lower part of the membrane was subjected to a stripping protocol (Restore^{TM}PLUS Western Blot Stripping Buffer [ThermoScientific] according to the manufacturer's instructions) and then stained for NS4B. The same procedure was applied to the respective input samples that were loaded onto a separate gel (see middle part). Here, the bottom piece of the membrane was restained again for GAPDH as loading control (see bottom). Red dotted boxes specify areas that were cropped to generate respective panels in Extended Data Figure 2a.

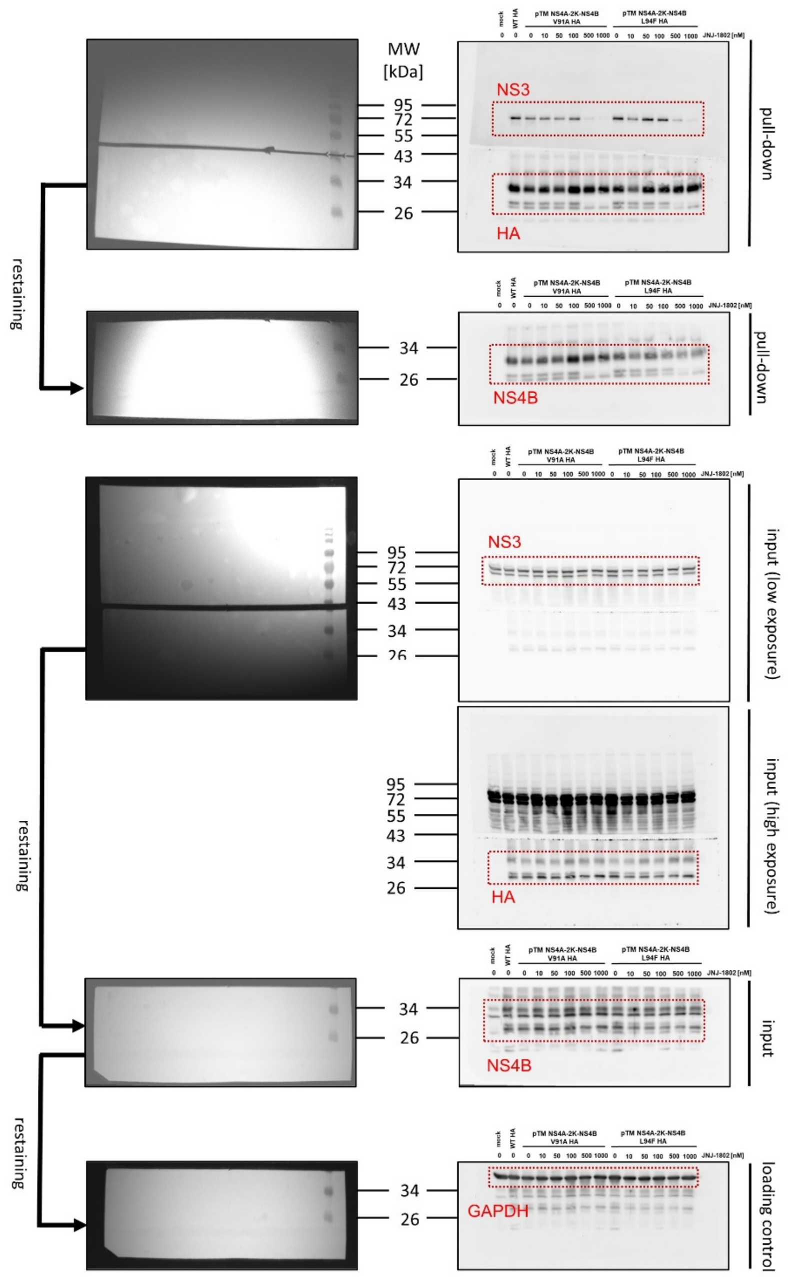

Supplementary Fig. S2. Uncropped western blots from Extended Data Figure 2b

Dose-response assay of JNJ-1802 in cells transfected with pTM NS4A-2K-NS4B (V91A and L94F mutant). Eluted samples were loaded onto a gel (see top part) and the membrane was cut and firstly stained for both NS3 and HA-Tag. Subsequently, the lower part of the membrane was subjected to a stripping protocol (Restore^{TM}PLUS Western Blot Stripping Buffer

[ThermoScientific] according to the manufacturer's instructions) and then stained for NS4B. The same procedure was applied to the respective input samples that were loaded onto a separate gel (see middle part). Here, the bottom piece of the membrane was restained again for GAPDH as loading control (see bottom). Red dotted boxes specify areas that were cropped to generate respective panels in Extended Data Figure 2b.

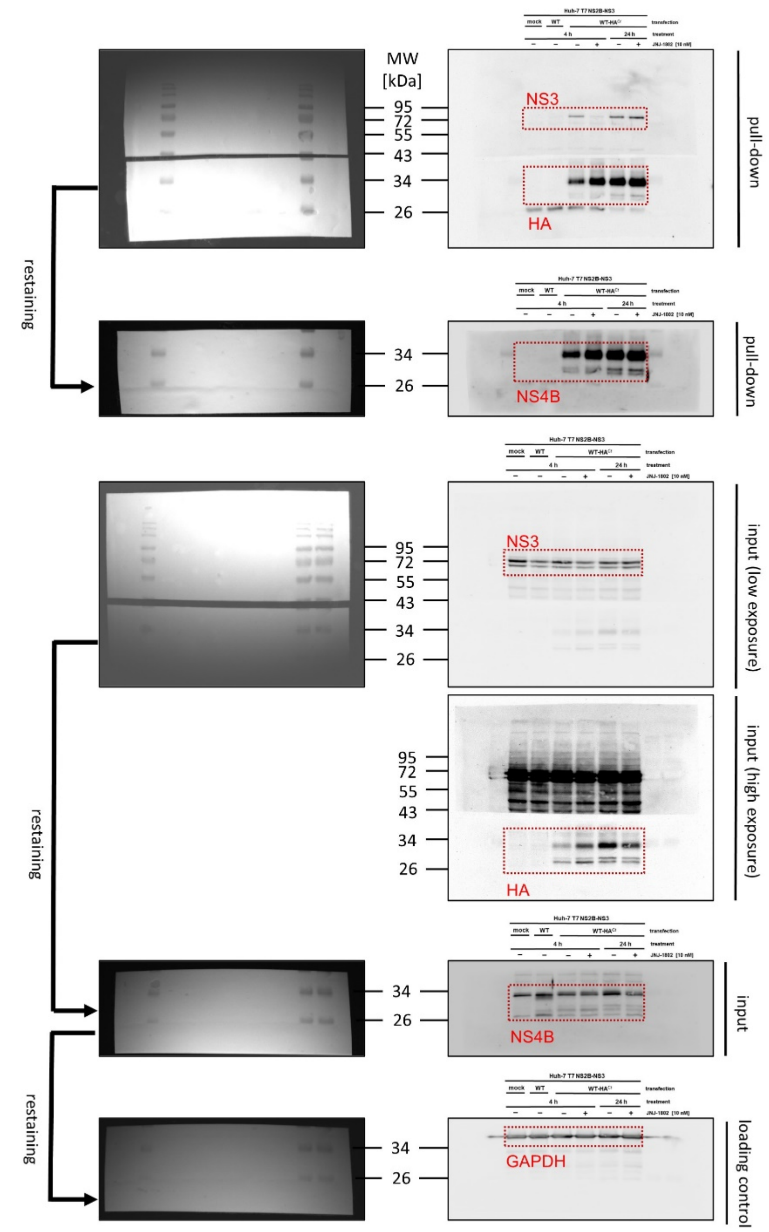

Supplementary Fig. S3. Uncropped western blots from Extended Data Figure 2i

Time-of-addition assay of JNJ-1802 in cells transfected with pTM NS4A-2K-NS4B(-HA^{Ct}). Eluted samples were loaded onto a gel (see top part) and the membrane was cut and firstly stained for both NS3 and HA-Tag. Subsequently, the lower part of the membrane was subjected to a stripping protocol (Restore^{TM}PLUS Western Blot Stripping Buffer [ThermoScientific]

according to the manufacturer's instructions) and then stained for NS4B. The same procedure was applied to the respective input samples that were loaded onto a separate gel (see middle part). Here, the bottom piece of the membrane was restained again for GAPDH as loading control (see bottom). Red dotted boxes specify areas that were cropped to generate respective panels in Extended Data Figure 2i.

Supplementary Tables

Supplementary Table 1. *In vitro* antiviral activity of JNJ-1802 against the different DENV genotypes

Serotype	Genotype	Strain	EC_{50} (nM)	EC_{90} (nM)
DENV-1	G1	Djibouti	0.207 ± 0.056	0.83 ± 0.37
DENV-1	G3	Malaysia°,*	1.34 ± 0.40	3.04 ± 0.69
DENV-1	G4	Indonesia	<0.19[&] ± 0.19	0.80 ± 0.31
DENV-1	G4	45AZ5	1.52 ± 0.18	4.18 ± 0.71
DENV-1	G5	France - Toulon	<0.23[†] ± 0.33	1.02 ± 0.68
DENV-2	Asian America	Martinique*	0.80 ± 0.70	1.88 ± 1.37
DENV-2	American	Trinidad	0.063 ± 0.081	0.162 ± 0.083
DENV-2	Cosmopolitan	France - Toulon	<0.0443[‡] ± 0.0074	0.28[†] ± 0.26
DENV-2	Asian I	Thailand*	1.40 ± 0.34	2.783 ± 0.037
DENV-2	Asian II	Papua New Guinea°	<0.04[ǂ] ± 0.00	0.19 ± 0.10
DENV-2	Sylvatic	Malaysia°	0.0634 ± 0.0022	0.265 ± 0.029
DENV-3	G1	Malaysia	1.28 ± 0.46	3.6 ± 1.9
DENV-3	G2	Thailand	1.68 ± 0.24	5.8 ± 3.0
DENV-3	G3	Bolivia	0.9 ± 1.1	6.71 ± 0.66
DENV-3	G5	Brazil°,#	1.8 ± 1.4	3.8 ± 1.1
DENV-4	G1	India	<0.090[‡] ± 0.090	0.762 ± 0.095
DENV-4	G2a	Malaysia	0.82 ± 0.71	2.1 ± 1.7
DENV-4	G2b	Brazil	0.521 ± 0.080	1.54 ± 0.15
DENV-4	G3	Thailand°	45 ± 12	46 ± 12
DENV-4	Sylvatic	Malaysia°	0.66 ± 0.42	1.6 ± 1.1

°DENV strain that was generated using infectious subgenomic amplicons. *DENV strain that carries the T108I mutation in NS4B. [#]DENV strain that contains the T108A mutation in NS4B. Panel was selected as reported by Touret and colleagues[71].

[&]Two out of four values were below the threshold.

[†]One out of three values was below the threshold.

[‡]Two out of three values were below the threshold.

[ǂ]Two out of two values were below the threshold.

Antiviral assays were carried out in Vero E6 cells. Data represent mean values ± standard deviations from at least two independently performed experiments (*n* = 2 to 6). $EC_{50/90}$, 50%/90% effective concentration.

Supplementary Table *2*. Overview of antiviral activity and cytotoxic assays

Virus	Antiviral activity assay[a]	Cell lines	Cytotoxicity assays[a]	Cell lines
CHIKV (S27)	ATP-based bioluminescent readout after 2 days (ATPLite 1step luminescence assay system; PerkinElmer).	Infected Huh-7 cells	ATP-based bioluminescent readout after 2 days (ATPLite 1step luminescence assay system; PerkinElmer).	Non-infected Huh-7 cells
RSV	Measuring inhibition of eGFP expression of wild-type rgRSV224, a recombinant RSV A2 strain harboring an eGFP reporter gene[69] after 3 days.	Infected HeLa cells	ATP-based bioluminescent readout after 3 days (ATPLite 1step luminescence assay system; PerkinElmer).	Non-infected HeLa cells
INF A/PuertoRico/8/1934 (H1N1)<br>INF A/Taiwan/1/1986 (H1N1)<br>INF B/Singapore/222/1979	ATP-based bioluminescent readout after 3 days (ATPLite 1step luminescence assay system; PerkinElmer).	Infected MDCK cells	ATP-based bioluminescent readout after 3 days (ATPLite 1step luminescence assay system; PerkinElmer).	Non-infected MDCK cells
HCV	Measuring firefly luciferase reporter gene expression after 3 days using the SteadyLite Plus assay kit (PerkinElmer)	Huh-7 cells stably transfected with a selectable self-replicating sub-genomic HCV Genotype 1b (Clone ET) RNA sequence harboring a luciferase reporter gene (Huh-7- Luc cells)[67]	Luciferase activity was quantified after 3 days using the SteadyLite Plus assay kit (PerkinElmer)	Cell lines Huh-7-CMV-Luc (containing an hCMV MIEP Luc construct)
HBV	Intracellular HBV DNA levels were quantified after 3 days using RT-qPCR and fluorescent reporter probes.	Stably transfected cell line, HepG2.117, secreting a high level of hepatitis B virions[72,73]	ATP-based bioluminescent readout after 3 days (ATPLite 1step luminescence assay system; PerkinElmer).	HepG2 cells
ADV	MTS dye reduction assay after 6 days (CellTiter, Promega)	Infected HeLa cells	MTS dye reduction assay after 6 days (CellTiter, Promega)	Non-infected HeLa cells
hCMV	Plaque reduction assay after 6 days	Infected MRC-5 cells	MTS dye reduction assay after 6 days (CellTiter, Promega)	Non-infected MRC-5 cells
HIV-1	Inhibition of β-galactosidase reporter expression	Infected MAGI CCR5 cells containing an HIV 1	MTS dye reduction assay after 2 days (CellTiter, Promega)	Non-infected MAGI CCR5 cells

	using Gal screen reagent (Tropix) according to the manufacturer's instructions after 2 days.	LTR promoter driving expression of the β-galactosidase gene upon HIV 1 Tat transactivation		
HRV-14<br>HRV-16	MTS dye reduction assay after 3 to 4 days (CellTiter, Promega)	Infected HeLa cells	MTS dye reduction assay after 3 to 4 days (CellTiter, Promega)	Non-infected HeLa cells
VACV	Plaque reduction assay after 3 days	Infected Vero cells	MTS dye reduction assay after 3 days (CellTiter, Promega)	Non-infected Vero E6 cells
rVSV	Luciferase activity using BrightGlo reagent (Promega) after 1 day	A549 cells infected with rVSV harboring a luciferase reporter gene	ATP measurement after 1 day using CellTiter-Glo Luminescent Cell Viability Kit (Promega)	Non-infected A549 cells

[a] Assay duration refers to the incubation time with JNJ-1802.

ADV, adenovirus; ATP, adenosine triphosphate; CHIKV, chikungunya virus; eGFP, enhanced green fluorescent protein; HBV, hepatitis B virus; HCV, hepatitis C virus; hCMV, human cytomegalovirus; HIV, human immunodeficiency virus; HRV, human rhinovirus; INF, influenza; MIEP, major immediate-early promoter; MTS, 3 (4,5 dimethylthiazol-2 yl)-5 (3 carboxymethoxyphenyl)-2 (4 sulfophenyl)-2H tetrazolium; RSV, respiratory syncytial virus; RT qPCR, reverse transcription quantitative polymerase chain reaction; rVSV, recombinant vesicular stomatitis virus; VACV, vaccinia virus.

Supplementary Table 3. Overview of the different *in vivo* experiments with JNJ-1802 in mice infected with DENV-2 RL

Dosing regimen	Number of animals	Treatment	Dosage (mg/kg/dose)
**Viraemia model, high viral inoculum (10^6 PFU)**			
b.i.d., 3 days starting from 1 h prior infection	24	Vehicle	0
	8	JNJ-1802	30
	16	JNJ-1802	10
	16	JNJ-1802	3
	16	JNJ-1802	1
	16	JNJ-1802	0.3
	16	JNJ-1802	0.1
**Viraemia model, high viral inoculum (10^6 PFU)**			
q.d., 3 days starting from 1 h prior infection	8	Vehicle	0
	8	JNJ-1802	30
	8	JNJ-1802	3
	8	JNJ-1802	0.3
**Viraemia model, low viral inoculum (10^2 PFU)**			
b.i.d., 6 days starting from 1 h prior infection	16	Vehicle	0
	16	JNJ-1802	10
	16	JNJ-1802	1
	16	JNJ-1802	0.1
**Mortality model, high viral inoculum (10^6 PFU)**			
b.i.d., 5 days starting from 1 h prior infection	10	Vehicle	0
	10	JNJ-1802	10
	10	JNJ-1802	3
	10	JNJ-1802	1
	10	JNJ-1802	0.3
**Delayed treatment (therapeutic setting) (10^2 PFU)**			
b.i.d., 6 days starting on Day 0 p.i.	10	Vehicle (control group)	0
b.i.d., 6 days starting on Day 0 p.i.	10	JNJ-1802 (control group)	30
b.i.d., 6 days starting on Day 4 p.i.	10	JNJ-1802	30
b.i.d., 6 days starting on Day 5 p.i.	10	JNJ-1802	30

DENV, dengue virus; b.i.d., twice daily; PFU, plaque-forming units; p.i., post-infection; q.d., once daily.

Supplementary Table 4: Haematological parameters of vehicle-treated animal

				Dose	0 mg/kg JNJ-1802														
				**Animal code**	R03018			R11106			R12060			R08021			R13060		
				**Day p.i.**	0	7	28	0	7	28	0	7	28	0	7	28	0	7	28
**Haemotological parameters**		**unit**	**Norm min**	**Norm max**															
White blood cell count	WBC	10^9/L	2.06	21.9	8.24	6.79	5.93	7.81	5.77	4.11	10.28	7.35	8.5	13.75	4.75	5.5	11.55	7.55	9.57
Red blood cells count	RBC	10^12/L	4.66	6.14	5.33	4.66	4.95	5.7	4.99	4.51	5.71	4.2	5.28	5.38	4.18	4.36	5.63	4.78	4.92
Haemoglobin	HGB	mmol/L	7.03	8.99	7.8	6.8	7.4	8	7	6.4	8.9	6.5	8.1	8.1	6.4	6.6	8.5	7.3	7.6
Haematocrit	HCT	L/L	0.34	0.42	0.373	0.326	0.352	0.391	0.354	0.326	0.414	0.314	0.389	0.384	0.302	0.318	0.407	0.353	0.372
Mean corpuscular volume	MCV	fL	65.06	75.82	70	70	71.1	68.6	70.9	72.3	72.5	74.8	73.7	71.4	72.2	72.9	72.3	73.8	75.6
Mean corpuscular hemoglobin	MCH	amol	1378	1592	1463	1459	1495	1404	1403	1419	1559	1548	1534	1506	1531	1514	1510	1527	1545
Mean corpuscular hemoglobin conc.	MCHC	mmol/L	20.05	22.13	20.9	20.9	21	20.5	19.8	19.6	21.5	20.7	20.8	21.1	21.2	20.8	20.9	20.7	20.4
Platelet count	PLT	10^9/L	172	493	116	240	109	325	320	400	399	362	434	264	283	352	238	298	291
Red blood cell distribution width - SD	RDW-SD	fL	30.48	38.4	34.3	33.7	34.9	38.5	39.6	38.6	33.4	32.8	34.6	35.4	34	35	37.5	36.8	37.7
Red blood cell distribution - CV	RDW-CV	%	11.9	15.54	13.7	13.3	13.9	16.3	15.4	14.8	12.8	12.3	13.2	14	13.4	13.6	14.2	13.9	14
Platelet distribution width	PDW	fL	9.01	16.57	nd	13.9	nd	14.6	12.1	13.9	9.7	9	9.9	13.8	11.8	12.6	13.8	12.6	14.1
Mean platelet volume	MPV	fL	9.38	13.14	nd	12.2	nd	12.1	10.7	11.9	9.5	9.1	9.7	11.9	11	11.6	11.2	10.8	12.1
Platelet larger cell ratio	P-LCR	%	20.69	49.09	nd	41.5	nd	42.1	32.1	41.2	20	16.5	22.9	40.2	33.3	39.5	34.9	32.2	41.1
Plateletcrit	PCT	%	0.23	0.51	nd	0.29	nd	0.39	0.34	0.48	0.38	0.33	0.42	0.31	0.31	0.41	0.27	0.32	0.35
Neutrophil count	NEUT#	10^9/L	0.91	18.97	5.77	5.62	3.14	6.04	3.69	2.54	8.3	5.14	5.3	10.09	2.33	2.04	9.42	4.1	4.44
Lymphocyte count	LYMPH#	10^9/L	0.26	4.18	1.98	0.93	2.33	1.28	1.62	1.26	1.35	1.64	2.5	2.4	1.82	2.82	1.49	2.41	3.96
Monocyte count	MONO#	10^9/L	0	1.16	0.49	0.23	0.38	0.48	0.44	0.31	0.63	0.44	0.62	1.12	0.48	0.47	0.53	0.69	0.87
Eosinophil count	EO#	10^9/L	0	0.43	0	0	0.07	0	0.02	0	0	0.12	0.07	0.13	0.12	0.16	0.11	0.35	0.29
Basophil count	BASO#	10^9/L	0	0.03	0	0.01	0.01	0.01	0	0	0	0.01	0.01	0.01	0	0.01	0	0	0.01
% Neutrophil	NEUT%	%	42.64	100	70.1	82.8	52.9	77.4	64	61.8	80.8	70	62.4	73.4	49.1	37.1	81.5	54.4	46.4
% Lymphocyte	LYMPH%	%	1.4	48.02	24	13.7	39.3	16.4	28.1	30.7	13.1	22.3	29.4	17.5	38.3	51.3	12.9	31.9	41.4
% Monocyte	MONO%	%	1.86	9.06	5.9	3.4	6.4	6.1	7.6	7.5	6.1	6	7.3	8.1	10.1	8.5	4.6	9.1	9.1
% Eosinophil	EO%	%	0	4.59	0	0	1.2	0	0.3	0	0	1.6	0.8	0.9	2.5	2.9	1	4.6	3
% Basophil	BASO%	%	0	0.27	0	0.1	0.2	0.1	0	0	0	0	0.1	0.1	0	0.2	0	0	0.1

Values which are outside the normal minimum and maximum values are highlighted in orange. CV, coefficient of variation; n.d., not determined; p.i.; post-infection; SD, standard deviation.

Supplementary Table 5: Haematological parameters of animals treated with 0.01 mg/kg/day JNJ-1802

				Dose	0.01 mg/kg JNJ-1802								
				**Animal code**	R04051			R01039			R12088		
				**Day p.i.**	0	7	28	0	7	28	0	7	28
**Haematological parameters**		**unit**	**Norm min**	**Norm max**									
White blood cell count	WBC	10^9/L	2.06	21.9	8.91	3.59	5.65	9.98	8.74	11.83	8.06	7.16	4.81
Red blood cells count	RBC	10^12/L	4.66	6.14	5.83	5.07	5.21	6.47	5.54	5.34	5.26	4.87	5.29
Haemoglobin	HGB	mmol/L	7.03	8.99	8.1	7.1	7.4	9.3	8.1	7.6	7.9	7.4	7.9
Haematocrit	HCT	L/L	0.34	0.42	0.39	0.342	0.36	0.443	0.37	0.371	0.382	0.358	0.384
Mean corpuscular volume	MCV	fL	65.06	75.82	66.9	67.5	69.1	68.5	66.8	69.5	72.6	73.5	72.6
Mean corpuscular hemoglobin	MCH	amol	1378	1592	1389	1400	1420	1437	1462	1423	1502	1520	1493
Mean corpuscular hemoglobin conc.	MCHC	mmol/L	20.05	22.13	20.8	20.8	20.6	21	21.9	20.5	20.7	20.7	20.6
Platelet count	PLT	10^9/L	172	493	311	382	405	311	319	439	288	314	359
Red blood cell distribution width - SD	RDW-SD	fL	30.48	38.4	36.2	36.2	36.9	34.9	32.3	35.2	35.7	36.5	36.4
Red blood cell distribution - CV	RDW-CV	%	11.9	15.54	15.3	14.9	14.8	15.4	13.5	14.1	13.6	13.7	13.7
Platelet distribution width	PDW	fL	9.01	16.57	11.9	10.1	11	11.7	9.7	10.6	10.1	9.9	10.4
Mean platelet volume	MPV	fL	9.38	13.14	10.8	9.5	10.5	10.5	9.2	9.8	9.6	9.3	9.8
Platelet larger cell ratio	P-LCR	%	20.69	49.09	31.5	21.3	29.7	28.8	18.9	23.9	21.9	19.8	23.5
Plateletcrit	PCT	%	0.23	0.51	0.33	0.36	0.43	0.33	0.29	0.43	0.28	0.29	0.35
Neutrophil count	NEUT#	10^9/L	0.91	18.97	6.42	2.08	2.82	9.13	7.28	9.5	6.07	4.83	2.79
Lymphocyte count	LYMPH#	10^9/L	0.26	4.18	2.12	1.26	2.55	0.51	0.91	1.5	1.48	1.74	1.65
Monocyte count	MONO#	10^9/L	0	1.16	0.37	0.25	0.27	0.34	0.47	0.71	0.51	0.59	0.36
Eosinophil count	EO#	10^9/L	0	0.43	0	0	0	0	0.08	0.11	0	0	0
Basophil count	BASO#	10^9/L	0	0.03	0	0	0.01	0	0	0.01	0	0	0.01
% Neutrophil	NEUT%	%	42.64	100	72	57.9	49.9	91.5	83.3	80.3	75.3	67.5	58
% Lymphocyte	LYMPH%	%	1.4	48.02	23.8	35.1	45.1	5.1	10.4	12.7	18.4	24.3	34.3
% Monocyte	MONO%	%	1.86	9.06	4.2	7	4.8	3.4	5.4	6	6.3	8.2	7.5
% Eosinophil	EO%	%	0	4.59	0	0	0	0	0.9	0.9	0	0	0
% Basophil	BASO%	%	0	0.27	0	0	0.2	0	0	0.1	0	0	0.2

Values which are outside the normal minimum and maximum values are highlighted in orange. CV, coefficient of variation; p.i., post-infection; SD, standard deviation.

Supplementary Table 6: Haematological parameters of animals treated with 0.18 mg/kg/day JNJ-1802

				Dose	0.18 mg/kg JNJ-1802								
				**Animal code**	R09066			R10069			R12109		
				**Day p.i.**	0	7	28	0	7	28	0	7	28
**Haematological parameters**		**unit**	**Norm min**	**Norm max**									
White blood cell count	WBC	10^9/L	2.06	21.9	8.62	7.85	5.58	13.23	9.94	5.23	7.31	9.57	5.42
Red blood cells count	RBC	10^12/L	4.66	6.14	5.59	5.02	5.09	5.6	4.62	4.78	5.61	5.29	5.77
Haemoglobin	HGB	mmol/L	7.03	8.99	8.4	7.7	7.9	8	6.8	7.2	8.2	7.9	8.5
Haematocrit	HCT	L/L	0.34	0.42	0.406	0.368	0.377	0.391	0.325	0.339	0.399	0.379	0.411
Mean corpuscular volume	MCV	fL	65.06	75.82	72.6	73.3	74.1	69.8	70.3	70.9	71.1	71.6	71.2
Mean corpuscular hemoglobin	MCH	amol	1378	1592	1503	1534	1552	1429	1472	1506	1462	1493	1473
Mean corpuscular hemoglobin conc.	MCHC	mmol/L	20.05	22.13	20.7	20.9	21	20.5	20.9	21.2	20.6	20.8	20.7
Platelet count	PLT	10^9/L	172	493	328	280	380	310	300	330	367	363	286
Red blood cell distribution width - SD	RDW-SD	fL	30.48	38.4	35.9	35.2	35.8	34.5	33.2	33.6	35	33.8	33.9
Red blood cell distribution - CV	RDW-CV	%	11.9	15.54	13.5	13.4	13.7	13.6	13.2	13.2	13.6	13.2	13.3
Platelet distribution width	PDW	fL	9.01	16.57	10.5	11.5	11.6	11.8	10.6	11.9	12.8	13.1	15.1
Mean platelet volume	MPV	fL	9.38	13.14	9.9	10.5	10.3	11.1	10.4	11.2	11.7	11.6	12.9
Platelet larger cell ratio	P-LCR	%	20.69	49.09	23.9	28.1	28.5	34.9	27.3	34.3	39.1	37	46.2
Plateletcrit	PCT	%	0.23	0.51	0.32	0.29	0.39	0.34	0.31	0.37	0.43	0.42	0.37
Neutrophil count	NEUT#	10^9/L	0.91	18.97	6.2	5.72	3.48	11.04	8.03	3.16	5.09	6.2	2.15
Lymphocyte count	LYMPH#	10^9/L	0.26	4.18	1.73	1.54	1.57	1.6	1.43	1.81	1.93	3.03	2.93
Monocyte count	MONO#	10^9/L	0	1.16	0.68	0.36	0.41	0.59	0.44	0.26	0.29	0.32	0.32
Eosinophil count	EO#	10^9/L	0	0.43	0.01	0.23	0.12	0	0.04	0	0	0.01	0
Basophil count	BASO#	10^9/L	0	0.03	0	0	0	0	0	0	0	0.01	0.02
% Neutrophil	NEUT%	%	42.64	100	71.9	72.9	62.4	83.4	80.8	60.4	69.6	64.8	39.6
% Lymphocyte	LYMPH%	%	1.4	48.02	20.1	19.6	28.1	12.1	14.4	34.6	26.4	31.7	54.1
% Monocyte	MONO%	%	1.86	9.06	7.9	4.6	7.3	4.5	4.4	5	4	3.3	5.9
% Eosinophil	EO%	%	0	4.59	0.1	2.9	2.2	0	0.4	0	0	0.1	0
% Basophil	BASO%	%	0	0.27	0	0	0	0	0	0	0	0.1	0.4

Values which are outside the normal minimum and maximum values are highlighted in orange. CV, coefficient of variation; p.i., post-infection; SD, standard deviation.

Supplementary Table 7: Haematological parameters of animals treated with 3 mg/kg/day

				**Dose**	3 mg/kg								
				**Animal code**	R09041			R10035			R13148		
				**Day p.i.**	0	7	28	0	7	28	0	7	28
**Haematological parameters**		**unit**	**Norm min**	**Norm max**									
White blood cell count	WBC	10^9/L	2.06	21.9	10.7	6.22	9.86	9.07	9.31	12.34	8.67	8.49	8.06
Red blood cells count	RBC	10^12/L	4.66	6.14	4.94	3.83	4.46	6.53	5.55	6.26	6.14	5.13	5.12
Haemoglobin	HGB	mmol/L	7.03	8.99	6.9	5.3	5.6	9.3	8	9	8.7	7.3	7.4
Haematocrit	HCT	L/L	0.34	0.42	0.343	0.273	0.296	0.442	0.38	0.441	0.415	0.361	0.362
Mean corpuscular volume	MCV	fL	65.06	75.82	69.4	71.3	66.4	67.7	68.5	70.4	67.6	70.4	70.7
Mean corpuscular hemoglobin	MCH	amol	1378	1592	1397	1384	1256	1424	1441	1438	1417	1423	1445
Mean corpuscular hemoglobin conc.	MCHC	mmol/L	20.05	22.13	20.1	19.4	18.9	21	21.1	20.4	21	20.2	20.4
Platelet count	PLT	10^9/L	172	493	285	284	322	273	240	276	241	298	257
Red blood cell distribution width - SD	RDW-SD	fL	30.48	38.4	37.7	36.9	36.1	36.4	36.3	38.6	38.7	39.5	38.1
Red blood cell distribution - CV	RDW-CV	%	11.9	15.54	15.2	14.8	15.4	16.2	14.6	16.4	17.2	15.5	15.1
Platelet distribution width	PDW	fL	9.01	16.57	13.8	12.5	14.3	14.1	12.9	14.2	17.6	13.2	16.3
Mean platelet volume	MPV	fL	9.38	13.14	12.4	11.5	12.1	11.4	10.8	11.9	13.1	11.6	13.3
Platelet larger cell ratio	P-LCR	%	20.69	49.09	44.9	35.7	43.5	37.4	32.9	40.8	50.4	40.5	52.9
Plateletcrit	PCT	%	0.23	0.51	0.35	0.33	0.39	0.31	0.26	0.33	0.32	0.35	0.34
Neutrophil count	NEUT#	10^9/L	0.91	18.97	9.41	4.76	7.72	5.7	7.1	7.59	5.86	4.84	4.5
Lymphocyte count	LYMPH#	10^9/L	0.26	4.18	0.65	0.89	1.33	2.21	1.72	3.28	2.15	3.08	2.91
Monocyte count	MONO#	10^9/L	0	1.16	0.64	0.57	0.8	1.16	0.43	1.42	0.65	0.52	0.61
Eosinophil count	EO#	10^9/L	0	0.43	0	0	0	0	0.04	0	0.01	0.05	0.04
Basophil count	BASO#	10^9/L	0	0.03	0	0	0.01	0	0.02	0.05	0	0	0
% Neutrophil	NEUT%	%	42.64	100	87.9	76.5	78.3	62.8	76.3	61.5	67.6	57	55.8
% Lymphocyte	LYMPH%	%	1.4	48.02	6.1	14.3	13.5	24.4	18.5	26.6	24.8	36.3	36.1
% Monocyte	MONO%	%	1.86	9.06	6	9.2	8.1	12.8	4.6	11.5	7.5	6.1	7.6
% Eosinophil	EO%	%	0	4.59	0	0	0	0	0.4	0	0.1	0.6	0.5
% Basophil	BASO%	%	0	0.27	0	0	0	0	0.2	0.4	0	0	0

Values which are outside the normal minimum and maximum values are highlighted in orange. CV, coefficient of variation; p.i., post-infection; SD, standard deviation.

References

66 Sun, D. & Nassal, M. Stable HepG2- and Huh7-based human hepatoma cell lines for efficient regulated expression of infectious hepatitis B virus. *J. Hepatol.* **45**, 636-645, doi:10.1016/j.jhep.2006.05.019 (2006).

67 Lohmann, V. *et al.* Replication of subgenomic hepatitis C virus RNAs in a hepatoma cell line. *Science* **285**, 110-113, doi:10.1126/science.285.5424.110 (1999).

68 Nakabayashi, H., Taketa, K., Miyano, K., Yamane, T. & Sato, J. Growth of human hepatoma cells lines with differentiated functions in chemically defined medium. *Cancer Res.* **42**, 3858-3863 (1982).

69 Hallak, L. K., Spillmann, D., Collins, P. L. & Peeples, M. E. Glycosaminoglycan sulfation requirements for respiratory syncytial virus infection. *J. Virol.* **74**, 10508-10513, doi:10.1128/jvi.74.22.10508-10513.2000 (2000).

70 Kaptein, S. J. F. *et al.* A pan-serotype dengue virus inhibitor targeting the NS3-NS4B interaction. *Nature* **598**, 504-509, doi:10.1038/s41586-021-03990-6 (2021).

71 Touret, F. *et al.* Phylogenetically based establishment of a dengue virus panel, representing all available genotypes, as a tool in dengue drug discovery. *Antiviral Res.* **168**, 109-113, doi:10.1016/j.antiviral.2019.05.005 (2019).

72 Korba, B. E. & Gerin, J. L. Use of a standardized cell culture assay to assess activities of nucleoside analogs against hepatitis B virus replication. *Antiviral Res.* **19**, 55-70, doi:10.1016/0166-3542(92)90056-b (1992).

73 Sells, M. A., Chen, M. L. & Acs, G. Production of hepatitis B virus particles in Hep G2 cells transfected with cloned hepatitis B virus DNA. *Proc. Natl. Acad. Sci. U. S. A.* **84**, 1005-1009, doi:10.1073/pnas.84.4.1005 (1987).
