# Supplementary material for: Blocking NS3–NS4B interaction inhibits dengue virus in non-human primates
Source: Nature. 2023 Mar 15;615(7953):678–86. doi: 10.1038/s41586-023-05790-6 (PMC10033419; doi:10.1038/s41586-023-05790-6)
Supplement: Supplementary file 2 — Reporting Summary [file 41586_2023_5790_MOESM2_ESM.pdf]

nature portfolio

Corresponding author(s): Marnix Van Loock

Last updated by author(s): Jan 27, 2023

Reporting Summary

Nature Portfolio wishes to improve the reproducibility of the work that we publish. This form provides structure for consistency and transparency in reporting. For further information on Nature Portfolio policies, see our Editorial Policies and the Editorial Policy Checklist.

Statistics

For all statistical analyses, confirm that the following items are present in the figure legend, table legend, main text, or Methods section.

n/a	Confirmed	
☐	☒	The exact sample size (*n*) for each experimental group/condition, given as a discrete number and unit of measurement
☐	☒	A statement on whether measurements were taken from distinct samples or whether the same sample was measured repeatedly
☐	☒	The statistical test(s) used AND whether they are one- or two-sided<br>*Only common tests should be described solely by name; describe more complex techniques in the Methods section.*
☒	☐	A description of all covariates tested
☐	☒	A description of any assumptions or corrections, such as tests of normality and adjustment for multiple comparisons
☐	☒	A full description of the statistical parameters including central tendency (e.g. means) or other basic estimates (e.g. regression coefficient) AND variation (e.g. standard deviation) or associated estimates of uncertainty (e.g. confidence intervals)
☐	☒	For null hypothesis testing, the test statistic (e.g. *F*, *t*, *r*) with confidence intervals, effect sizes, degrees of freedom and *P* value noted<br>*Give P values as exact values whenever suitable.*
☐	☒	For Bayesian analysis, information on the choice of priors and Markov chain Monte Carlo settings
☒	☐	For hierarchical and complex designs, identification of the appropriate level for tests and full reporting of outcomes
☒	☐	Estimates of effect sizes (e.g. Cohen's *d*, Pearson's *r*), indicating how they were calculated

Our web collection on statistics for biologists contains articles on many of the points above.

Software and code

Policy information about availability of computer code

Data collection	Automated RNA extractions were performed on the QIAcube HT automat (Qiagen). For RT-qPCR, a Bio-Rad CFX Connect real-time system or an ABI 7500DX Fast Real Time PCR system was used. Whole genome sequencing was performed on a MiSeq platform (Illumina). Luminescence was measured using a Viewlux (PerkinElmer) apparatus, Microbeta Trilux luminescence reader (PerkinElmer, Wallac) or Envision plate reader (PerkinElmer). Spectrophotometrical measurements were performed using a Molecular Devices SpectraMax plate reader, a Molecular Devices Vmax plate reader or a Bio-rad iMark microplate reader. Western blots data were collected using a chemoluminescence imager (ECL ChemoCamImager, Intas Science Imaging Instruments GmbH). Compound concentrations in the plasma samples were determined using an API 5500 LC–MS/MS system mass spectrometer (Applied Biosystems).
Data analysis	RT-qPCR data were analysed using the QuantStudio 12K Flex software (v1.2.3) or SDS 1.2 Applied Biosystems software. Inhibition values for antiviral molecules were plotted using KaleidaGraph plotting software (version 4.03, Synergy Software). The NS4B protein sequence of various flaviviruses was aligned using Clustal Omega Version 2.1. Western blot data were quantified using ImageJ (version 2.1.0/1.53j; Wayne Rasband and contributors, National Institutes of Health, USA). GraphPad Prism Version v9.0.0 & v7.04 (GraphPad Software, Inc.) or R software (version 3.4.2) was used for statistical evaluations. Individual plasma concentration-time profiles were subjected to a non-compartmental pharmacokinetics analysis using Phoenix WinNonlin v.6.3. (Certara). A custom script (PMID 25178459) was used to derive the amino acid composition of each sample for all coding sequences per DENV genotype, which was not specifically developed for this research but for all similar analyses. The code for the custom script is deposited as part of the pipeline VirVarSeq but is individually accessible on the Open Source software platform SourceForge (https://sourceforge.net/projects/virtools/?source=directory). The code for this specific variant detection script is 'codon_table.pl'.Graphs and figures were generated using Microsoft PowerPoint (Version 2208 Build 16.0.15601.20280), GraphPad Prism (v9.0.0 & v7.04) or Adobe Illustrator (2020 24.0.2); the software is made available by Janssen Pharmaceutical NV.

For manuscripts utilizing custom algorithms or software that are central to the research but not yet described in published literature, software must be made available to editors and reviewers. We strongly encourage code deposition in a community repository (e.g. GitHub). See the Nature Portfolio guidelines for submitting code & software for further information.

Data

Policy information about availability of data

All manuscripts must include a data availability statement. This statement should provide the following information, where applicable:

- Accession codes, unique identifiers, or web links for publicly available datasets
- A description of any restrictions on data availability
- For clinical datasets or third party data, please ensure that the statement adheres to our policy

All data supporting the findings of this study are available within the article and all accession codes are provided in the manuscript.
The genome sequence of DENV-2 RL is deposited at GenBank (accession MW741553). The synthesis and chemical characterization of all compounds described in this paper is provided as Supplementary Information (Supplementary Methods). The uncropped images of the western blots shown in Extended Data Figs. 2 a,b and i are presented in Supplementary Figs. 1–3. All data supporting the findings of this study are available within the article or the Supplementary Information.
Code availability
A custom script (PMID 25178459) was used to derive the amino acid composition of each sample for all coding sequences per DENV genotype, which was not specifically developed for this research but for all similar analyses. The code for the custom script is deposited as part of the pipeline VirVarSeq but is individually accessible on the Open Source software platform SourceForge (https://sourceforge.net/projects/virtools/?source=directory). The code for this specific variant detection script is 'codon_table.pl'. The natural occurrence of the mutations was retrieved from the Virus Pathogen Resource database (www.viprbrc.org; accessed in May 2020).Graphs and figures were generated using Microsoft PowerPoint (Version 2208 Build 16.0.15601.20280), GraphPad Prism (v9.0.0 & v7.04) or Adobe Illustrator (2020 24.0.2); the software is made available by Janssen Pharmaceutical NV. In some figures, basic templates obtained from the Servier Medical Art library (https://smart.servier.com/) were used.

Human research participants

Policy information about studies involving human research participants and Sex and Gender in Research.

Reporting on sex and gender	N/A
Population characteristics	N/A
Recruitment	N/A
Ethics oversight	N/A

Note that full information on the approval of the study protocol must also be provided in the manuscript.

Field-specific reporting

Please select the one below that is the best fit for your research. If you are not sure, read the appropriate sections before making your selection.

☒ Life sciences ☐ Behavioural & social sciences ☐ Ecological, evolutionary & environmental sciences

For a reference copy of the document with all sections, see nature.com/documents/nr-reporting-summary-flat.pdf

Life sciences study design

All studies must disclose on these points even when the disclosure is negative.

Sample size

For almost all in vitro studies, three or more independent experiments (either in duplicate or triplicate) were performed. Resistant selection experiments with the compound were obtained in two independent efforts (A and B sample; Ext. Data Fig. 1a, b). For the EC50 dose response experiments, highly validated assays were used with at least two or more experiments. As we saw consistent results in the past using this number of experiments and the results received from our reference compounds that were taken along were as expected, we consider two or more independent experiments as sufficient. Three independent experiments were performed for the Western Blots which gave consistent results. These experiments are very labour-intensive and we deem the sample size of three sufficient given the high reproducibility of the results.
Statistical power calculations considered the number of mice required to detect a significant reduction in viraemia compared with vehicle-treated controls. With groups of $n = 8$, a reduction of at least 0.8 log10 in viral RNA can be detected according to the independent t-test (with $\alpha = 0.05$, power = 80% and a standard deviation value of 0.5). In addition, statistical calculations considered the number of mice required to detect a significant improvement in survival compared with vehicle-treated controls. With groups of $n = 11$, a minimal survival rate of 60% for treated mice versus 0% in the untreated, infected control group can be demonstrated according to the Fisher's exact test (with $\alpha = 0.05$ and power = 80%).
To assess the in vivo efficacy of JNJ-1802 against DENV-2 in NHPs, no formal sample size calculation was performed. To assess antiviral activity of JNJ-1802 in rhesus macaques infected with the DENV-1/45AZ5 virus strain, sample size was calculated using a one-sided Fisher exact test and an $\alpha = 0.05$ targeting a power of at least 80%. This sample size is sufficient to detect a significant difference in the number of infected animals in each of the groups as low as 83%.

Data exclusions

For the antiviral assays performed at Aix-Marseille Université (pan-genotype and pan-serotype testing), the following inclusion/exclusion

Data exclusions	criteria were used:<br>To be validated, an experiment needs to pass some inclusion criteria regarding the standard deviation (StDEV) of the Ct values (RT-qPCR):<br>- StDEV of Ct of the virus controls (VCs) should be <0.5, and<br>- If StDEV of triplicates is <1 for all compound dilutions, use all inhibition values for generating the dose-response curve<br>- If StDEV of triplicates is <1 for at least four compound dilutions, including the first inhibition value below 50%, then use those inhibition values for generating the dose-response curve.<br>When the Ct's StDEV of the VCs are >0.5 or when the Ct values of the inhibition triplicates do not pass the inclusion criteria, the inhibition values are excluded, and the experiment was repeated.<br>Some data points from the animal experiments were excluded because of deviating RT-qPCR curves.<br>No data were excluded from other experiments.
Replication	Three or more independent experiments (either in duplicate or triplicate) were performed for almost all in vitro experiments and at least two independent experiments for almost all in vivo studies. All attempts at replication were consistent and reflect the intra and inter variability. The antiviral effect of JNJ-1802 was assessed independently in different laboratories (KU Leuven, Janssen Pharmaceutica, Aix-Marseille Université and Heidelberg University), which produced comparable results.
Randomization	Allocation of mice or monkeys to experimental groups was performed randomly. For the in vitro experiments performed in this study, randomization was not relevant as no allocation to experimental treatment groups is required. Reference compounds and proper controls were taken along to assess consistency over time. We received consistent results over time with repeats performed on different days and by different people.
Blinding	As for the animal studies, samples (blood) obtained from mice were collected in tubes labelled from 1 to x. Typically, these samples were next processed by technicians not involved in the treatment/manipulation of the mice. The DENV-2 NHP studies were not blinded to the animal caretakers who performed all animal handling and blood collections. However, these individuals were not involved in the processing of the collected materials. The latter was done by laboratory technicians who received the blood samples in coded tubes for further analysis. For DENV-3 NHP studies, the veterinary staff were not told which material (drug/placebo) they were delivering to a specific animal. Sample processing and conducting of assays was performed by laboratory technicians not involved in the treatment/manipulation of the animals. Blood samples were labelled with animal ID numbers and processed in a non-specific manner simultaneously (e.g., separation of sera from whole blood into aliquots labelled only with the date, study time point, animal ID number, and study ID number); knowledge of treatment group is not relevant to this task since all samples are processed identically and no interpretation is required. All assays were performed on samples of all animals by laboratory technicians; the samples and raw data were not labelled with their treatment group.<br>For the in vitro experiments performed in the study, blinding was not applicable as no experimental treatment groups were used where the quality of the outcome could be influenced .

Reporting for specific materials, systems and methods

We require information from authors about some types of materials, experimental systems and methods used in many studies. Here, indicate whether each material, system or method listed is relevant to your study. If you are not sure if a list item applies to your research, read the appropriate section before selecting a response.

Materials & experimental systems

n/a	Involved in the study	
☐	☒	Antibodies
☐	☒	Eukaryotic cell lines
☒	☐	Palaeontology and archaeology
☐	☒	Animals and other organisms
☒	☐	Clinical data
☒	☐	Dual use research of concern

Methods

n/a	Involved in the study	
☒	☐	ChIP-seq
☒	☐	Flow cytometry
☒	☐	MRI-based neuroimaging

Antibodies

Antibodies used	Mouse monoclonal anti-GAPDH, G-9 (sc-365062, Lot # I2320, Santa Cruz Biotechnology, 1:1000 dilution). Mouse monoclonal anti-β-actin, clone AC-15 (A5441, Lot # 079M4799V, Sigma-Aldrich, dilution 1:5000). Mouse monoclonal anti-HA agarose beads, clone HA-7 (A2095, Lot # 119M4756V, Sigma-Aldrich). Mouse anti-HA.11 epitope tag, clone: 16B12 (901502, Lot #: B276381, BioLegend, dilution 1:1000). Rabbit polyclonal anti-NS3 (1:2000 dilution) or anti-NS4B (1:1000 dilution) antibodies were generated in-house (Miller et al. 2006, PMID 1643638). Anti-flavivirus group monoclonal antigen antibody, clone D1-4G2-4-15 (MAB10216, Lot # 2441960, Millipore/ Merck, dilution 1:50 for survival study). KPL Peroxidase-labelled goat anti-monkey IgM (Sero Care Cat. # 074-11-031), or IgG (Sigma Cat. # SAB3700766).
Validation	Almost all antibodies were obtained from a commercial sources and have often been referred to by us and others.<br>sc-365062: same used in PMID 34616043<br>A5441: same used in PMID 34616043<br>A2095: same used in PMID 34616043<br>16B12: same used in PMID 35216024<br>MAB10216: same used in PMID 34616043<br>074-11-031: same used in PMID 6172519<br>The generation of the polyclonal antibodies against NS3 and the polyclonal antibodies against NS4B was reported in PMID 16436383.

Eukaryotic cell lines

Policy information about cell lines and Sex and Gender in Research

Cell line source(s)	Monkey African Green kidney cells (Vero; ECACC CL 84113001/ATCC CCL 81; Vero E6: ATCC CRL-1586), C6/36 mosquito cells (from Aedes albopictus; ATCC CCL-1660). THP-1/DC-SIGN THP-1 cells (TIB-202, ATCC) Madin-Darby canine kidney (MDCK) were obtained from ATCC. HeLa cell lines (cervical epithelial, human) were obtained from ATCC. The hepatoblastoma cell line HepG2 was obtained from ATCC. HepG2.117 cells (Sun and Nassal., J Hepatol. 2006) were kindly provided by Prof. M. Nassal, University Hospital Freiburg, Freiburg, Germany. Human hepatocellular carcinoma cells (Huh-7; Nakabayashi et al., Cancer Research 1982) were obtained from Prof. Heinz Schaller (Center for Molecular Biology Heidelberg (ZMBH), Germany). MT4-LTR-Luc cells were generated in-house. MRC-5 cells (secondary human fetal lung fibroblast; ATCC) MAGI-CCR5 cells were obtained from the National Institutes of Health (NIH; Bethesda, MA) acquired immune deficiency syndrome (AIDS) Research and Reference Reagent Program. Human lung epithelial A549 cells were obtained from ATCC. Huh-7-Luc cells (Huh-7 human hepatoma cells that are stably transfected with a selectable self-replicating subgenomic hepatitis C virus (HCV) Genotype 1b [Clone ET] RNA sequence harboring a luciferase [Luc] reporter gene) and Huh-7-CMV-Luc (Huh-7 cells containing a CMV major immediate early promoter – Luc construct) were obtained from Heidelberg University Hospital (Heidelberg, Germany).
Authentication	Cell lines were not authenticated.
Mycoplasma contamination	All cell lines tested negative for mycoplasma contamination.
Commonly misidentified lines (See ICLAC register)	None of the commonly misidentified cell lines were used.

Animals and other research organisms

Policy information about studies involving animals; ARRIVE guidelines recommended for reporting animal research, and Sex and Gender in Research

Laboratory animals	At KU Leuven, AG129 mice (129/Sv mice deficient in both IFN-α/β and IFN-γ receptors) were used (females, 7-11 weeks old). Breeding couples of AG129 mice were purchased from Marshall BioResources and bred in-house. AT UTMB, AG129 mice (male and female,6–8 weeks old) were used. At BPRC, healthy, adult, male and female, Indian-origin rhesus monkeys were used with a minimum age of 5 years, and a minimum body weight of 7 kg. The animals were selected from the experimental stock from the self-sustainable BPRC colony. Healthy, adult, Indian strain rhesus macaques, males and females, at least 5 kg in weight, 4–10 years of age were selected for the study at WRAIR. The animals were U.S. colony bred and procured from Covance Research Products, Inc. (Alice, Texas). Male CD-1 mice, 27–33 g of body weight and around 6–8weeks old were purchased from Charles River Laboratories Germany (Sulzfeld).
Wild animals	No wild animals were used in the study.
Reporting on sex	Findings do not apply to one sex only.
Field-collected samples	No field-collected samples were used in the study.
Ethics oversight	For the studies in AG129 mice at the KU Leuven, housing conditions and experimental procedures were approved by the ethics committee of KU Leuven (licence P169/2011 and P047/2017) following institutional guidelines approved by the Federation of European Laboratory Animal Science Associations. For the studies using CD-1 mice at Janssen Pharmaceutica, housing conditions and experimental procedures were approved by the Ethics Committee on Animal Experiments of Janssen Pharmaceutica, Beerse, Belgium (license number LA1100119). Janssen Pharmaceutica is holding full AAALAC accreditation. UTMB is an Association for the Assessment and Accreditation of Laboratory and Care (AAALAC) International accredited facility. All procedures were reviewed and approved by the UTMB Institutional Animal Care and Use Committee. The studies were carried out in strict compliance with the recommendations of the Guide for the Care and Use of Laboratory animals published by the National Research Council. Studies in rhesus macaques at the Biomedical Primate Research Centre (BPRC, Association for the Assessment and Accreditation of Laboratory Animal Care [AAALAC] International accredited) in Rijswijk, were approved by appropriate national authorities (CCD, Central Committee for Animal Experiments; licence number AVD5020020172884) and by the institutional Animal Welfare Body (AWB). Studies in rhesus macaques at the Walter Reed Army Institute of Research (WRAIR) were approved by the WRAIR Institutional Animal Care and Use Committee (IACUC). Research was conducted in compliance with the Animal Welfare Act and other federal statutes and regulations pertaining to animals, and the work was performed in accordance with the principles stated in the Guide for the Care and Use of Laboratory Animals, the National Research Council. WRAIR is fully accredited by AAALAC.

Note that full information on the approval of the study protocol must also be provided in the manuscript.
